# Local Adaptation to Altitude Underlies Divergent Thermal Physiology in Tropical Killifishes of the Genus *Aphyosemion*

**DOI:** 10.1371/journal.pone.0054345

**Published:** 2013-01-22

**Authors:** David J. McKenzie, Guillan Estivales, Jon C. Svendsen, John F. Steffensen, Jean-François Agnèse

**Affiliations:** 1 UMR5554 Institut des Sciences de l'Evolution, Université Montpellier 2, Montpellier, France; 2 UMR5119 Ecologie des Systèmes Marins Côtiers, Université Montpellier 2, Montpellier, France; 3 Marine Biological Section, University of Copenhagen, Helsingør, Denmark; University of Manchester, United Kingdom

## Abstract

In watersheds of equatorial West Africa, monophyletic groups of killifish species (genus *Aphyosemion*) occur in discrete altitudinal ranges, low altitude species (LA, sea level to ∼350 m) or high altitude species (HA, 350 to 900 m). We investigated the hypothesis that local adaptation to altitude by the LA and HA species would be revealed as divergent effects of temperature on their physiological energetics. Two species from each group (mass ∼350 mg) were acclimated to 19, 25 and 28°C, with 19 and 28°C estimated to be outside the thermal envelope for LA or HA, respectively, in the wild. Wild-caught animals (F0 generation) were compared with animals raised in captivity at 25°C (F1 generation) to investigate the contribution of adaptation versus plasticity. Temperature significantly increased routine metabolic rate in all groups and generations. However, LA and HA species differed in the effects of temperature on their ability to process a meal. At 25°C, the specific dynamic action (SDA) response was completed within 8 h in all groups, but acclimation to temperatures beyond the thermal envelope caused profound declines in SDA performance. At 19°C, the LA required ∼14 h to complete the SDA, whereas the HA required only ∼7 h. The opposite effect was observed at 28°C. This effect was evident in both F0 and F1. Reaction norms for effects of temperature on SDA therefore revealed a trade-off, with superior performance at warmer temperatures by LA being associated with inferior performance at cooler temperatures, and vice-versa in HA. The data indicate that divergent physiological responses to temperature in the LA and HA species reflect local adaptation to the thermal regime in their habitat, and that local adaptation to one thermal environment trades off against performance in another.

## Introduction

Temperature is the abiotic factor which exerts the greatest influence on the physiology of ectotherms such as teleost fishes [1]. It is widely accepted that fishes have adapted genetically, over evolutionary time, to function optimally within the range of temperatures that they routinely encounter in their environment [2], [3]. As a result, metabolism, physiological performance and thermal tolerance can all exhibit differences among species or geographically separated populations of a single species, along thermal gradients [4]–[6]. Fish species which inhabit cooler climes should function better at low temperatures by comparison with individuals from warmer climes [7]–[9], and it has been argued that processes of local thermal adaptation can involve a trade-off, whereby evolution of improved performance at colder temperatures is associated with reduced performance at warmer temperatures [10], [11].

It has also been argued that ectotherms which have evolved within a limited thermal range will become highly specialised to function within that range, with the consequence that they function poorly at temperatures outside it [8], [11]–[13]. In particular, equatorial climes are characterised by limited seasonal temperature variations but they show thermal gradients with altitude, with mean temperature cooling by between 0.5 and 1.0°C every 100 meters [11]. Terrestrial ectotherms show clear evidence for local thermal adaptation as a function of altitude [14]–[16]. The thermal physiology of tropical freshwater fish species that occupy discrete altitudinal ranges has not been studied, but it seems highly probable that they have adapted to function within the thermal range of their habitat.

**Figure 1:**
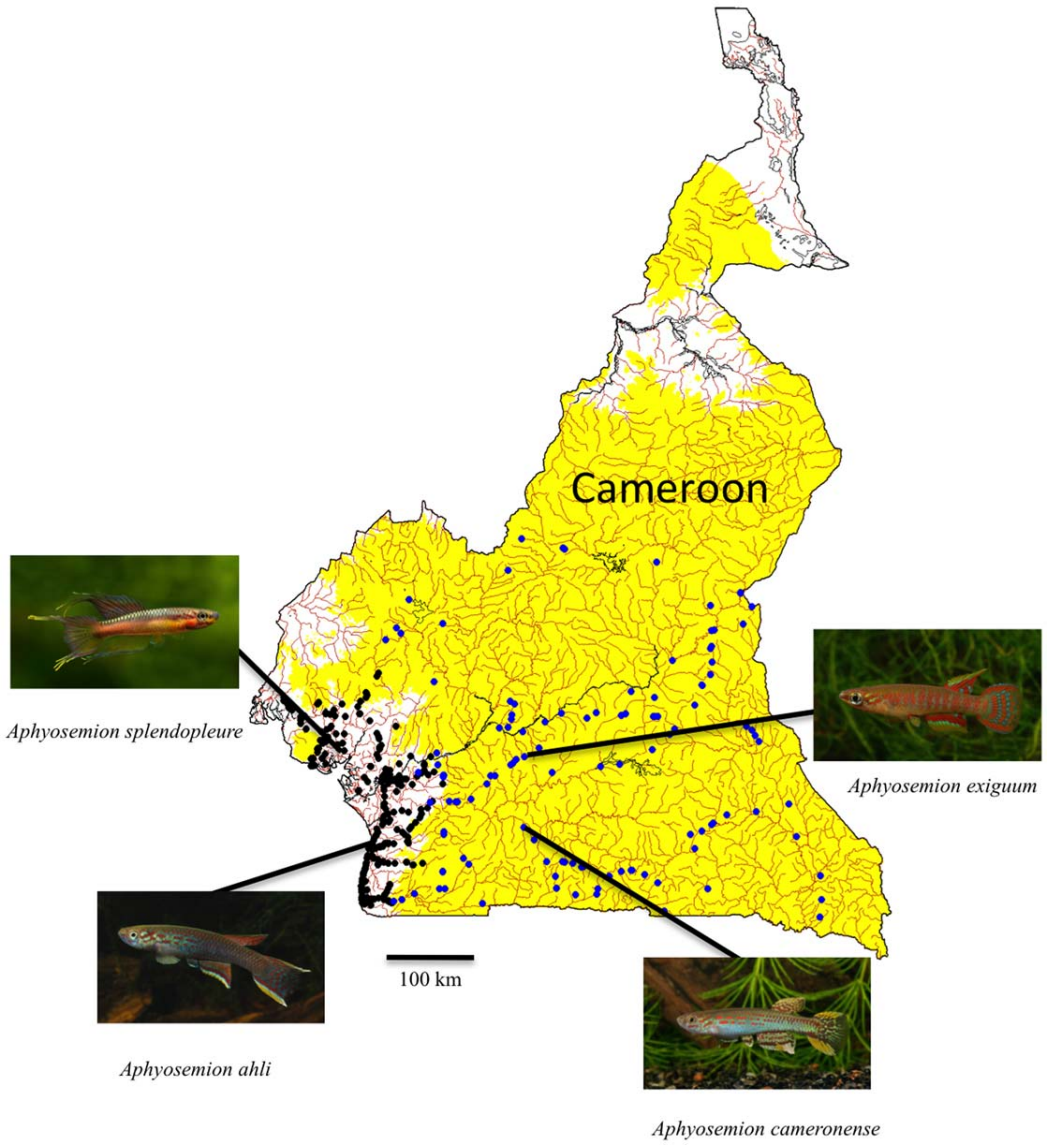
Map of Cameroon showing the distribution of the low altitude (LA) and high altitude (HA) *Aphyosemion* species. Altitudes below 350 m are in white, above 350 m in yellow. ,The LA species are almost exclusively present below 350 m (black dots), the HA species above 350 m (in blue). The arrows show where the animals were collected for the current study. Photos by Olivier Buisson (*A. ahli*, *A. cameronense* and *A. exiguum*) and Alf Persson (*A. splendopleure*).

*Aphyosemion* is a specious genus of African killifishes (Cyprinodontiformes) which, in equatorial forests of West Africa (Cameroon), comprise various species groups that arose several million years ago [17]–[19]. They inhabit small ponds and rivulets but the groups exhibit a clear distribution as a function of altitude (Figure 1): low altitude (LA) species are present in the coastal plain from sea level to an altitude of 350 meters, beyond which they are replaced by high altitude (HA) species, which occur between 350 and 900 meters [17]–[19]. The LA and HA groups are each monophyletic and have been separated for at least one million years [17]–[19]. These groups provide, therefore, an opportunity to investigate how adaptation to altitude has influenced the thermal physiology of tropical freshwater fishes.

Most ectotherms have some ability to acclimatise (or acclimate in the laboratory) to changes in their thermal environment, so individuals with different thermal histories can have different sensitivities to temperature [20]. Thus, in order to assess the contribution of adaptation to any measured physiological responses to temperature, it is necessary also to perform common-garden experiments, where species are reared under the same controlled thermal conditions, before their sensitivities are compared [11], [12].

The current study investigated the overall hypothesis that local adaptation to altitude by species from the LA and HA groups would be revealed as differences in the effects of temperature on their physiological energetics. Two species from each group (mass ∼350 mg) were acclimated to 19°C, 25°C and 28°C, with 19°C and 28°C estimated to be approximately 1.5°C beyond the thermal range experienced by LA or HA species, respectively, in the wild. Elements of their in-vivo physiological energetics were compared using techniques of respirometry [21], [22]. Rates of oxygen uptake were measured as an index of rates of routine energy use (“cost of living”). The specific dynamic action (SDA) response was measured as an indicator of the ability to process a meal, and its energetic costs. The SDA is the transient increase in oxygen uptake that follows feeding in all animals [23], it has ecological and evolutionary relevance because it reflects processes and costs of dietary protein handling and tissue protein turnover and deposition, hence growth [23]–[25]. Appetite was also measured, as reduced appetite is an indicator of sub-lethal physiological stress in fishes [26]. The potential existence of a trade-off was assessed by plotting the relationships between measured variables and acclimation temperature, to reveal whether LA and HA species exhibited opposing reaction norms [10], [11]. In order to explore the relative contribution of adaptation versus phenotypic plasticity, responses by wild-caught animals (F0 generation) were compared with those of animals raised under common garden conditions at 25°C in captivity (F1 generation). The data supported the hypothesis, the temperature reaction norms revealed a reciprocal trade-off in physiological performance as a function of acclimation temperature in both generations of the LA and HA groups, reflecting local adaptation to the thermal regime in their native habitat.

## Materials and Methods

### Ethics Statement

Animals were collected from Cameroon and transported by air to the Institute of Evolutionary Sciences of Montpellier (ISEM), France, with the fishing and export permit number 0020/ASE/MINEPIA/DIRPEC/SDARA. None of the species are on the IUCN Red List, or on CITES annex C. The fishes were held, and the non-lethal experiments were conducted, in strict accordance with the laws governing animal experimentation in France, under a licence (Expérimentation animale niv 1) held by D.J.M. At the end of the experiments, the fishes were maintained at ISEM, under the holding conditions described below.

### Animals

Four species of killifish were studied, *Aphysemion ahli* Myers, 1933 and *A. splendopleure* (Brüning, 1929) from LA, and *A. cameronense* (Boulenger, 1903) and *A. exiguum* (Boulenger, 1911) from HA. Wild individuals of both sexes and unknown age were captured from small ponds and rivulets in forested areas in Cameroon using dip nets. Specimens of *A. ahli* were captured from the surroundings of Kribi (N 02 59 115, E 09 59449, altitude 40 m), *A. splendopleure* from Tiko (N 04 04 273, E 09 21 794, altitude 26 m), *A. cameronense* from South East Ebolowa (N 02 42 310, E 10 48 474, altitude 624 m), *A. exiguun* from the surroundings of Yaoundé (N 03 51 590, E 11 26 139, altitude 745 m) (Figure 1).

After transfer to France and ISEM, the fishes were housed individually in tubs with a constant flow of biofiltered freshwater in a recirculating system at 25°C. Water temperature was regulated at 25°C with a 2 kW heater/cooler (TECO TR10, www.tecoonline.eu) and submersible 300 W aquarium heaters (EcoTherm, www.ecotherm.fr). Water in the system was exchanged at about 50% per week, using filtered (20 µm) reverse-osmosed water (<50 µs). The system was in a windowless room, photoperiod was maintained at 11 h day 13 h night, daybreak at 0800. All animals were fed on live *Artemia*, once every two days, to about 10% of their body mass. Animals were maintained under these conditions for at least 2 months prior to experimentation.

The F1 individuals were obtained by breeding wild animals in captivity. Breeding pairs (10 per species) were transferred to large tubs of static water at 25°C. Fertilized eggs were deposited every day on floating mats of acrylic wool. Eggs were removed daily and incubated in the same water as the breeders, at 25°C. Hatching occurred at 12 to 15 days. For their first two months, offspring were fed with *Artemia* nauplii, after which they were fed in the same manner as the adults, and maintained at 25°C. At between 6 and 8 months of age, the F1 fishes had reached adulthood. Therefore, eight groups of adult animals were studied, namely four species with wild and F1 individuals in each.

### Temperature acclimation

In order to establish a suitable spectrum of acclimation temperatures, water temperatures were measured daily for an annual cycle, using three dataloggers (Onset HOBO Pro v2 Water Temperature Data Logger, www.onsetcomp.com) placed in killifish habitats at two of the LA and HA sites, at an altitude of 28 m and 90 m or 513 m and 617 m respectively. At the LA sites, the overall annual mean (± S.D.) was 25.6±0.6°C, temperature never fell below 23.0°C and never exceeded 28.5°C. At the HA sites, the overall annual mean was 22.6±0.6°C, temperature never fell below 20.5°C and never exceeded 25.5°C. Air temperatures were obtained for a variety of altitudes in Cameroon [27], and showed a decline of approximately 0.6°C per 100 m increase in altitude. Therefore, assuming a similar temperature gradient in water over their altitudinal range, for LA temperature should rarely fall below 20.5°C at their upper altitudinal limit (350 m) and rarely exceed 29.0°C at their lower (sea-level) whereas, for HA, temperature should rarely fall below 18°C at their upper altitudinal limit (900 m) and and rarely exceed 26.5°C at their lower (350 m).

Consequently, groups of individuals were acclimated to two temperatures other than 25°C, a cooler temperature of 19°C, that was 1.5°C below the minimum expected for the LA groups and a warmer temperature of 28°C, hence 1.5°C above the maximum expected for the HA groups. Preliminary experiments found that more extreme temperatures, whether warmer (30°C) or cooler (15°C) caused some of the animals to refuse to feed. Water temperature was changed in the recirculating system at a rate of 1°C per day, using the 2 kW heater/cooler and submersible aquarium heaters. Animals were maintained for at least 14 days at the acclimation temperature prior to experimentation, and then at that temperature until all experiments had been completed, which could be up to 14 days. The minimum mean daily rate of temperature acclimation was therefore 0.3°C day^−1^ towards 19°C, and below 0.2°C day^−1^ towards 28°C, less than 0.5°C day^−1^ that is suggested to be sufficient for acclimation of biochemical and physiological processes in freshwater fishes [6], [28]. All animals fed spontaneously throughout the temperature acclimation and at their final temperature.

### Metabolic rate and specific dynamic action

Metabolic rate and SDA were measured by respirometry, as rates of O_2_ uptake [21], [22], [24], at all three temperatures. Measurements were made on four fish at a time. Forty-eight hours prior to experimentation, individuals were removed from their tubs, gently blotted dry on tissue paper and weighed to the nearest 0.1 mg. They were returned to their tubs and fasted until use in experiments.

For each fish, rates of O_2_ uptake were measured for 24 h in the fasted state, and for 24 h after consumption of a meal of live *Artemia* equivalent to 5% of the fish's body mass. These two measurements were made sequentially, with the order randomised among trials. When the animals were fasted first, they were gently coaxed into a 50 ml glass beaker underwater, in their tub. They were then gently poured into one of four respirometer chambers (internal diameter 19 mm, length 70 mm). The O_2_ uptake rate was then measured for 24 h, as described below. The fishes were then poured from their respirometer chamber into a small tub containing water at the appropriate temperature, and offered their meal of *Artemia*. They typically ate these within five min. Thirty min after being offered their ration, they were coaxed into the beaker underwater and poured gently back into their chamber and O_2_ uptake rate of the fed fish measured for 24 h. When the sequence of fasting/fed was reversed, the animals were offered their ration in their own tub then, 30 min later, transferred to the respirometer chamber as described above. Twenty four hours later they were removed and placed in the tub of water for 30 min without feeding, then returned to their chamber, as described above. Care was taken to avoid exposing the fish to air, to reduce stress during transfers [29]. In all cases, feeding and/or transfers were performed starting at 1600 h each day, to avoid any confounding effects of diurnal activity rhythms.

The respirometer chambers were submerged in 7 cm of water in a large plastic tub, which was provided with a flow of biofiltered water within a recirculating system, delivered by a submersible pump (Eheim 1024, www.eheim.com) from a reservoir, to which it returned by gravity. Water temperature in the entire system was regulated at ±0.1°C of the required temperature using a TempReg system (Loligo Systems, www.loligosystems.com), and aerated by aquarium air-pumps. Instantaneous O_2_ uptake rate was measured by intermittent stopped-flow respirometry [21] once every 30 min. Briefly, each rubber bung sealing a respirometry chamber was perforated by two glass tubes (internal diameter 2 mm). Two of these glass tubes, at opposing ends of the chamber, were connected to create a closed loop of gas-impermeable tygon tubing (internal diameter 2.9 mm) which passed over a multi-channel variable-speed roller pump (Watson-Marlow 323Du/4D, www.watson-marlow.com). The pump recirculated water through the loop and each chamber, with a total recirculating volume of approximately 35 ml in each case (this was measured to within 0.1 ml by gravimetry for each chamber). An O_2_-sensitive optode (Pre-sens Mini flow-thru, www.presens.de) was placed within this circuit, just downstream of the chamber exit, upstream of the pump. The optode was connected to a meter (Pre-Sens OXY-4 mini) that measured O_2_ content of the circuit, in mg l^−1^, continuously once every 15 s, with data displayed and stored on the manufacturer's dedicated software (Pre-Sens Oxyview). The other glass tube, at the same end of the chamber as the inflow from the roller pump, was connected to a small submersible pump (Sarlobetter Mini-A, www.sarlobetter.com.br) immersed in the water in the large outer tub. The remaining glass tube at the other end was connected to a small length of plastic tubing leading to the water surface. The submersible pumps were connected to timer, which turned them on for 15 min in every 30 min. When the submersible pumps were not on, there was a decline in O_2_ in the chamber due to uptake by the fish, which was recorded by the optode. The submersible pump then flushed the system with aerated water from the outer tub, after which the cycle was repeated. Disturbance was kept to a minimum during measurements. The water surface was shielded with opaque polystyrene to prevent visually disturbing the fish during any experimental activities. Rates of O_2_ uptake were then calculated as described previously [21], [22], in µg O_2_ g^−1^ fish mass h^−1^, considering the rate of decline in O_2_ content in the system during the closed period, the solubility of O_2_ at the test temperature, the volume of the system, and mass of the fish. When the fish was removed from the chamber between the two 24 h measurements, and at the end of each experiment, background respiration rates were measured over one 30 min cycle, and values corrected accordingly [22].

To correct for the effects on O_2_ uptake rate of differences in mass among individuals, all individual values were corrected to a common mass [30], using the general teleost allometric mass coefficient of 0.82 provided by [31]. A mass of 350 mg was chosen as it approximated mean mass of the individuals used in all treatments, and therefore oxygen uptake rates (Mo_2_) are reported in units of mg h^−1^, for a notional fish with that body mass.

Routine metabolic rate (RMR) during fasting was calculated as the overall mean of the Mo_2_ values for the last 16 h of measurements that followed recovery from sham feeding/handling stress in the fasted killifish [22]. The SDA response was calculated empirically for each individual, by subtracting the fasted Mo_2_ values from the fed values, respirometry cycle by cycle, and assuming that any difference was due to the SDA. The peak response (Mo_2peak_) was taken as the maximum difference between fed and fasted Mo_2_, and the time to this (T_peak_) were identified for each individual [22]. The total duration of the SDA (D_SDA_) was estimated as the time required to return to within 5% of fasted Mo_2_ for at least two consecutive measurements (i.e. 60 min). The total area of the SDA, the SDA coefficient (C_SDA_) was then calculated by integrating under the resulting curve [22], [23], [24].

### Appetite

Live *Artemia* were sorted for 1 mm body size with plankton sieves, and their average mass calculated based upon the mass of 5 groups of 20 individuals. Pilot experiments revealed that the killifish could eat up to 20% of their body mass in live *Artemia* before becoming satiated. Individual killifish whose mass had been measured as described above, were offered 25% of their mass as *Artemia*, and allowed to feed for 60 min. At the end of this period, the remaining *Artemia* were counted and their mass estimated, to calculate the mass eaten.

### Statistical analysis

Statistics were performed with Sigmaplot 11.0 (www.sigmaplot.com). In all cases, the level of statistical significance was taken as P<0.05. Data were checked for normality (Kolgorov-Smirnov test) and homogeneity of variance (Bartlett's test) prior to application of parametric tests, and in some cases were log-10 transformed to meet these requirements. If this still did not permit the use of parametric tests, data were compared by Kruskal-Wallis one-way ANOVA on ranks.

Mass of the individuals from each species, whether F0 or F1 generation, were compared by Kruskal-Wallis one-way ANOVA on ranks. Mean values for RMR, derived SDA variables or appetite, were each plotted against acclimation temperature for each of the four species, F0 and F1 separately, to reveal reaction norms. A two-way ANOVA, one factor being species, the other being temperature, was used to investigate significant differences among experimental groups for a given variable. In those cases where a significant difference was observed, Holm-Sidak post-hoc tests were undertaken to identify where in the dataset this difference occurred. If the two HA or LA species pairs in a generation did not differ significantly for a given variable, to facilitate comparisons and interpretation of results, they were combined into an LA or HA group, for F0 and F1 separately. Thus, there were four groups, LA F1 and F0 and the HA F1 and F0, each group comprising the appropriate species pair, *A. splendopleure* and *A. ahli* for LA and *A. cameronense* and *A. exiguum* for HA. These were then analysed in the two-way ANOVA with one factor being species group, the other being temperature.

## Results

The mean mass of the individuals studied for each species (F0 and F1 considered separately) at the three temperatures are shown in Table 1. There was some evidence of significant differences among the groups at the various temperatures, although absolute differences in mass were relatively limited (Table 1, Text S1). The potential effects of the allometric relationship between mass and metabolic rate in fishes [31] should, nonetheless, have been at least partially addressed by correcting values to a common mass [30] of 350 mg.

**Table 1:** Mean (± SD) mass of killifish studied at each of three acclimation temperatures (19, 25 and 28°C).

	low altitude				high altitude			
	*A. splendopleure*		*A. ahli*		*A. cameronense*		*A. exiguum*	
	F0	F1	F0	F1	F0	F1	F0	F1
19°C	400±65(6)	249±55(5)	267±151(6)	249±67(6)	331±134(5)	298±79(6)	358±169(6)	372±133(6)
25°C	400±52(6)	239±138(5)	316±61(5)	238±65(6)	337±154(5)	356±55(6)	264±32(5)	382±165(5)
28°C	327±63(7)	236±121(5)	263±145(6)	243±58(6)	362±103(6)	334±107(6)	245±61(6)	372±133(6)

Four species were studied, a pair from low altitude (*A. splendopleure* and *A. ahli*) and from high altitude (*A. cameronense* and *A. exiguum*), with two generations per species, an F0 generation captured in the wild and an F1 generation bred in captivity. Values are in mg, numbers in brackets are sample size. Data failed the Kolgorov-Smirnov test for normality, even after log transformation. Kruskal-Wallis one-way ANOVA by ranks found a significant difference (H = 46.552, d.f. = 23, P = 0.003) but Dunn's method for all pairwise multiple comparisons failed to identify any significant differences among groups (see Text S1 for details).

**Figure 2:**
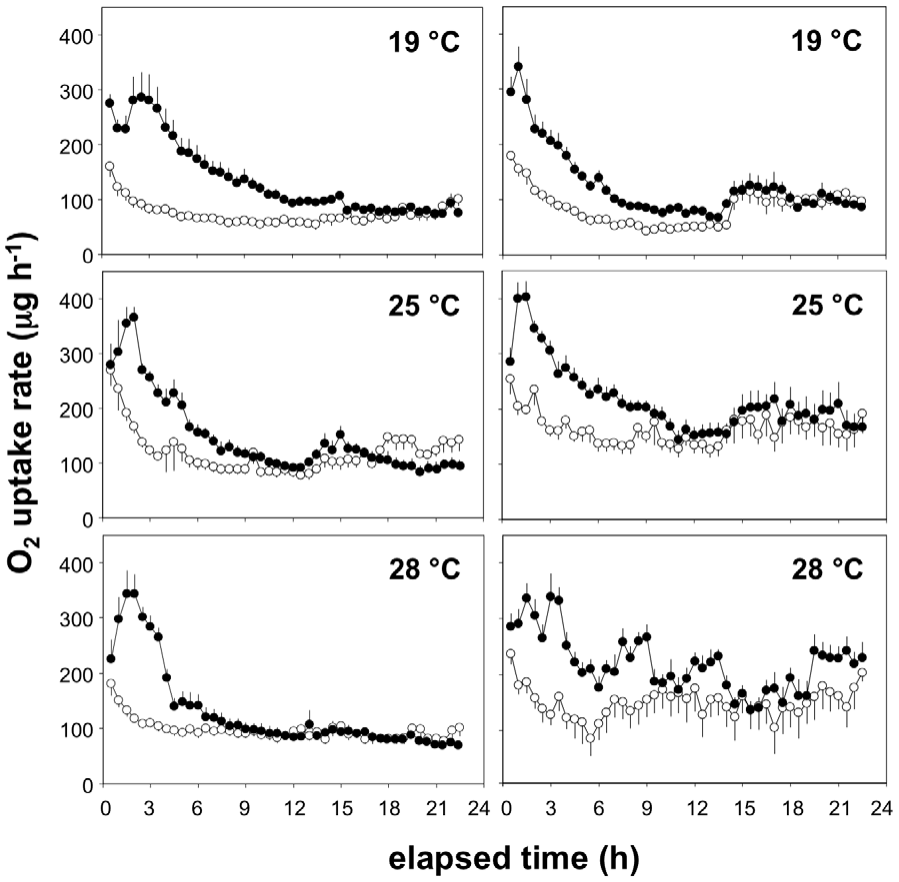
Mean (± SE) rates of oxygen uptake in wild-caught individuals of two species of killifish, *A. ahli* from low altitude (left column) or *A. exiguum* from high altitude (right column), at three different acclimation temperatures. In each panel, data are for fish placed into respirometers at 1630 either fasted (white symbols) or when fed 5% of their body mass as live *Artemia* (black symbols). Rates of oxygen uptake are corrected to a body mass of 350 mg, n = 6 in all cases. See text for further details.

**Figure 3:**
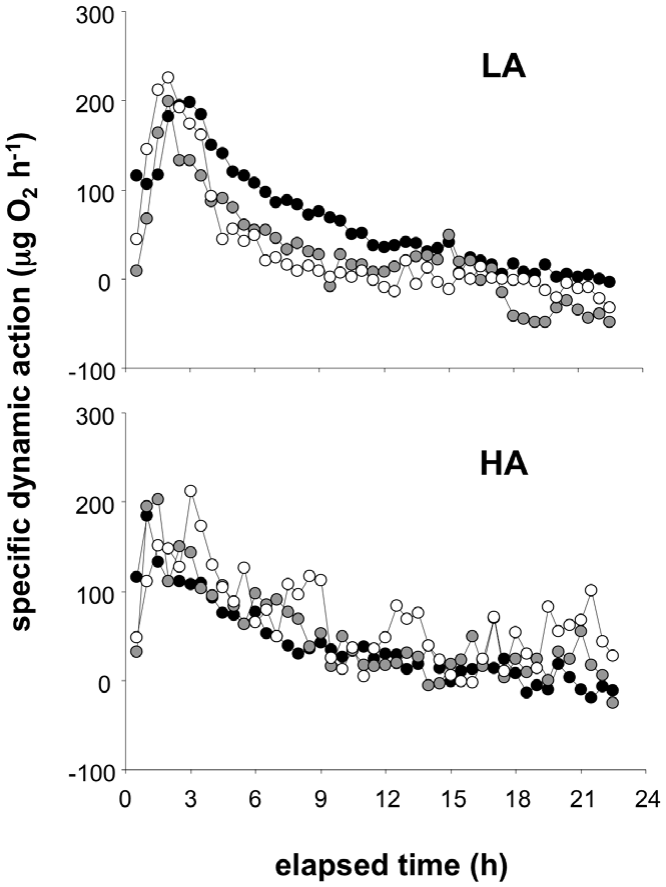
Specific dynamic action (SDA), expressed as rates of oxygen uptake, in two species of killifish, *A. ahli* from low altitude (LA, upper panel) or *A. exiguum* from high altitude (HA, lower panel), at three different acclimation temperatures, 19°C (white), 25°C (grey) and 28°C (black). The SDA was calculated as the net difference in oxygen uptake between animals that were either fasted, or fed 5% of their body mass as *Artemia* (see Figure 2). Values are means of n = 6 individuals from each species, rates are corrected to a body mass of 350 mg. Standard error bars are not shown to ease reading of the figure, but were approximately 10% of the mean value in all cases.

Figure 2 shows a representative trace of mean Mo_2_ over 24 h in two groups, namely an F0 generation of one LA and one HA species, at each of three acclimation temperatures. At all temperatures, the fasted traces clearly show that the manipulation at the start of the experiment caused elevated Mo_2_ but, by about 4 h afterwards, this effect had disappeared and Mo_2_ was relatively stable, particularly during the night, then showing increased metabolic rate presumably due to spontaneous activity when the lights came on at 0800 (Figure 2). The fed traces clearly show that mean Mo_2_ was higher than fasted Mo_2_ for a period that extended for a number of hours at each temperature (Figure 2). Figure 3 shows a representative trace of mean net SDA response over 24 h derived from the data shown in Figure 2, namely an F0 generation of one LA and one HA species, at each of three acclimation temperatures. There was a clear SDA response at all temperatures, and this was true for all of the experimental groups (Figure 3). Similar results were obtained in all experimental groups, and formed the basis for the derivation of RMR and the SDA reaction norms, and their statistical comparison amongst the groups.

An initial comparison of all species separately by two-way ANOVA, for all respirometric variables, found that the species pairs for LA and HA, considering F0 and F1 generations separately, never differed for any variable at any temperature, irrespective of any effects of temperature (see Text S2, S3, S4, S5, S6 for ANOVA outputs for RMR, Mo_2peak_, T_peak_, T_SDA_ and C_SDA_, respectively). Therefore, as presaged in the methods above, data are presented for four groups, LA F1 and F0 and the HA F1 and F0, each group comprising the appropriate species pair, *A. splendopleure* and *A. ahli* for LA and *A. cameronense* and *A. exiguum* for HA.

### Routine metabolic rate

Table 2 shows RMR at each temperature for the four groups. There were no differences in mean RMR among the groups at any given temperature. Increasing temperature caused an overall significant increase in RMR, when all groups were considered together. When considered separately, all groups exhibited a significantly lower RMR at 19°C compared to 28°C, and in some cases also compared to 25°C also (Table 2).

**Table 2:** Mean (± SE) routine metabolic rate (RMR) at three acclimation temperatures in four groups of killifish.

	low altitude		high altitude	
	F0	F1	F0	F1
19°C	76±9^§^	68±7^§^	81±5^†§^	63±11^†§^
25°C	92±8^§^	82±6^§^	125±12	95±10
28°C	138±11	122±7	146±22	120±6

Each group comprised a species pair from either low altitude (*A. splendopleure* and *A. ahli*) or from high altitude (*A. cameronense* and *A. exiguum*), with two generations per species, an F0 generation captured in the wild and an F1 generation bred in captivity. Metabolic rate is expressed as rates of oxygen uptake (µg h^−1^), corrected to a body mass of 350 mg, n = between 11 and 13. A 2-way ANOVA on log-10 transformed values found an effect of group (df = 3, F = 3.512, P = 0.017), temperature (df = 2, F = 37.637, P<0.001) but no interaction between group and temperature (df = 6, F = 0.24, P = 0.96). Overall, low altitude F1 had lower RMR than high altitude F0, and overall RMR differed at all temperatures. There were, however, no differences among groups at any temperature. Within each group, Holm-Sidak pairwise comparisons revealed significant effects of temperature (^†^, significantly different from 25°C; ^§^, significantly different from 28°C, P<0.05, see Text S7 for details).

### Specific dynamic action

**Figure 4:**
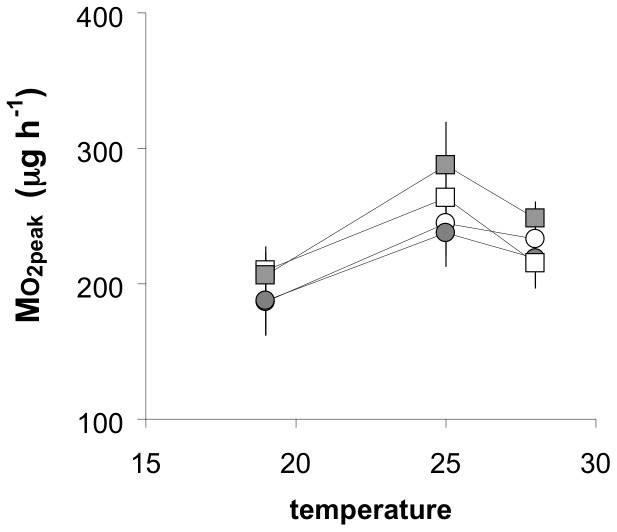
Reaction norms for mean (± SE) peak oxygen uptake rate during the specific dynamic action (Mo_2-peak_) as a function of acclimation temperature in four groups of killifish. Each group comprised a species pair from either low altitude (*A. splendopleure* and *A. ahli*), with both an F0 generation (white squares) and an F1 generation (grey squares), or from high altitude (*A. cameronense* and *A. exiguum*), with both an F0 generation (white circles) and an F1 generation (grey circles). F0 generation were captured in the wild, F1 generation were bred in captivity. Rates of oxygen uptake are corrected to a body mass of 350 mg, n = between 5 and 7. Two-way ANOVA found a significant effect of temperature (F = 6.064, d.f. = 2, P = 0.003) but not of group (F = 0.926, d.f. = 3, P = 0.430) or their interaction (F = 0.241, d.f. = 6, P = 0.962). This was due to higher overall peak at 25°C than at 19°C, although individual groups did not show significant effects (see Text S8 for details).

Figure 4 shows the reaction norms for mean Mo_2peak_ as a function of acclimation temperature in the eight groups. There were no clear differences in reaction norms among species groups. Overall, there was a significant effect of temperature, in that the peak was higher at 25°C than at 19°C. This effect was not, however, observed in any of the four groups when considered separately, there were no differences among the groups at any temperature, and no interaction between temperature and group. Figure 4 does show, however, that both F0 and F1 generations for both LA and HA had very similar mean peak values as a function of temperature.

**Figure 5:**
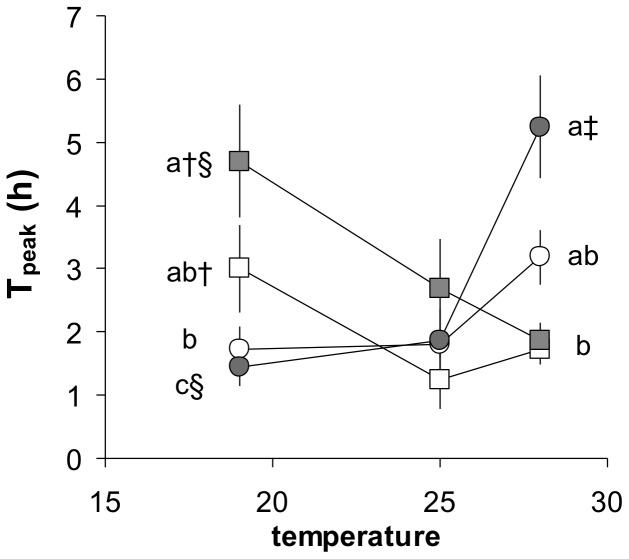
Reaction norms for mean (± SE) time required to reach peak oxygen uptake during the specific dynamic action (T_peak_) as a function of acclimation temperature in four groups of killifish. Each group comprised a species pair from either low altitude (*A. splendopleure* and *A. ahli*), with both an F0 generation (white squares) and an F1 generation (grey squares), or from high altitude (*A. cameronense* and *A. exiguum*), with both an F0 generation (white circles) and an F1 generation (grey circles). F0 generation were captured in the wild, F1 generation were bred in captivity, n = between 5 and 7. A 2-way ANOVA on log 10 transformed values found no effect of group (df = 3, F = 0.849, P = 0.470), but a significant effect of temperature (df = 2, F = 6.652, P = 0.002) and their interaction (df = 6, F = 6.015, P<0.001). Overall T_peak_ was lower at 25°C than at 19 or 28°C. At 19 and 28°C, a similar letter superscript indicates no significant difference among group means for that temperature. Within a group, † indicates a difference between 19 and 25°C, § indicates a difference between 19 and 28°C, ‡ indicates a significant difference between 25 and 28°C (see Text S9 for details).

Figure 5 shows the reaction norms for the effects of temperature on mean T_peak_ in the four groups. The two-way ANOVA revealed a significant effect of temperature, which overall was lower at 25°C than at the other two temperatures (Figure 5). There was also, however, a significant interaction between temperature and group, because the effects of temperature depended upon the group. This was evident from the reaction norms; T_peak_ tended to decrease with increasing temperature in LA, whereas it increased with temperature in HA (Figure 5). These effects of temperature were significant within the LA F0 and F1 groups, and in the HA F1 group. As a consequence, therefore, T_peak_ was significantly higher in LA groups compared to some HA groups at 19°C, and the opposite was true at 28°C. All groups had similar intermediate values at 25°C (Figure 5).

**Figure 6:**
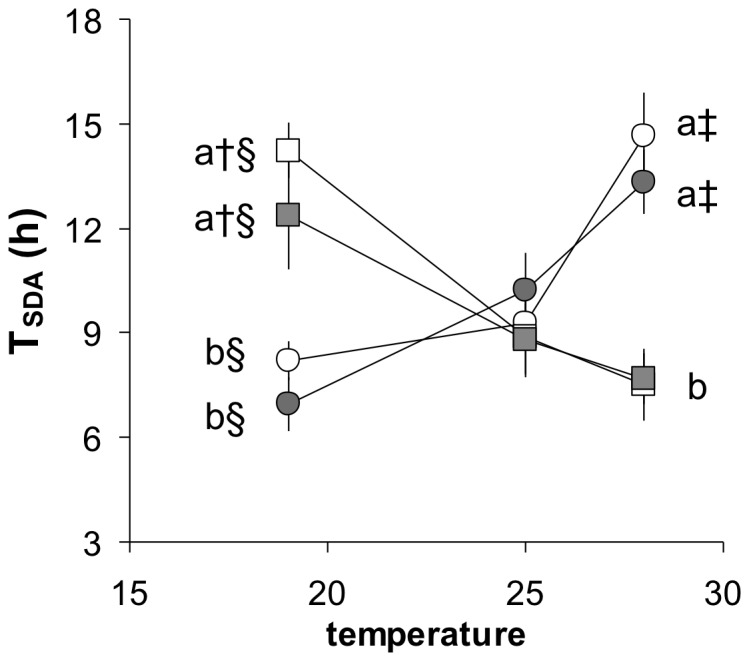
Reaction norms for mean (± SE) time required to complete the specific dynamic action response (T_SDA_) as a function of acclimation temperature in four groups of killifish. Each group comprised a species pair from either low altitude (*A. splendopleure* and *A. ahli*), with both an F0 generation (white squares) and an F1 generation (grey squares), or from high altitude (*A. cameronense* and *A. exiguum*), with both an F0 generation (white circles) and an F1 generation (grey circles). F0 generation were captured in the wild, F1 generation were bred in captivity, n = between 5 and 7. A 2-way ANOVA found no effect of group (df = 3, F = 0.676, P = 0.568) or temperature (df = 2, F = 2.064, P = 0.131) but a significant interaction (df = 6, F = 12.695, P<0.001). At 19 and 28°C, a similar letter superscript indicates no significant difference among group means. Within a group, † indicates a difference between 19 and 25°C, § indicates a difference between 19 and 28°C, ‡ indicates a significant difference between 25 and 28°C (see Text S10 for details).

Figure 6 shows the reaction norms for the effects of temperature on D_SDA_. The D_SDA_ varied between seven and 14 h but as for T_peak_, there was clear evidence of opposing temperature reaction norms in the LA and HA groups. The two-way ANOVA did not reveal any significant overall effect of temperature or group, but a significant interaction between the two factors. That is, as for T_peak_, the effect of temperature depended upon the group. The F0 and F1 generations showing very similar reaction norms within the altitude groups, and there were no significant differences between generations at any temperature. Both generations of the LA species pairs showed, however, a significantly shorter D_SDA_ at 28°C compared to both 25 and 19°C. The opposite was true of the HA species pairs, which both showed a significantly longer D_SDA_ at 28°C compared to both 19 and 25°C (Figure 6). Furthermore, both generations of LA had a shorter D_SDA_ at 28°C than both generations of HA, while the opposite was true at 19°C, with intermediate values at 25°C.

Table 3 shows the effects of temperature on C_SDA_ in the four groups. The data were variable amongst individuals at all temperatures, with no significant differences among or within groups.

**Table 3:** Mean (± SE) SDA coefficient, following consumption of 5% body mass as *Artemia*, at three acclimation temperatures in four groups of killifish.

	low altitude		high altitude	
	F0	F1	F0	F1
19°C	1127±114	999±210	649±75	653±99
25°C	1087±178	901±128	730±97	939±136
28°C	917±104	821±90	1129±343	1246±176

Each group comprised a species pair from either low altitude (*A. splendopleure* and *A. ahli*) or from high altitude (*A. cameronense* and *A. exiguum*), with two generations per species, an F0 generation captured in the wild and an F1 generation bred in captivity. The SDA coefficient is expressed as µg O_2_ consumed, corrected to a body mass of 350 mg, n = between 11 and 13. Two-way ANOVA on log 10 transformed data revealed no significant overall effects of group (df = 3, F = 1.739, P = 0.163), temperature (df = 2, F = 1.354, P = 0.262) or interaction between group and temperature (df = 6, F = 1.227, P = 0.297), therefore no differences among individual groups or temperatures (see Text S11 for details).

### Appetite

Table 4 shows the effects of temperature on four different species×generation groups, namely *A. ahli* F0 and *A. splendopleure* F1 for LA, and *A. exiguum* F0 and *A. cameronense* F1 for HA. There were no significant differences among any group; all individuals were able to consume about 15% of their body mass at any given temperature.

**Table 4:** Mean (± SE) appetite at three acclimation temperatures in four species of killifish, comprising an F0 generation of *A. ahli* and F1 generation of *A. splendopleure* from low altitude, or an F0 generation of *A. exiguum* and an F1 generation of *A. cameronense* from high altitude.

	low altitude		high altitude	
	*A. ahli* F0	*A. splendopleure* F1	*A. exiguum* F0	*A. cameronense* F1
19°C	15±1	18±2	19±3	14±2
25°C	14±2	19±4	14±2	19±3
28°C	15±2	24±3	18±2	17±2

Appetite is expressed as the percentage body mass of live *Artemia* consumed when fed a single meal *ad-libitum*, n = between 10 and 12. One-way ANOVA revealed no significant differences amongst groups (DF = 11, F = 1.279, P = 0.244; see Text S12 for details).

## Discussion

Our study indicates that local adaptation to altitude is associated with divergent thermal physiology in tropical freshwater fishes. Specifically, our data support the hypothesis that local thermal adaptation by ectotherms involves a trade-off whereby optimising performance at cooler temperatures is associated with reduced performance at warmer temperatures, and vice versa. This pattern emerged because of the divergent effects of temperature on SDA in the LA and HA species, in particular the speed at which meals were processed. The similarity of the reaction norms for the effects of temperature on SDA in F0 and F1 generations of both LA and HA groups indicates that the responses reflect local adaptation to different altitudes.

The absence of any differences between the LA and HA species in their temperature reaction norms for RMR indicates that both sets of species have similar thermal sensitivity for their enzymes of aerobic metabolism. Thus, local adaptation to different thermal regimes was not related to differences in routine maintenance costs. There is evidence that metabolic rate in fishes can be a stable individual trait which could potentially be subject to selection [32], [33]. It might be expected, therefore, that the species would exhibit thermal adaptation of metabolic enzymes, whereby HA animals would sustain a relatively higher metabolic rate at colder temperatures, offsetting depressive effects of cold, and LA animals would exhibit a relatively lower metabolic rate at warmer temperatures, offsetting the acceleratory effects of warmth [11]. It is not clear whether longer periods of acclimation to the cooler and warmer temperatures might have elicited more profound plastic responses, eventually leading to differences in the reaction norms for RMR [12], [20], [34].

It has been argued that, by accelerating biochemical processes in ectotherms, increasing acclimation (or acclimatisation) temperature should elicit a more rapid SDA response, with a shorter T_peak_ and D_SDA_, higher MO_2peak_ but no change in C_SDA_ [23]. This pattern was only partially observed in the current study. Thus, the T_peak_ and D_SDA_ data did reveal that LA animals were able to initiate the SDA response and process a standard ration more rapidly at warmer temperatures. The opposite was, however, true of the HA animals, which processed a meal more rapidly at cooler temperatures. Elements of digestive capacity have been used previously as indicators of thermal adaptation/acclimation in ectotherms [35]–[37]. This study demonstrates that the SDA response can be used as an indicator of local thermal adaptation in fishes, and demonstrates that such adaptation involves a trade-off [10], because the more rapid SDA of the LA species at warmer temperatures was associated with slower performance at cooler temperatures, and vice-versa for the HA species. The upper and lower temperatures used here were estimated to be only about 1.5°C below or above the thermal range that the LA and HA species, respectively, would expect to encounter over their altitudinal range in the wild, yet they caused a marked decline in SDA performance. The opposing temperature reaction norms between LA and HA species are consistent with the hypothesis that the species are stenothermal, each specialized to function within a narrow range of temperatures, losing function immediately outside this [11]. Further studies, at more temperatures, are required to describe the overall shape of the temperature performance curve for the species groups [11].

This interspecific altitudinal comparison differs from studies on fishes along temperate latitudinal gradients, which have tended to compare populations within coastal and/or euryhaline species [4]–[6], [37], [38]. These have not revealed stenothermal populations at each latitude, but a quite broad thermal tolerance typical of temperate ectotherms [11], [39] and extensive overlap of absolute thermal tolerance among populations over the range [6]. They have often found counter-gradient variation, where cold-adapted populations at high latitudes exhibit more rapid digestion times and growth rates than warm adapted conspecifics at lower latitudes, which can trade-off against other fitness functions such as locomotor performance [37], [38].

Proximate mechanisms of thermal adaptation can involve changes in the thermal sensitivity of proteins, in particular of enzymes, and modification of membrane composition and function [11]. At a systemic level, thermal adaptation can involve adaptation by elements of the respiratory transport chain, with evidence that fishes exhibit their maximum aerobic metabolic scope, the scope to perform aerobic activities, within the range of temperatures at which they have evolved [3]. It has been proposed that thermal tolerance in fishes is defined by limitations to aerobic metabolic scope and systemic oxygen delivery at non-optimal temperatures [40], [41]. The decline in SDA performance seen in the LA and HA groups at 19°C and 28°C, respectively, were not associated with any reduction in MO_2peak_, so the data do not provide evidence that the effects were due to limitations in oxygen delivery. The absence of any differences in appetite in members of the four species at the three acclimation temperatures, although only a partial dataset, indicates that the animals did not differ in their desire to feed. It is well-known that reduced appetite is a sensitive indicator of physiological stress in fishes [26]. Therefore, the data do not provide evidence of major physiological impairment and stress at the non-optimal temperatures but, rather, of differences in routine physiological performance. The fact that reaction norms for C_SDA_ did not differ indicates that the reduced SDA performance was not related to different total metabolic costs of processing the meal, but only in the rapidity with which this occurred [22].

The decline in SDA performance may, therefore, reflect effects of local thermal adaptation of function of the digestive tract, for example digestive enzymes, processes of absorption, and then the metabolic pathways of nutrient assimilation. A significant proportion of the SDA response is believed to reflect the metabolic costs of tissue protein turnover and deposition, using the amino acids consumed in the meal [25]. Thus, these processes of digestion and assimilation should be targeted in future studies to explore which elements have been subject to selection during local adaptation to altitude and therefore the mechanistic basis of the trade-off in performance between the LA and HA species. There is evidence in a number of fish species that a more rapid SDA response is related to a more rapid growth rate [22], [42], presumably because it allows animals to feed more frequently. There are a number of potential ecological advantages to growing rapidly, in particular in juvenile stages in an environment that is not food-limited [22], [42], [43].

The current dataset cannot distinguish whether the differences in thermal physiology are a cause or a consequence of the species' distribution [39]. The two groups are each monophyletic and diverged at least one million years ago [17]–[19]. In the wild, they are not separated by a geographical barrier at 350 m. They have a parapatric distribution, in the rainforests one species group replaces the other with an abrupt transition from one rivulet to the next, they are never found in syntopy [17]–[19]. The differences in the groups' abilities to process meals as a function of temperature may contribute to maintaining the distribution of the LA and HA species at their altitudes, for example by providing competitive advantages to rapidly growing juveniles in the thermal range to which they are adapted.

Investigating physiological responses to temperature in ectotherms, in particular the extent to which these are genetically fixed and phenotypically plastic, is now of interest for predicting the effects of climate change and global warming on biodiversity [44], [45]. Many equatorial areas are biodiversity hotspots for freshwater fish fauna [46], but relatively little is known about their patterns of thermal tolerance [47] and the potential impact of global warming [45]. The current dataset indicates that fishes that occupy discrete altitudinal ranges in the tropics may be highly stenothermal, physiological performance may be particularly finely adapted to the thermal regime in their habitat. For both LA and HA groups, exposure to temperatures beyond the apparent thermal envelope in their habitats caused profound declines in function. Thus, small variations in temperature, for example due to global warming, could have important implications for the distribution of such species, in particular because their freshwater habitats may have significant barriers to dispersal [46].

## Supporting Information

Text S1 **Kruskal-Wallis One Way Analysis of Variance on Ranks comparing body mass of killifish (genus Aphyosemion) individuals from two species from two altitudes, and two generations, studied at three temperatures (19°, 25° and 28°C).**
(DOC)

Text S2 **Two Way Analysis of Variance comparing routine metabolic rate at three temperatures among 2 altitudes×2 species×2 generations.**
(DOC)

Text S3 **Two Way Analysis of Variance comparing peak SDA MO2 at three temperatures among 2 altitudes×2 species×2 generations.**
(DOC)

Text S4 **Two Way Analysis of Variance comparing time to SDA peak at three temperatures among 2 altitudes×2 species×2 generations.**
(DOC)

Text S5 **Two Way Analysis of Variance comparing SDA duration at three temperatures among 2 altitudes×2 species×2 generations.**
(DOC)

Text S6 **Two Way Analysis of Variance comparing SDA coefficient at three temperatures among 2 altitudes×2 species×2 generations.**
(DOC)

Text S7 **Two Way Analysis of Variance. comparing routine metabolic rate at three temperatures among 2 altitude groups×2 generations.**
(DOC)

Text S8 **Two Way Analysis of Variance comparing peak SDA MO2 at three temperatures among 2 altitude groups×2 generations.**
(DOC)

Text S9 **Two Way Analysis of Variance comparing time to SDA peak at three temperatures among 2 altitude groups×2 generations.**
(DOC)

Text S10 **Two Way Analysis of Variance comparing SDA duration at three temperatures among 2 altitude groups×2 generations.**
(DOC)

Text S11 **Two Way Analysis of Variance comparing SDA coefficient at three temperatures among 2 altitude groups×2 generations.**
(DOC)

Text S12 **One Way Analysis of Variance comparing appetite among 2 high altitude and 2 low altitude species.**
(DOC)
