# Supplementary material for: Local Adaptation to Altitude Underlies Divergent Thermal Physiology in Tropical Killifishes of the Genus Aphyosemion
Source: PLoS One. 2013 Jan 22;8(1):e54345. doi: 10.1371/journal.pone.0054345 (PMC3551936; doi:10.1371/journal.pone.0054345)
Supplement: Text S1 — Kruskal-Wallis One Way Analysis of Variance on Ranks comparing body mass of killifish (genus Aphyosemion) individuals from two species from two altitudes, and two generations, studied at three temperatures (19°, 25° and 28°C). (DOC) [file pone.0054345.s001.doc]

**Supporting Information 1**

**Kruskal-Wallis One Way Analysis of Variance on Ranks. comparing body mass of killifish (genus Aphyosemion) individuals from two species from two altitudes, and two generations, studied at three temperatures (19°, 25° and 28° C)**

**HA, high altitude; LA, low altitude; F0, wild; F1, raised in common garden; A., genus Aphyosemion,**

Dependent Variable: body mass in mg

**Group N Missing Median 25% 75%**

HA F0 A. exiguum 19 6 0 285.000 263.000 468.000

HA F0 A. exiguum 25 5 0 270.000 233.750 287.750

HA F0 A. exiguum 28 6 0 253.500 226.000 286.000

HA F1 A. exiguum 19 6 0 365.000 300.000 405.000

HA F1 A. exiguum 25 5 0 345.000 200.000 438.750

HA F1 A. exiguum 28 6 0 365.000 300.000 405.000

HA F0 A. cameronense 25 5 0 333.000 238.250 432.500

HA F0 A. cameronense 19 5 0 333.000 238.250 408.500

HA F0 A. cameronense 28 5 0 378.000 296.750 432.500

HA F1 A. cameronense 25 6 0 356.000 310.000 408.000

HA F1 A. cameronense 19 6 0 305.000 248.000 331.000

HA F1 A. cameronense 28 6 0 288.500 247.000 467.000

LA F0 A. ahli 19 6 0 353.000 255.000 519

LA F0 A. ahli 25 5 0 428 394.500 497.500

LA F0 A. ahli 28 6 0 498.000 384.000 692.000

LA F1 A. ahli 19 6 0 251.500 233.000 285.000

LA F1 A. ahli 25 6 0 238.000 223.000 260.000

LA F1 A. ahli 28 7 0 243.000 235.500 278.750

LA F0 A. splendopleure 19 5 0 280.000 222.750 302.500

LA F0 A. splendopleure 25 5 0 280.000 232.750 302.500

LA F0 A. splendopleure 28 5 0 237.000 216.250 313.750

LA F1 A. splendopleure 25 6 0 415.500 320.000 479.000

LA F1 A. splendopleure 19 6 0 389.000 350.000 425

LA F1 A. splendopleure 28 6 0 268.000 210.000 390.000

H = 45.597 with 23 degrees of freedom. (P = 0.003)

The differences in the median values among the treatment groups are greater than would be expected by chance; there is a statistically significant difference (P = 0.003)

To isolate the group or groups that differ from the others use a multiple comparison procedure.

All Pairwise Multiple Comparison Procedures (Dunn's Method) :

**Comparison Diff of Ranks Q P<0.05**

LA F0 A. ahli vs LA F1 A. ahli 82.083 3.608 No

LA F0 A. ahli vs LA F1 A. ahli 78.595 3.585 Do Not Test

LA F0 A. ahli vs HA F0 A. exig 77.167 3.392 Do Not Test

LA F0 A. ahli vs LA F1 A. ahli 76.000 3.341 Do Not Test

LA F0 A. ahli vs LA F0 A. sple 73.967 3.100 Do Not Test

LA F0 A. ahli vs HA F0 A. exig 71.767 3.008 Do Not Test

LA F0 A. ahli vs LA F0 A. sple 70.367 2.949 Do Not Test

LA F0 A. ahli vs LA F0 A. sple 67.267 2.819 Do Not Test

LA F0 A. ahli vs LA F1 A. sple 60.000 2.637 Do Not Test

LA F0 A. ahli vs HA F1 A. came 57.583 2.531 Do Not Test

LA F0 A. ahli vs HA F1 A. exig 50.167 2.103 Do Not Test

LA F0 A. ahli vs HA F0 A. came 48.267 2.023 Do Not Test

LA F0 A. ahli vs HA F0 A. exig 46.917 2.062 Do Not Test

LA F0 A. ahli vs HA F1 A. came 46.667 2.051 Do Not Test

LA F0 A. ahli vs HA F0 A. came 45.867 1.922 Do Not Test

LA F0 A. ahli vs LA F0 A. ahli 36.500 1.604 Do Not Test

LA F0 A. ahli vs HA F1 A. exig 35.250 1.549 Do Not Test

LA F0 A. ahli vs HA F1 A. exig 35.250 1.549 Do Not Test

LA F0 A. ahli vs HA F0 A. came 35.167 1.474 Do Not Test

LA F0 A. ahli vs HA F1 A. came 34.667 1.524 Do Not Test

LA F0 A. ahli vs LA F1 A. sple 26.417 1.161 Do Not Test

LA F0 A. ahli vs LA F1 A. sple 17.250 0.758 Do Not Test

LA F0 A. ahli vs LA F0 A. ahli 6.367 0.267 Do Not Test

LA F0 A. ahli vs LA F1 A. ahli 75.717 3.173 Do Not Test

LA F0 A. ahli vs LA F1 A. ahli 72.229 3.130 Do Not Test

LA F0 A. ahli vs HA F0 A. exig 70.800 2.967 Do Not Test

LA F0 A. ahli vs LA F1 A. ahli 69.633 2.918 Do Not Test

LA F0 A. ahli vs LA F0 A. sple 67.600 2.713 Do Not Test

LA F0 A. ahli vs HA F0 A. exig 65.400 2.624 Do Not Test

LA F0 A. ahli vs LA F0 A. sple 64.000 2.568 Do Not Test

LA F0 A. ahli vs LA F0 A. sple 60.900 2.444 Do Not Test

LA F0 A. ahli vs LA F1 A. sple 53.633 2.248 Do Not Test

LA F0 A. ahli vs HA F1 A. came 51.217 2.147 Do Not Test

LA F0 A. ahli vs HA F1 A. exig 43.800 1.758 Do Not Test

LA F0 A. ahli vs HA F0 A. came 41.900 1.681 Do Not Test

LA F0 A. ahli vs HA F0 A. exig 40.550 1.699 Do Not Test

LA F0 A. ahli vs HA F1 A. came 40.300 1.689 Do Not Test

LA F0 A. ahli vs HA F0 A. came 39.500 1.585 Do Not Test

LA F0 A. ahli vs LA F0 A. ahli 30.133 1.263 Do Not Test

LA F0 A. ahli vs HA F1 A. exig 28.883 1.211 Do Not Test

LA F0 A. ahli vs HA F1 A. exig 28.883 1.211 Do Not Test

LA F0 A. ahli vs HA F0 A. came 28.800 1.156 Do Not Test

LA F0 A. ahli vs HA F1 A. came 28.300 1.186 Do Not Test

LA F0 A. ahli vs LA F1 A. sple 20.050 0.840 Do Not Test

LA F0 A. ahli vs LA F1 A. sple 10.883 0.456 Do Not Test

LA F1 A. sple vs LA F1 A. ahli 64.833 2.850 Do Not Test

LA F1 A. sple vs LA F1 A. ahli 61.345 2.798 Do Not Test

LA F1 A. sple vs HA F0 A. exig 59.917 2.634 Do Not Test

LA F1 A. sple vs LA F1 A. ahli 58.750 2.582 Do Not Test

LA F1 A. sple vs LA F0 A. sple 56.717 2.377 Do Not Test

LA F1 A. sple vs HA F0 A. exig 54.517 2.285 Do Not Test

LA F1 A. sple vs LA F0 A. sple 53.117 2.226 Do Not Test

LA F1 A. sple vs LA F0 A. sple 50.017 2.096 Do Not Test

LA F1 A. sple vs LA F1 A. sple 42.750 1.879 Do Not Test

LA F1 A. sple vs HA F1 A. came 40.333 1.773 Do Not Test

LA F1 A. sple vs HA F1 A. exig 32.917 1.380 Do Not Test

LA F1 A. sple vs HA F0 A. came 31.017 1.300 Do Not Test

LA F1 A. sple vs HA F0 A. exig 29.667 1.304 Do Not Test

LA F1 A. sple vs HA F1 A. came 29.417 1.293 Do Not Test

LA F1 A. sple vs HA F0 A. came 28.617 1.199 Do Not Test

LA F1 A. sple vs LA F0 A. ahli 19.250 0.846 Do Not Test

LA F1 A. sple vs HA F1 A. exig 18.000 0.791 Do Not Test

LA F1 A. sple vs HA F1 A. exig 18.000 0.791 Do Not Test

LA F1 A. sple vs HA F0 A. came 17.917 0.751 Do Not Test

LA F1 A. sple vs HA F1 A. came 17.417 0.766 Do Not Test

LA F1 A. sple vs LA F1 A. sple 9.167 0.403 Do Not Test

LA F1 A. sple vs LA F1 A. ahli 55.667 2.447 Do Not Test

LA F1 A. sple vs LA F1 A. ahli 52.179 2.380 Do Not Test

LA F1 A. sple vs HA F0 A. exig 50.750 2.231 Do Not Test

LA F1 A. sple vs LA F1 A. ahli 49.583 2.180 Do Not Test

LA F1 A. sple vs LA F0 A. sple 47.550 1.993 Do Not Test

LA F1 A. sple vs HA F0 A. exig 45.350 1.901 Do Not Test

LA F1 A. sple vs LA F0 A. sple 43.950 1.842 Do Not Test

LA F1 A. sple vs LA F0 A. sple 40.850 1.712 Do Not Test

LA F1 A. sple vs LA F1 A. sple 33.583 1.476 Do Not Test

LA F1 A. sple vs HA F1 A. came 31.167 1.370 Do Not Test

LA F1 A. sple vs HA F1 A. exig 23.750 0.995 Do Not Test

LA F1 A. sple vs HA F0 A. came 21.850 0.916 Do Not Test

LA F1 A. sple vs HA F0 A. exig 20.500 0.901 Do Not Test

LA F1 A. sple vs HA F1 A. came 20.250 0.890 Do Not Test

LA F1 A. sple vs HA F0 A. came 19.450 0.815 Do Not Test

LA F1 A. sple vs LA F0 A. ahli 10.083 0.443 Do Not Test

LA F1 A. sple vs HA F1 A. exig 8.833 0.388 Do Not Test

LA F1 A. sple vs HA F1 A. exig 8.833 0.388 Do Not Test

LA F1 A. sple vs HA F0 A. came 8.750 0.367 Do Not Test

LA F1 A. sple vs HA F1 A. came 8.250 0.363 Do Not Test

HA F1 A. came vs LA F1 A. ahli 47.417 2.084 Do Not Test

HA F1 A. came vs LA F1 A. ahli 43.929 2.004 Do Not Test

HA F1 A. came vs HA F0 A. exig 42.500 1.868 Do Not Test

HA F1 A. came vs LA F1 A. ahli 41.333 1.817 Do Not Test

HA F1 A. came vs LA F0 A. sple 39.300 1.647 Do Not Test

HA F1 A. came vs HA F0 A. exig 37.100 1.555 Do Not Test

HA F1 A. came vs LA F0 A. sple 35.700 1.496 Do Not Test

HA F1 A. came vs LA F0 A. sple 32.600 1.366 Do Not Test

HA F1 A. came vs LA F1 A. sple 25.333 1.114 Do Not Test

HA F1 A. came vs HA F1 A. came 22.917 1.007 Do Not Test

HA F1 A. came vs HA F1 A. exig 15.500 0.650 Do Not Test

HA F1 A. came vs HA F0 A. came 13.600 0.570 Do Not Test

HA F1 A. came vs HA F0 A. exig 12.250 0.538 Do Not Test

HA F1 A. came vs HA F1 A. came 12.000 0.527 Do Not Test

HA F1 A. came vs HA F0 A. came 11.200 0.469 Do Not Test

HA F1 A. came vs LA F0 A. ahli 1.833 0.0806 Do Not Test

HA F1 A. came vs HA F1 A. exig 0.583 0.0256 Do Not Test

HA F1 A. came vs HA F1 A. exig 0.583 0.0256 Do Not Test

HA F1 A. came vs HA F0 A. came 0.500 0.0210 Do Not Test

HA F0 A. came vs LA F1 A. ahli 46.917 1.966 Do Not Test

HA F0 A. came vs LA F1 A. ahli 43.429 1.882 Do Not Test

HA F0 A. came vs HA F0 A. exig 42.000 1.760 Do Not Test

HA F0 A. came vs LA F1 A. ahli 40.833 1.711 Do Not Test

HA F0 A. came vs LA F0 A. sple 38.800 1.557 Do Not Test

HA F0 A. came vs HA F0 A. exig 36.600 1.469 Do Not Test

HA F0 A. came vs LA F0 A. sple 35.200 1.412 Do Not Test

HA F0 A. came vs LA F0 A. sple 32.100 1.288 Do Not Test

HA F0 A. came vs LA F1 A. sple 24.833 1.041 Do Not Test

HA F0 A. came vs HA F1 A. came 22.417 0.939 Do Not Test

HA F0 A. came vs HA F1 A. exig 15.000 0.602 Do Not Test

HA F0 A. came vs HA F0 A. came 13.100 0.526 Do Not Test

HA F0 A. came vs HA F0 A. exig 11.750 0.492 Do Not Test

HA F0 A. came vs HA F1 A. came 11.500 0.482 Do Not Test

HA F0 A. came vs HA F0 A. came 10.700 0.429 Do Not Test

HA F0 A. came vs LA F0 A. ahli 1.333 0.0559 Do Not Test

HA F0 A. came vs HA F1 A. exig 0.0833 0.00349 Do Not Test

HA F0 A. came vs HA F1 A. exig 0.0833 0.00349 Do Not Test

HA F1 A. exig vs LA F1 A. ahli 46.833 2.059 Do Not Test

HA F1 A. exig vs LA F1 A. ahli 43.345 1.977 Do Not Test

HA F1 A. exig vs HA F0 A. exig 41.917 1.843 Do Not Test

HA F1 A. exig vs LA F1 A. ahli 40.750 1.791 Do Not Test

HA F1 A. exig vs LA F0 A. sple 38.717 1.623 Do Not Test

HA F1 A. exig vs HA F0 A. exig 36.517 1.530 Do Not Test

HA F1 A. exig vs LA F0 A. sple 35.117 1.472 Do Not Test

HA F1 A. exig vs LA F0 A. sple 32.017 1.342 Do Not Test

HA F1 A. exig vs LA F1 A. sple 24.750 1.088 Do Not Test

HA F1 A. exig vs HA F1 A. came 22.333 0.982 Do Not Test

HA F1 A. exig vs HA F1 A. exig 14.917 0.625 Do Not Test

HA F1 A. exig vs HA F0 A. came 13.017 0.546 Do Not Test

HA F1 A. exig vs HA F0 A. exig 11.667 0.513 Do Not Test

HA F1 A. exig vs HA F1 A. came 11.417 0.502 Do Not Test

HA F1 A. exig vs HA F0 A. came 10.617 0.445 Do Not Test

HA F1 A. exig vs LA F0 A. ahli 1.250 0.0549 Do Not Test

HA F1 A. exig vs HA F1 A. exig 0.000 0.000 Do Not Test

HA F1 A. exig vs LA F1 A. ahli 46.833 2.059 Do Not Test

HA F1 A. exig vs LA F1 A. ahli 43.345 1.977 Do Not Test

HA F1 A. exig vs HA F0 A. exig 41.917 1.843 Do Not Test

HA F1 A. exig vs LA F1 A. ahli 40.750 1.791 Do Not Test

HA F1 A. exig vs LA F0 A. sple 38.717 1.623 Do Not Test

HA F1 A. exig vs HA F0 A. exig 36.517 1.530 Do Not Test

HA F1 A. exig vs LA F0 A. sple 35.117 1.472 Do Not Test

HA F1 A. exig vs LA F0 A. sple 32.017 1.342 Do Not Test

HA F1 A. exig vs LA F1 A. sple 24.750 1.088 Do Not Test

HA F1 A. exig vs HA F1 A. came 22.333 0.982 Do Not Test

HA F1 A. exig vs HA F1 A. exig 14.917 0.625 Do Not Test

HA F1 A. exig vs HA F0 A. came 13.017 0.546 Do Not Test

HA F1 A. exig vs HA F0 A. exig 11.667 0.513 Do Not Test

HA F1 A. exig vs HA F1 A. came 11.417 0.502 Do Not Test

HA F1 A. exig vs HA F0 A. came 10.617 0.445 Do Not Test

HA F1 A. exig vs LA F0 A. ahli 1.250 0.0549 Do Not Test

LA F0 A. ahli vs LA F1 A. ahli 45.583 2.004 Do Not Test

LA F0 A. ahli vs LA F1 A. ahli 42.095 1.920 Do Not Test

LA F0 A. ahli vs HA F0 A. exig 40.667 1.788 Do Not Test

LA F0 A. ahli vs LA F1 A. ahli 39.500 1.736 Do Not Test

LA F0 A. ahli vs LA F0 A. sple 37.467 1.570 Do Not Test

LA F0 A. ahli vs HA F0 A. exig 35.267 1.478 Do Not Test

LA F0 A. ahli vs LA F0 A. sple 33.867 1.419 Do Not Test

LA F0 A. ahli vs LA F0 A. sple 30.767 1.289 Do Not Test

LA F0 A. ahli vs LA F1 A. sple 23.500 1.033 Do Not Test

LA F0 A. ahli vs HA F1 A. came 21.083 0.927 Do Not Test

LA F0 A. ahli vs HA F1 A. exig 13.667 0.573 Do Not Test

LA F0 A. ahli vs HA F0 A. came 11.767 0.493 Do Not Test

LA F0 A. ahli vs HA F0 A. exig 10.417 0.458 Do Not Test

LA F0 A. ahli vs HA F1 A. came 10.167 0.447 Do Not Test

LA F0 A. ahli vs HA F0 A. came 9.367 0.393 Do Not Test

HA F0 A. came vs LA F1 A. ahli 36.217 1.518 Do Not Test

HA F0 A. came vs LA F1 A. ahli 32.729 1.419 Do Not Test

HA F0 A. came vs HA F0 A. exig 31.300 1.312 Do Not Test

HA F0 A. came vs LA F1 A. ahli 30.133 1.263 Do Not Test

HA F0 A. came vs LA F0 A. sple 28.100 1.128 Do Not Test

HA F0 A. came vs HA F0 A. exig 25.900 1.039 Do Not Test

HA F0 A. came vs LA F0 A. sple 24.500 0.983 Do Not Test

HA F0 A. came vs LA F0 A. sple 21.400 0.859 Do Not Test

HA F0 A. came vs LA F1 A. sple 14.133 0.592 Do Not Test

HA F0 A. came vs HA F1 A. came 11.717 0.491 Do Not Test

HA F0 A. came vs HA F1 A. exig 4.300 0.173 Do Not Test

HA F0 A. came vs HA F0 A. came 2.400 0.0963 Do Not Test

HA F0 A. came vs HA F0 A. exig 1.050 0.0440 Do Not Test

HA F0 A. came vs HA F1 A. came 0.800 0.0335 Do Not Test

HA F1 A. came vs LA F1 A. ahli 35.417 1.557 Do Not Test

HA F1 A. came vs LA F1 A. ahli 31.929 1.456 Do Not Test

HA F1 A. came vs HA F0 A. exig 30.500 1.341 Do Not Test

HA F1 A. came vs LA F1 A. ahli 29.333 1.289 Do Not Test

HA F1 A. came vs LA F0 A. sple 27.300 1.144 Do Not Test

HA F1 A. came vs HA F0 A. exig 25.100 1.052 Do Not Test

HA F1 A. came vs LA F0 A. sple 23.700 0.993 Do Not Test

HA F1 A. came vs LA F0 A. sple 20.600 0.863 Do Not Test

HA F1 A. came vs LA F1 A. sple 13.333 0.586 Do Not Test

HA F1 A. came vs HA F1 A. came 10.917 0.480 Do Not Test

HA F1 A. came vs HA F1 A. exig 3.500 0.147 Do Not Test

HA F1 A. came vs HA F0 A. came 1.600 0.0671 Do Not Test

HA F1 A. came vs HA F0 A. exig 0.250 0.0110 Do Not Test

HA F0 A. exig vs LA F1 A. ahli 35.167 1.546 Do Not Test

HA F0 A. exig vs LA F1 A. ahli 31.679 1.445 Do Not Test

HA F0 A. exig vs HA F0 A. exig 30.250 1.330 Do Not Test

HA F0 A. exig vs LA F1 A. ahli 29.083 1.278 Do Not Test

HA F0 A. exig vs LA F0 A. sple 27.050 1.134 Do Not Test

HA F0 A. exig vs HA F0 A. exig 24.850 1.041 Do Not Test

HA F0 A. exig vs LA F0 A. sple 23.450 0.983 Do Not Test

HA F0 A. exig vs LA F0 A. sple 20.350 0.853 Do Not Test

HA F0 A. exig vs LA F1 A. sple 13.083 0.575 Do Not Test

HA F0 A. exig vs HA F1 A. came 10.667 0.469 Do Not Test

HA F0 A. exig vs HA F1 A. exig 3.250 0.136 Do Not Test

HA F0 A. exig vs HA F0 A. came 1.350 0.0566 Do Not Test

HA F0 A. came vs LA F1 A. ahli 33.817 1.417 Do Not Test

HA F0 A. came vs LA F1 A. ahli 30.329 1.314 Do Not Test

HA F0 A. came vs HA F0 A. exig 28.900 1.211 Do Not Test

HA F0 A. came vs LA F1 A. ahli 27.733 1.162 Do Not Test

HA F0 A. came vs LA F0 A. sple 25.700 1.031 Do Not Test

HA F0 A. came vs HA F0 A. exig 23.500 0.943 Do Not Test

HA F0 A. came vs LA F0 A. sple 22.100 0.887 Do Not Test

HA F0 A. came vs LA F0 A. sple 19 0.762 Do Not Test

HA F0 A. came vs LA F1 A. sple 11.733 0.492 Do Not Test

HA F0 A. came vs HA F1 A. came 9.317 0.390 Do Not Test

HA F0 A. came vs HA F1 A. exig 1.900 0.0762 Do Not Test

HA F1 A. exig vs LA F1 A. ahli 31.917 1.338 Do Not Test

HA F1 A. exig vs LA F1 A. ahli 28.429 1.232 Do Not Test

HA F1 A. exig vs HA F0 A. exig 27.000 1.132 Do Not Test

HA F1 A. exig vs LA F1 A. ahli 25.833 1.083 Do Not Test

HA F1 A. exig vs LA F0 A. sple 23.800 0.955 Do Not Test

HA F1 A. exig vs HA F0 A. exig 21.600 0.867 Do Not Test

HA F1 A. exig vs LA F0 A. sple 20.200 0.811 Do Not Test

HA F1 A. exig vs LA F0 A. sple 17.100 0.686 Do Not Test

HA F1 A. exig vs LA F1 A. sple 9.833 0.412 Do Not Test

HA F1 A. exig vs HA F1 A. came 7.417 0.311 Do Not Test

HA F1 A. came vs LA F1 A. ahli 24.500 1.077 Do Not Test

HA F1 A. came vs LA F1 A. ahli 21.012 0.958 Do Not Test

HA F1 A. came vs HA F0 A. exig 19.583 0.861 Do Not Test

HA F1 A. came vs LA F1 A. ahli 18.417 0.810 Do Not Test

HA F1 A. came vs LA F0 A. sple 16.383 0.687 Do Not Test

HA F1 A. came vs HA F0 A. exig 14.183 0.594 Do Not Test

HA F1 A. came vs LA F0 A. sple 12.783 0.536 Do Not Test

HA F1 A. came vs LA F0 A. sple 9.683 0.406 Do Not Test

HA F1 A. came vs LA F1 A. sple 2.417 0.106 Do Not Test

LA F1 A. sple vs LA F1 A. ahli 22.083 0.971 Do Not Test

LA F1 A. sple vs LA F1 A. ahli 18.595 0.848 Do Not Test

LA F1 A. sple vs HA F0 A. exig 17.167 0.755 Do Not Test

LA F1 A. sple vs LA F1 A. ahli 16.000 0.703 Do Not Test

LA F1 A. sple vs LA F0 A. sple 13.967 0.585 Do Not Test

LA F1 A. sple vs HA F0 A. exig 11.767 0.493 Do Not Test

LA F1 A. sple vs LA F0 A. sple 10.367 0.434 Do Not Test

LA F1 A. sple vs LA F0 A. sple 7.267 0.305 Do Not Test

LA F0 A. sple vs LA F1 A. ahli 14.817 0.621 Do Not Test

LA F0 A. sple vs LA F1 A. ahli 11.329 0.491 Do Not Test

LA F0 A. sple vs HA F0 A. exig 9.900 0.415 Do Not Test

LA F0 A. sple vs LA F1 A. ahli 8.733 0.366 Do Not Test

LA F0 A. sple vs LA F0 A. sple 6.700 0.269 Do Not Test

LA F0 A. sple vs HA F0 A. exig 4.500 0.181 Do Not Test

LA F0 A. sple vs LA F0 A. sple 3.100 0.124 Do Not Test

LA F0 A. sple vs LA F1 A. ahli 11.717 0.491 Do Not Test

LA F0 A. sple vs LA F1 A. ahli 8.229 0.357 Do Not Test

LA F0 A. sple vs HA F0 A. exig 6.800 0.285 Do Not Test

LA F0 A. sple vs LA F1 A. ahli 5.633 0.236 Do Not Test

LA F0 A. sple vs LA F0 A. sple 3.600 0.144 Do Not Test

LA F0 A. sple vs HA F0 A. exig 1.400 0.0562 Do Not Test

HA F0 A. exig vs LA F1 A. ahli 10.317 0.432 Do Not Test

HA F0 A. exig vs LA F1 A. ahli 6.829 0.296 Do Not Test

HA F0 A. exig vs HA F0 A. exig 5.400 0.226 Do Not Test

HA F0 A. exig vs LA F1 A. ahli 4.233 0.177 Do Not Test

HA F0 A. exig vs LA F0 A. sple 2.200 0.0883 Do Not Test

LA F0 A. sple vs LA F1 A. ahli 8.117 0.340 Do Not Test

LA F0 A. sple vs LA F1 A. ahli 4.629 0.201 Do Not Test

LA F0 A. sple vs HA F0 A. exig 3.200 0.134 Do Not Test

LA F0 A. sple vs LA F1 A. ahli 2.033 0.0852 Do Not Test

LA F1 A. ahli vs LA F1 A. ahli 6.083 0.267 Do Not Test

LA F1 A. ahli vs LA F1 A. ahli 2.595 0.118 Do Not Test

LA F1 A. ahli vs HA F0 A. exig 1.167 0.0513 Do Not Test

HA F0 A. exig vs LA F1 A. ahli 4.917 0.216 Do Not Test

HA F0 A. exig vs LA F1 A. ahli 1.429 0.0652 Do Not Test

LA F1 A. ahli vs LA F1 A. ahli 3.488 0.159 Do Not Test

Note: The multiple comparisons on ranks do not include an adjustment for ties.
