# Supplementary material for: Local Adaptation to Altitude Underlies Divergent Thermal Physiology in Tropical Killifishes of the Genus Aphyosemion
Source: PLoS One. 2013 Jan 22;8(1):e54345. doi: 10.1371/journal.pone.0054345 (PMC3551936; doi:10.1371/journal.pone.0054345)
Supplement: Text S2 — Two Way Analysis of Variance comparing routine metabolic rate at three temperatures among 2 altitudes×2 species×2 generations. (DOC) [file pone.0054345.s002.doc]

**Supporting Information 2**

**Two Way Analysis of Variance. comparing routine metabolic rate at three temperatures among 2 altitudes x 2 species x 2 generations**

General Linear Model

Dependent Variable: Log10(RMR) in μg O_2_ h^-1^

**Normality Test:** Passed (P = 0.104)

**Equal Variance Test:** Passed (P = 0.081)

**Source of Variation DF SS MS F P**

altitude/generation/species 7 0.268 0.0384 2.152 0.044

temperature 2 1.581 0.790 44.340 <0.001

altitude/gene x temperature 14 0.209 0.0149 0.838 0.627

Residual 111 1.978 0.0178

Total 134 4.058 0.0303

The difference in the mean values among the different levels of altitude/generation/species is greater than would be expected by chance after allowing for effects of differences in temperature . There is a statistically significant difference (P = 0.044). To isolate which group(s) differ from the others use a multiple comparison procedure.

The difference in the mean values among the different levels of temperature is greater than would be expected by chance after allowing for effects of differences in altitude/generation/species. There is a statistically significant difference (P = <0.001). To isolate which group(s) differ from the others use a multiple comparison procedure.

The effect of different levels of altitude/generation/species does not depend on what level of temperature is present. There is not a statistically significant interaction between altitude/generation/species and temperature . (P = 0.627)

Power of performed test with alpha = 0.0500: for altitude/generation/species : 0.471

Power of performed test with alpha = 0.0500: for temperature : 1.000

Power of performed test with alpha = 0.0500: for altitude/gene x temperature : 0.0500

Least square means for altitude/generation/species :

**Group Mean SEM**

HA F0 A. exiguum 2.042 0.0325

HA F1 A. exiguum 1.909 0.0340

HA F0 A. cameronense 2.022 0.0345

HA F1 A. cameronense 1.970 0.0315

LA F0 A. ahli 2.005 0.0325

LA F1 A. ahli 1.933 0.0307

LA F0 A. splendopleure 1.921 0.0345

LA F1 A. splendopleure 1.962 0.0315

Least square means for temperature :

**Group Mean SEM**

19 1.837 0.0197

25 1.977 0.0208

28 2.098 0.0196

Least square means for altitude/gene x temperature :

**Group Mean SEM**

HA F0 A. exiguum x 19 1.888 0.0545

HA F0 A. exiguum x 25 2.112 0.0597

HA F0 A. exiguum x 28 2.126 0.0545

HA F1 A. exiguum x 19 1.687 0.0545

HA F1 A. exiguum x 25 1.966 0.0668

HA F1 A. exiguum x 28 2.075 0.0545

HA F0 A. cameronense x 19 1.917 0.0597

HA F0 A. cameronense x 25 2.015 0.0597

HA F0 A. cameronense x 28 2.133 0.0597

HA F1 A. cameronense x 19 1.858 0.0545

HA F1 A. cameronense x 25 1.982 0.0545

HA F1 A. cameronense x 28 2.071 0.0545

LA F0 A. ahli x 19 1.934 0.0545

LA F0 A. ahli x 25 1.998 0.0597

LA F0 A. ahli x 28 2.083 0.0545

LA F1 A. ahli x 19 1.822 0.0545

LA F1 A. ahli x 25 1.898 0.0545

LA F1 A. ahli x 28 2.080 0.0505

LA F0 A. sple x 19 1.786 0.0597

LA F0 A. sple x 25 1.911 0.0597

LA F0 A. sple x 28 2.065 0.0597

LA F1 A. sple x 19 1.800 0.0545

LA F1 A. sple x 25 1.931 0.0545

LA F1 A. sple x 28 2.154 0.0545

All Pairwise Multiple Comparison Procedures (Holm-Sidak method):

Overall significance level = 0.05

Comparisons for factor: **altitude/generation/species**

**Comparison Diff of Means t Unadjusted P Critical Level Significant?**

HA F0 A. exi vs. HA F1 A. exi 0.133 2.826 0.006 0.002 No

HA F0 A. exi vs. LA F0 A. spl 0.121 2.559 0.012 0.002 No

HA F0 A. exi vs. LA F1 A. ahl 0.109 2.435 0.017 0.002 No

HA F0 A. cam vs. HA F1 A. exi 0.112 2.324 0.022 0.002 No

HA F0 A. cam vs. LA F0 A. spl 0.101 2.068 0.041 0.002 No

LA F0 A. ahl vs. HA F1 A. exi 0.0958 2.037 0.044 0.002 No

HA F0 A. cam vs. LA F1 A. ahl 0.0884 1.916 0.058 0.002 No

HA F0 A. exi vs. LA F1 A. spl 0.0804 1.778 0.078 0.002 No

LA F0 A. ahl vs. LA F0 A. spl 0.0841 1.776 0.079 0.003 No

LA F0 A. ahl vs. LA F1 A. ahl 0.0717 1.604 0.111 0.003 No

HA F0 A. exi vs. HA F1 A. cam 0.0716 1.583 0.116 0.003 No

HA F1 A. cam vs. HA F1 A. exi 0.0613 1.323 0.188 0.003 No

HA F0 A. cam vs. LA F1 A. spl 0.0600 1.286 0.201 0.003 No

LA F1 A. spl vs. HA F1 A. exi 0.0525 1.133 0.260 0.003 No

HA F0 A. cam vs. HA F1 A. cam 0.0512 1.097 0.275 0.004 No

HA F1 A. cam vs. LA F0 A. spl 0.0496 1.064 0.290 0.004 No

LA F0 A. ahl vs. LA F1 A. spl 0.0433 0.958 0.340 0.004 No

LA F1 A. spl vs. LA F0 A. spl 0.0408 0.874 0.384 0.005 No

HA F1 A. cam vs. LA F1 A. ahl 0.0372 0.847 0.399 0.005 No

HA F0 A. exi vs. LA F0 A. ahl 0.0371 0.808 0.421 0.006 No

LA F0 A. ahl vs. HA F1 A. cam 0.0345 0.762 0.447 0.006 No

LA F1 A. spl vs. LA F1 A. ahl 0.0284 0.646 0.519 0.007 No

LA F1 A. ahl vs. HA F1 A. exi 0.0240 0.525 0.601 0.009 No

HA F0 A. exi vs. HA F0 A. cam 0.0204 0.431 0.667 0.010 No

HA F0 A. cam vs. LA F0 A. ahl 0.0167 0.353 0.725 0.013 No

LA F1 A. ahl vs. LA F0 A. spl 0.0124 0.268 0.789 0.017 No

LA F0 A. spl vs. HA F1 A. exi 0.0117 0.241 0.810 0.025 No

HA F1 A. cam vs. LA F1 A. spl 0.00883 0.198 0.843 0.050 No

Comparisons for factor: **temperature**

**Comparison Diff of Means t Unadjusted P Critical Level Significant?**

28 vs. 19 0.262 9.412 <0.001 0.017 Yes

25 vs. 19 0.140 4.882 <0.001 0.025 Yes

28 vs. 25 0.122 4.265 <0.001 0.050 Yes

Comparisons for factor: **temperature within HA F0 A. exiguum**

**Comparison Diff of Means t Unadjusted P Critical Level**

28 vs. 19 0.237 3.080 0.003 0.017

25 vs. 19 0.224 2.775 0.006 0.025

28 vs. 25 0.0131 0.162 0.871 0.050

**Comparison Significant?**

28 vs. 19 Yes

25 vs. 19 Yes

28 vs. 25 No

Comparisons for factor: **temperature within HA F1 A. exiguum**

**Comparison Diff of Means t Unadjusted P Critical Level**

28 vs. 19 0.388 5.037 <0.001 0.017

25 vs. 19 0.279 3.235 0.002 0.025

28 vs. 25 0.109 1.270 0.207 0.050

**Comparison Significant?**

28 vs. 19 Yes

25 vs. 19 Yes

28 vs. 25 No

Comparisons for factor: **temperature within HA F0 A. cameronense**

**Comparison Diff of Means t Unadjusted P Critical Level**

28 vs. 19 0.215 2.551 0.012 0.017

28 vs. 25 0.118 1.393 0.166 0.025

25 vs. 19 0.0978 1.158 0.249 0.050

**Comparison Significant?**

28 vs. 19 Yes

28 vs. 25 No

25 vs. 19 No

Comparisons for factor: **temperature within HA F1 A. cameronense**

**Comparison Diff of Means t Unadjusted P Critical Level**

28 vs. 19 0.213 2.761 0.007 0.017

25 vs. 19 0.123 1.599 0.113 0.025

28 vs. 25 0.0895 1.161 0.248 0.050

**Comparison Significant?**

28 vs. 19 Yes

25 vs. 19 No

28 vs. 25 No

Comparisons for factor: **temperature within LA F0 A. ahli**

**Comparison Diff of Means t Unadjusted P Critical Level**

28 vs. 19 0.149 1.932 0.056 0.017

28 vs. 25 0.0854 1.056 0.293 0.025

25 vs. 19 0.0635 0.786 0.434 0.050

**Comparison Significant?**

28 vs. 19 No

28 vs. 25 No

25 vs. 19 No

Comparisons for factor: **temperature within LA F1 A. ahli**

**Comparison Diff of Means t Unadjusted P Critical Level**

28 vs. 19 0.258 3.469 <0.001 0.017

28 vs. 25 0.182 2.455 0.016 0.025

25 vs. 19 0.0753 0.977 0.330 0.050

**Comparison Significant?**

28 vs. 19 Yes

28 vs. 25 Yes

25 vs. 19 No

Comparisons for factor: **temperature within LA F0 A. splendopleure**

**Comparison Diff of Means t Unadjusted P Critical Level**

28 vs. 19 0.279 3.308 0.001 0.017

28 vs. 25 0.154 1.823 0.071 0.025

25 vs. 19 0.125 1.485 0.140 0.050

**Comparison Significant?**

28 vs. 19 Yes

28 vs. 25 No

25 vs. 19 No

Comparisons for factor: **temperature within LA F1 A. splendopleure**

**Comparison Diff of Means t Unadjusted P Critical Level**

28 vs. 19 0.354 4.590 <0.001 0.017

28 vs. 25 0.223 2.889 0.005 0.025

25 vs. 19 0.131 1.701 0.092 0.050

**Comparison Significant?**

28 vs. 19 Yes

28 vs. 25 Yes

25 vs. 19 No

Comparisons for factor: **altitude/generation/species within 19**

**Comparison Diff of Means t Unadjusted P Critical Level**

LA F0 A. ahl vs. HA F1 A. exi 0.247 3.208 0.002 0.002

HA F0 A. cam vs. HA F1 A. exi 0.230 2.850 0.005 0.002

HA F0 A. exi vs. HA F1 A. exi 0.201 2.612 0.010 0.002

HA F1 A. cam vs. HA F1 A. exi 0.172 2.226 0.028 0.002

LA F0 A. ahl vs. LA F0 A. spl 0.148 1.833 0.069 0.002

LA F1 A. ahl vs. HA F1 A. exi 0.135 1.756 0.082 0.002

LA F0 A. ahl vs. LA F1 A. spl 0.134 1.741 0.085 0.002

HA F0 A. cam vs. LA F0 A. spl 0.131 1.555 0.123 0.002

LA F1 A. spl vs. HA F1 A. exi 0.113 1.468 0.145 0.003

LA F0 A. ahl vs. LA F1 A. ahl 0.112 1.452 0.149 0.003

HA F0 A. cam vs. LA F1 A. spl 0.117 1.451 0.150 0.003

HA F0 A. exi vs. LA F0 A. spl 0.102 1.265 0.209 0.003

LA F0 A. spl vs. HA F1 A. exi 0.0991 1.226 0.223 0.003

HA F0 A. cam vs. LA F1 A. ahl 0.0950 1.176 0.242 0.003

HA F0 A. exi vs. LA F1 A. spl 0.0882 1.144 0.255 0.004

LA F0 A. ahl vs. HA F1 A. cam 0.0757 0.982 0.328 0.004

HA F1 A. cam vs. LA F0 A. spl 0.0725 0.897 0.372 0.004

HA F0 A. exi vs. LA F1 A. ahl 0.0660 0.856 0.394 0.005

HA F1 A. cam vs. LA F1 A. spl 0.0585 0.758 0.450 0.005

HA F0 A. cam vs. HA F1 A. cam 0.0588 0.727 0.468 0.006

LA F0 A. ahl vs. HA F0 A. exi 0.0460 0.597 0.552 0.006

HA F1 A. cam vs. LA F1 A. ahl 0.0362 0.470 0.639 0.007

LA F1 A. ahl vs. LA F0 A. spl 0.0363 0.449 0.655 0.009

HA F0 A. exi vs. HA F1 A. cam 0.0297 0.386 0.701 0.010

HA F0 A. cam vs. HA F0 A. exi 0.0291 0.360 0.720 0.013

LA F1 A. ahl vs. LA F1 A. spl 0.0222 0.288 0.774 0.017

LA F0 A. ahl vs. HA F0 A. cam 0.0169 0.209 0.835 0.025

LA F1 A. spl vs. LA F0 A. spl 0.0140 0.174 0.862 0.050

**Comparison Significant?**

LA F0 A. ahl vs. HA F1 A. exi Yes

HA F0 A. cam vs. HA F1 A. exi No

HA F0 A. exi vs. HA F1 A. exi No

HA F1 A. cam vs. HA F1 A. exi No

LA F0 A. ahl vs. LA F0 A. spl No

LA F1 A. ahl vs. HA F1 A. exi No

LA F0 A. ahl vs. LA F1 A. spl No

HA F0 A. cam vs. LA F0 A. spl No

LA F1 A. spl vs. HA F1 A. exi No

LA F0 A. ahl vs. LA F1 A. ahl No

HA F0 A. cam vs. LA F1 A. spl No

HA F0 A. exi vs. LA F0 A. spl No

LA F0 A. spl vs. HA F1 A. exi No

HA F0 A. cam vs. LA F1 A. ahl No

HA F0 A. exi vs. LA F1 A. spl No

LA F0 A. ahl vs. HA F1 A. cam No

HA F1 A. cam vs. LA F0 A. spl No

HA F0 A. exi vs. LA F1 A. ahl No

HA F1 A. cam vs. LA F1 A. spl No

HA F0 A. cam vs. HA F1 A. cam No

LA F0 A. ahl vs. HA F0 A. exi No

HA F1 A. cam vs. LA F1 A. ahl No

LA F1 A. ahl vs. LA F0 A. spl No

HA F0 A. exi vs. HA F1 A. cam No

HA F0 A. cam vs. HA F0 A. exi No

LA F1 A. ahl vs. LA F1 A. spl No

LA F0 A. ahl vs. HA F0 A. cam No

LA F1 A. spl vs. LA F0 A. spl No

Comparisons for factor: **altitude/generation/species within 25**

**Comparison Diff of Means t Unadjusted P Critical Level**

HA F0 A. exi vs. LA F1 A. ahl 0.215 2.659 0.009 0.002

HA F0 A. exi vs. LA F0 A. spl 0.201 2.383 0.019 0.002

HA F0 A. exi vs. LA F1 A. spl 0.181 2.243 0.027 0.002

HA F0 A. exi vs. HA F1 A. exi 0.147 1.640 0.104 0.002

HA F0 A. exi vs. HA F1 A. cam 0.131 1.618 0.109 0.002

HA F0 A. cam vs. LA F1 A. ahl 0.117 1.453 0.149 0.002

HA F0 A. exi vs. LA F0 A. ahl 0.115 1.360 0.177 0.002

LA F0 A. ahl vs. LA F1 A. ahl 0.100 1.238 0.218 0.002

HA F0 A. cam vs. LA F0 A. spl 0.104 1.229 0.222 0.003

HA F0 A. exi vs. HA F0 A. cam 0.0974 1.154 0.251 0.003

HA F1 A. cam vs. LA F1 A. ahl 0.0842 1.092 0.277 0.003

HA F0 A. cam vs. LA F1 A. spl 0.0839 1.038 0.302 0.003

LA F0 A. ahl vs. LA F0 A. spl 0.0864 1.023 0.309 0.003

HA F1 A. cam vs. LA F0 A. spl 0.0704 0.871 0.386 0.003

LA F0 A. ahl vs. LA F1 A. spl 0.0665 0.823 0.412 0.004

HA F1 A. exi vs. LA F1 A. ahl 0.0680 0.790 0.431 0.004

HA F1 A. cam vs. LA F1 A. spl 0.0506 0.656 0.513 0.004

HA F1 A. exi vs. LA F0 A. spl 0.0543 0.606 0.546 0.005

HA F0 A. cam vs. HA F1 A. exi 0.0494 0.552 0.582 0.005

LA F1 A. spl vs. LA F1 A. ahl 0.0336 0.436 0.664 0.006

HA F0 A. cam vs. HA F1 A. cam 0.0333 0.412 0.681 0.006

HA F1 A. exi vs. LA F1 A. spl 0.0345 0.400 0.690 0.007

LA F0 A. ahl vs. HA F1 A. exi 0.0321 0.358 0.721 0.009

LA F1 A. spl vs. LA F0 A. spl 0.0198 0.245 0.807 0.010

HA F0 A. cam vs. LA F0 A. ahl 0.0174 0.206 0.837 0.013

LA F0 A. ahl vs. HA F1 A. cam 0.0159 0.197 0.844 0.017

HA F1 A. cam vs. HA F1 A. exi 0.0161 0.187 0.852 0.025

LA F0 A. spl vs. LA F1 A. ahl 0.0137 0.170 0.865 0.050

**Comparison Significant?**

HA F0 A. exi vs. LA F1 A. ahl No

HA F0 A. exi vs. LA F0 A. spl No

HA F0 A. exi vs. LA F1 A. spl No

HA F0 A. exi vs. HA F1 A. exi No

HA F0 A. exi vs. HA F1 A. cam No

HA F0 A. cam vs. LA F1 A. ahl No

HA F0 A. exi vs. LA F0 A. ahl No

LA F0 A. ahl vs. LA F1 A. ahl No

HA F0 A. cam vs. LA F0 A. spl No

HA F0 A. exi vs. HA F0 A. cam No

HA F1 A. cam vs. LA F1 A. ahl No

HA F0 A. cam vs. LA F1 A. spl No

LA F0 A. ahl vs. LA F0 A. spl No

HA F1 A. cam vs. LA F0 A. spl No

LA F0 A. ahl vs. LA F1 A. spl No

HA F1 A. exi vs. LA F1 A. ahl No

HA F1 A. cam vs. LA F1 A. spl No

HA F1 A. exi vs. LA F0 A. spl No

HA F0 A. cam vs. HA F1 A. exi No

LA F1 A. spl vs. LA F1 A. ahl No

HA F0 A. cam vs. HA F1 A. cam No

HA F1 A. exi vs. LA F1 A. spl No

LA F0 A. ahl vs. HA F1 A. exi No

LA F1 A. spl vs. LA F0 A. spl No

HA F0 A. cam vs. LA F0 A. ahl No

LA F0 A. ahl vs. HA F1 A. cam No

HA F1 A. cam vs. HA F1 A. exi No

LA F0 A. spl vs. LA F1 A. ahl No

Comparisons for factor: **altitude/generation/species within 28**

**Comparison Diff of Means t Unadjusted P Critical Level**

LA F1 A. spl vs. LA F0 A. spl 0.0885 1.095 0.276 0.002

LA F1 A. spl vs. HA F1 A. cam 0.0826 1.071 0.286 0.002

LA F1 A. spl vs. HA F1 A. exi 0.0787 1.021 0.309 0.002

LA F1 A. spl vs. LA F1 A. ahl 0.0739 0.995 0.322 0.002

LA F1 A. spl vs. LA F0 A. ahl 0.0707 0.918 0.361 0.002

HA F0 A. cam vs. LA F0 A. spl 0.0674 0.799 0.426 0.002

HA F0 A. cam vs. HA F1 A. cam 0.0615 0.760 0.449 0.002

HA F0 A. exi vs. LA F0 A. spl 0.0603 0.746 0.457 0.002

HA F0 A. cam vs. HA F1 A. exi 0.0576 0.713 0.478 0.003

HA F0 A. exi vs. HA F1 A. cam 0.0544 0.705 0.482 0.003

HA F0 A. cam vs. LA F1 A. ahl 0.0528 0.675 0.501 0.003

HA F0 A. exi vs. HA F1 A. exi 0.0505 0.655 0.514 0.003

HA F0 A. exi vs. LA F1 A. ahl 0.0457 0.615 0.540 0.003

HA F0 A. cam vs. LA F0 A. ahl 0.0496 0.614 0.541 0.003

HA F0 A. exi vs. LA F0 A. ahl 0.0425 0.552 0.582 0.004

LA F1 A. spl vs. HA F0 A. exi 0.0282 0.366 0.715 0.004

LA F1 A. spl vs. HA F0 A. cam 0.0211 0.261 0.794 0.004

LA F0 A. ahl vs. LA F0 A. spl 0.0178 0.220 0.826 0.005

LA F1 A. ahl vs. LA F0 A. spl 0.0146 0.187 0.852 0.005

LA F0 A. ahl vs. HA F1 A. cam 0.0118 0.154 0.878 0.006

HA F1 A. exi vs. LA F0 A. spl 0.00983 0.122 0.903 0.006

LA F1 A. ahl vs. HA F1 A. cam 0.00866 0.117 0.907 0.007

LA F0 A. ahl vs. HA F1 A. exi 0.00798 0.104 0.918 0.009

HA F0 A. cam vs. HA F0 A. exi 0.00710 0.0878 0.930 0.010

HA F1 A. cam vs. LA F0 A. spl 0.00599 0.0740 0.941 0.013

LA F1 A. ahl vs. HA F1 A. exi 0.00481 0.0648 0.948 0.017

HA F1 A. exi vs. HA F1 A. cam 0.00385 0.0499 0.960 0.025

LA F0 A. ahl vs. LA F1 A. ahl 0.00317 0.0427 0.966 0.050

**Comparison Significant?**

LA F1 A. spl vs. LA F0 A. spl No

LA F1 A. spl vs. HA F1 A. cam No

LA F1 A. spl vs. HA F1 A. exi No

LA F1 A. spl vs. LA F1 A. ahl No

LA F1 A. spl vs. LA F0 A. ahl No

HA F0 A. cam vs. LA F0 A. spl No

HA F0 A. cam vs. HA F1 A. cam No

HA F0 A. exi vs. LA F0 A. spl No

HA F0 A. cam vs. HA F1 A. exi No

HA F0 A. exi vs. HA F1 A. cam No

HA F0 A. cam vs. LA F1 A. ahl No

HA F0 A. exi vs. HA F1 A. exi No

HA F0 A. exi vs. LA F1 A. ahl No

HA F0 A. cam vs. LA F0 A. ahl No

HA F0 A. exi vs. LA F0 A. ahl No

LA F1 A. spl vs. HA F0 A. exi No

LA F1 A. spl vs. HA F0 A. cam No

LA F0 A. ahl vs. LA F0 A. spl No

LA F1 A. ahl vs. LA F0 A. spl No

LA F0 A. ahl vs. HA F1 A. cam No

HA F1 A. exi vs. LA F0 A. spl No

LA F1 A. ahl vs. HA F1 A. cam No

LA F0 A. ahl vs. HA F1 A. exi No

HA F0 A. cam vs. HA F0 A. exi No

HA F1 A. cam vs. LA F0 A. spl No

LA F1 A. ahl vs. HA F1 A. exi No

HA F1 A. exi vs. HA F1 A. cam No

LA F0 A. ahl vs. LA F1 A. ahl No
