# Supplementary material for: Local Adaptation to Altitude Underlies Divergent Thermal Physiology in Tropical Killifishes of the Genus Aphyosemion
Source: PLoS One. 2013 Jan 22;8(1):e54345. doi: 10.1371/journal.pone.0054345 (PMC3551936; doi:10.1371/journal.pone.0054345)
Supplement: Text S3 — Two Way Analysis of Variance comparing peak SDA MO2 at three temperatures among 2 altitudes×2 species×2 generations. (DOC) [file pone.0054345.s003.doc]

**Supporting Information 3**

**Two Way Analysis of Variance comparing peak SDA MO_2_ at three temperatures among 2 altitudes x 2 species x 2 generations**

Dependent Variable: MO_2peak_ in μg O_2_ h^-1^

**Normality Test:** Passed (P = 0.322)

**Equal Variance Test:** Passed (P = 0.656)

**Source of Variation DF SS MS F P**

altitude/generation/species 7 76303.065 10900.438 1.726 0.110

temperature 2 77870.459 38935.229 6.164 0.003

altitude/gene x temperature 14 70365.749 5026.125 0.796 0.672

Residual 111 701128.874 6316.476

Total 134 932399.721 6958.207

The difference in the mean values among the different levels of altitude/generation/species is not great enough to exclude the possibility that the difference is just due to random sampling variability after allowing for the effects of differences in temperature . There is not a statistically significant difference (P = 0.110).

The difference in the mean values among the different levels of temperature is greater than would be expected by chance after allowing for effects of differences in altitude/generation/species. There is a statistically significant difference (P = 0.003). To isolate which group(s) differ from the others use a multiple comparison procedure.

The effect of different levels of altitude/generation/species does not depend on what level of temperature is present. There is not a statistically significant interaction between altitude/generation/species and temperature . (P = 0.672)

Power of performed test with alpha = 0.0500: for altitude/generation/species : 0.296

Power of performed test with alpha = 0.0500: for temperature : 0.827

Power of performed test with alpha = 0.0500: for altitude/gene x temperature : 0.0500

Least square means for altitude/generation/species :

**Group Mean SEM**

HA F0 A. exiguum 214.004 19.347

HA F1 A. exiguum 211.085 20.234

HA F0 A. cameronense 230.471 20.521

HA F1 A. cameronense 216.602 18.733

LA F0 A. ahli 198.274 19.347

LA F1 A. ahli 268.051 18.281

LA F0 A. splendopleure 219.004 20.521

LA F1 A. splendopleure 261.108 18.733

Least square means for temperature :

**Group Mean SEM**

19 197.282 11.755

25 257.153 12.368

28 227.540 11.654

Least square means for altitude/gene x temperature :

**Group Mean SEM**

HA F0 A. exiguum x 19 180.806 32.446

HA F0 A. exiguum x 25 239.396 35.543

HA F0 A. exiguum x 28 221.809 32.446

HA F1 A. exiguum x 19 211.967 32.446

HA F1 A. exiguum x 25 221.841 39.738

HA F1 A. exiguum x 28 199.447 32.446

HA F0 A. cameronense x 19 193.300 35.543

HA F0 A. cameronense x 25 250.712 35.543

HA F0 A. cameronense x 28 247.400 35.543

HA F1 A. cameronense x 19 163.409 32.446

HA F1 A. cameronense x 25 247.565 32.446

HA F1 A. cameronense x 28 238.833 32.446

LA F0 A. ahli x 19 179.088 32.446

LA F0 A. ahli x 25 276.777 35.543

LA F0 A. ahli x 28 138.957 32.446

LA F1 A. ahli x 19 223.711 32.446

LA F1 A. ahli x 25 321.295 32.446

LA F1 A. ahli x 28 259.148 30.039

LA F0 A. sple x 19 185.649 35.543

LA F0 A. sple x 25 247.464 35.543

LA F0 A. sple x 28 223.899 35.543

LA F1 A. sple x 19 240.323 32.446

LA F1 A. sple x 25 252.174 32.446

LA F1 A. sple x 28 290.826 32.446

All Pairwise Multiple Comparison Procedures (Holm-Sidak method):

Overall significance level = 0.05

Comparisons for factor: **altitude/generation/species**

**Comparison Diff of Means t Unadjusted P Critical Level**

LA F1 A. ahl vs. LA F0 A. ahl 69.777 2.621 0.010 0.002

LA F1 A. spl vs. LA F0 A. ahl 62.834 2.333 0.021 0.002

LA F1 A. ahl vs. HA F1 A. exi 56.966 2.089 0.039 0.002

LA F1 A. ahl vs. HA F0 A. exi 54.047 2.030 0.045 0.002

LA F1 A. ahl vs. HA F1 A. cam 51.449 1.966 0.052 0.002

LA F1 A. spl vs. HA F1 A. exi 50.023 1.814 0.072 0.002

LA F1 A. ahl vs. LA F0 A. spl 49.047 1.785 0.077 0.002

LA F1 A. spl vs. HA F0 A. exi 47.104 1.749 0.083 0.002

LA F1 A. spl vs. HA F1 A. cam 44.505 1.680 0.096 0.003

LA F1 A. spl vs. LA F0 A. spl 42.104 1.515 0.133 0.003

LA F1 A. ahl vs. HA F0 A. cam 37.581 1.367 0.174 0.003

HA F0 A. cam vs. LA F0 A. ahl 32.197 1.142 0.256 0.003

LA F1 A. spl vs. HA F0 A. cam 30.637 1.103 0.273 0.003

LA F0 A. spl vs. LA F0 A. ahl 20.730 0.735 0.464 0.003

HA F1 A. cam vs. LA F0 A. ahl 18.328 0.681 0.498 0.004

HA F0 A. cam vs. HA F1 A. exi 19.386 0.673 0.503 0.004

HA F0 A. cam vs. HA F0 A. exi 16.467 0.584 0.561 0.004

HA F0 A. exi vs. LA F0 A. ahl 15.730 0.575 0.567 0.005

HA F0 A. cam vs. HA F1 A. cam 13.868 0.499 0.619 0.005

HA F1 A. exi vs. LA F0 A. ahl 12.811 0.458 0.648 0.006

HA F0 A. cam vs. LA F0 A. spl 11.467 0.395 0.694 0.006

LA F0 A. spl vs. HA F1 A. exi 7.919 0.275 0.784 0.007

LA F1 A. ahl vs. LA F1 A. spl 6.944 0.265 0.791 0.009

HA F1 A. cam vs. HA F1 A. exi 5.517 0.200 0.842 0.010

LA F0 A. spl vs. HA F0 A. exi 5.000 0.177 0.860 0.013

HA F0 A. exi vs. HA F1 A. exi 2.919 0.104 0.917 0.017

HA F1 A. cam vs. HA F0 A. exi 2.598 0.0965 0.923 0.025

LA F0 A. spl vs. HA F1 A. cam 2.401 0.0864 0.931 0.050

**Comparison Significant?**

LA F1 A. ahl vs. LA F0 A. ahl No

LA F1 A. spl vs. LA F0 A. ahl No

LA F1 A. ahl vs. HA F1 A. exi No

LA F1 A. ahl vs. HA F0 A. exi No

LA F1 A. ahl vs. HA F1 A. cam No

LA F1 A. spl vs. HA F1 A. exi No

LA F1 A. ahl vs. LA F0 A. spl No

LA F1 A. spl vs. HA F0 A. exi No

LA F1 A. spl vs. HA F1 A. cam No

LA F1 A. spl vs. LA F0 A. spl No

LA F1 A. ahl vs. HA F0 A. cam No

HA F0 A. cam vs. LA F0 A. ahl No

LA F1 A. spl vs. HA F0 A. cam No

LA F0 A. spl vs. LA F0 A. ahl No

HA F1 A. cam vs. LA F0 A. ahl No

HA F0 A. cam vs. HA F1 A. exi No

HA F0 A. cam vs. HA F0 A. exi No

HA F0 A. exi vs. LA F0 A. ahl No

HA F0 A. cam vs. HA F1 A. cam No

HA F1 A. exi vs. LA F0 A. ahl No

HA F0 A. cam vs. LA F0 A. spl No

LA F0 A. spl vs. HA F1 A. exi No

LA F1 A. ahl vs. LA F1 A. spl No

HA F1 A. cam vs. HA F1 A. exi No

LA F0 A. spl vs. HA F0 A. exi No

HA F0 A. exi vs. HA F1 A. exi No

HA F1 A. cam vs. HA F0 A. exi No

LA F0 A. spl vs. HA F1 A. cam No

Comparisons for factor: **temperature**

**Comparison Diff of Means t Unadjusted P Critical Level Significant?**

25 vs. 19 59.871 3.509 <0.001 0.017 Yes

28 vs. 19 30.258 1.828 0.070 0.025 No

25 vs. 28 29.613 1.743 0.084 0.050 No

Comparisons for factor: **temperature within HA F0 A. exiguum**

**Comparison Diff of Means t Unadjusted P Critical Level**

25 vs. 19 58.590 1.217 0.226 0.017

28 vs. 19 41.003 0.894 0.373 0.025

25 vs. 28 17.587 0.365 0.715 0.050

**Comparison Significant?**

25 vs. 19 No

28 vs. 19 No

25 vs. 28 No

Comparisons for factor: **temperature within HA F1 A. exiguum**

**Comparison Diff of Means t Unadjusted P Critical Level**

25 vs. 28 22.394 0.437 0.663 0.017

19 vs. 28 12.520 0.273 0.785 0.025

25 vs. 19 9.874 0.192 0.848 0.050

**Comparison Significant?**

25 vs. 28 No

19 vs. 28 No

25 vs. 19 No

Comparisons for factor: **temperature within HA F0 A. cameronense**

**Comparison Diff of Means t Unadjusted P Critical Level**

25 vs. 19 57.412 1.142 0.256 0.017

28 vs. 19 54.100 1.076 0.284 0.025

25 vs. 28 3.312 0.0659 0.948 0.050

**Comparison Significant?**

25 vs. 19 No

28 vs. 19 No

25 vs. 28 No

Comparisons for factor: **temperature within HA F1 A. cameronense**

**Comparison Diff of Means t Unadjusted P Critical Level**

25 vs. 19 84.156 1.834 0.069 0.017

28 vs. 19 75.424 1.644 0.103 0.025

25 vs. 28 8.731 0.190 0.849 0.050

**Comparison Significant?**

25 vs. 19 No

28 vs. 19 No

25 vs. 28 No

Comparisons for factor: **temperature within LA F0 A. ahli**

**Comparison Diff of Means t Unadjusted P Critical Level**

25 vs. 28 137.820 2.864 0.005 0.017

25 vs. 19 97.689 2.030 0.045 0.025

19 vs. 28 40.131 0.875 0.384 0.050

**Comparison Significant?**

25 vs. 28 Yes

25 vs. 19 No

19 vs. 28 No

Comparisons for factor: **temperature within LA F1 A. ahli**

**Comparison Diff of Means t Unadjusted P Critical Level**

25 vs. 19 97.584 2.127 0.036 0.017

25 vs. 28 62.147 1.406 0.163 0.025

28 vs. 19 35.437 0.801 0.425 0.050

**Comparison Significant?**

25 vs. 19 No

25 vs. 28 No

28 vs. 19 No

Comparisons for factor: **temperature within LA F0 A. splendopleure**

**Comparison Diff of Means t Unadjusted P Critical Level**

25 vs. 19 61.815 1.230 0.221 0.017

28 vs. 19 38.250 0.761 0.448 0.025

25 vs. 28 23.565 0.469 0.640 0.050

**Comparison Significant?**

25 vs. 19 No

28 vs. 19 No

25 vs. 28 No

Comparisons for factor: **temperature within LA F1 A. splendopleure**

**Comparison Diff of Means t Unadjusted P Critical Level**

28 vs. 19 50.502 1.101 0.273 0.017

28 vs. 25 38.652 0.842 0.401 0.025

25 vs. 19 11.851 0.258 0.797 0.050

**Comparison Significant?**

28 vs. 19 No

28 vs. 25 No

25 vs. 19 No

Comparisons for factor: **altitude/generation/species within 19**

**Comparison Diff of Means t Unadjusted P Critical Level**

LA F1 A. spl vs. HA F1 A. cam 76.914 1.676 0.097 0.002

LA F1 A. spl vs. LA F0 A. ahl 61.235 1.335 0.185 0.002

LA F1 A. ahl vs. HA F1 A. cam 60.301 1.314 0.191 0.002

LA F1 A. spl vs. HA F0 A. exi 59.517 1.297 0.197 0.002

LA F1 A. spl vs. LA F0 A. spl 54.674 1.136 0.258 0.002

HA F1 A. exi vs. HA F1 A. cam 48.558 1.058 0.292 0.002

LA F1 A. spl vs. HA F0 A. cam 47.024 0.977 0.331 0.002

LA F1 A. ahl vs. LA F0 A. ahl 44.623 0.972 0.333 0.002

LA F1 A. ahl vs. HA F0 A. exi 42.904 0.935 0.352 0.003

LA F1 A. ahl vs. LA F0 A. spl 38.062 0.791 0.431 0.003

HA F1 A. exi vs. LA F0 A. ahl 32.879 0.717 0.475 0.003

HA F1 A. exi vs. HA F0 A. exi 31.160 0.679 0.498 0.003

LA F1 A. ahl vs. HA F0 A. cam 30.411 0.632 0.529 0.003

HA F0 A. cam vs. HA F1 A. cam 29.891 0.621 0.536 0.003

LA F1 A. spl vs. HA F1 A. exi 28.357 0.618 0.538 0.004

HA F1 A. exi vs. LA F0 A. spl 26.318 0.547 0.586 0.004

LA F0 A. spl vs. HA F1 A. cam 22.240 0.462 0.645 0.004

HA F1 A. exi vs. HA F0 A. cam 18.667 0.388 0.699 0.005

HA F0 A. exi vs. HA F1 A. cam 17.397 0.379 0.705 0.005

LA F1 A. spl vs. LA F1 A. ahl 16.613 0.362 0.718 0.006

LA F0 A. ahl vs. HA F1 A. cam 15.679 0.342 0.733 0.006

HA F0 A. cam vs. LA F0 A. ahl 14.212 0.295 0.768 0.007

HA F0 A. cam vs. HA F0 A. exi 12.493 0.260 0.796 0.009

LA F1 A. ahl vs. HA F1 A. exi 11.744 0.256 0.798 0.010

HA F0 A. cam vs. LA F0 A. spl 7.651 0.152 0.879 0.013

LA F0 A. spl vs. LA F0 A. ahl 6.561 0.136 0.892 0.017

LA F0 A. spl vs. HA F0 A. exi 4.842 0.101 0.920 0.025

HA F0 A. exi vs. LA F0 A. ahl 1.719 0.0375 0.970 0.050

**Comparison Significant?**

LA F1 A. spl vs. HA F1 A. cam No

LA F1 A. spl vs. LA F0 A. ahl No

LA F1 A. ahl vs. HA F1 A. cam No

LA F1 A. spl vs. HA F0 A. exi No

LA F1 A. spl vs. LA F0 A. spl No

HA F1 A. exi vs. HA F1 A. cam No

LA F1 A. spl vs. HA F0 A. cam No

LA F1 A. ahl vs. LA F0 A. ahl No

LA F1 A. ahl vs. HA F0 A. exi No

LA F1 A. ahl vs. LA F0 A. spl No

HA F1 A. exi vs. LA F0 A. ahl No

HA F1 A. exi vs. HA F0 A. exi No

LA F1 A. ahl vs. HA F0 A. cam No

HA F0 A. cam vs. HA F1 A. cam No

LA F1 A. spl vs. HA F1 A. exi No

HA F1 A. exi vs. LA F0 A. spl No

LA F0 A. spl vs. HA F1 A. cam No

HA F1 A. exi vs. HA F0 A. cam No

HA F0 A. exi vs. HA F1 A. cam No

LA F1 A. spl vs. LA F1 A. ahl No

LA F0 A. ahl vs. HA F1 A. cam No

HA F0 A. cam vs. LA F0 A. ahl No

HA F0 A. cam vs. HA F0 A. exi No

LA F1 A. ahl vs. HA F1 A. exi No

HA F0 A. cam vs. LA F0 A. spl No

LA F0 A. spl vs. LA F0 A. ahl No

LA F0 A. spl vs. HA F0 A. exi No

HA F0 A. exi vs. LA F0 A. ahl No

Comparisons for factor: **altitude/generation/species within 25**

**Comparison Diff of Means t Unadjusted P Critical Level**

LA F1 A. ahl vs. HA F1 A. exi 99.454 1.939 0.055 0.002

LA F1 A. ahl vs. HA F0 A. exi 81.899 1.702 0.092 0.002

LA F1 A. ahl vs. HA F1 A. cam 73.730 1.607 0.111 0.002

LA F1 A. ahl vs. LA F0 A. spl 73.831 1.534 0.128 0.002

LA F1 A. ahl vs. LA F1 A. spl 69.121 1.506 0.135 0.002

LA F1 A. ahl vs. HA F0 A. cam 70.583 1.467 0.145 0.002

LA F0 A. ahl vs. HA F1 A. exi 54.936 1.030 0.305 0.002

LA F1 A. ahl vs. LA F0 A. ahl 44.518 0.925 0.357 0.002

LA F0 A. ahl vs. HA F0 A. exi 37.381 0.744 0.459 0.003

LA F0 A. ahl vs. HA F1 A. cam 29.212 0.607 0.545 0.003

LA F1 A. spl vs. HA F1 A. exi 30.333 0.591 0.556 0.003

LA F0 A. ahl vs. LA F0 A. spl 29.313 0.583 0.561 0.003

HA F0 A. cam vs. HA F1 A. exi 28.871 0.542 0.589 0.003

LA F0 A. ahl vs. HA F0 A. cam 26.065 0.519 0.605 0.003

LA F0 A. ahl vs. LA F1 A. spl 24.603 0.511 0.610 0.004

HA F1 A. cam vs. HA F1 A. exi 25.724 0.501 0.617 0.004

LA F0 A. spl vs. HA F1 A. exi 25.623 0.481 0.632 0.004

HA F0 A. exi vs. HA F1 A. exi 17.555 0.329 0.743 0.005

LA F1 A. spl vs. HA F0 A. exi 12.778 0.266 0.791 0.005

HA F0 A. cam vs. HA F0 A. exi 11.316 0.225 0.822 0.006

HA F1 A. cam vs. HA F0 A. exi 8.169 0.170 0.866 0.006

LA F0 A. spl vs. HA F0 A. exi 8.068 0.161 0.873 0.007

LA F1 A. spl vs. HA F1 A. cam 4.609 0.100 0.920 0.009

LA F1 A. spl vs. LA F0 A. spl 4.710 0.0979 0.922 0.010

HA F0 A. cam vs. HA F1 A. cam 3.147 0.0654 0.948 0.013

HA F0 A. cam vs. LA F0 A. spl 3.248 0.0646 0.949 0.017

LA F1 A. spl vs. HA F0 A. cam 1.462 0.0304 0.976 0.025

HA F1 A. cam vs. LA F0 A. spl 0.101 0.00210 0.998 0.050

**Comparison Significant?**

LA F1 A. ahl vs. HA F1 A. exi No

LA F1 A. ahl vs. HA F0 A. exi No

LA F1 A. ahl vs. HA F1 A. cam No

LA F1 A. ahl vs. LA F0 A. spl No

LA F1 A. ahl vs. LA F1 A. spl No

LA F1 A. ahl vs. HA F0 A. cam No

LA F0 A. ahl vs. HA F1 A. exi No

LA F1 A. ahl vs. LA F0 A. ahl No

LA F0 A. ahl vs. HA F0 A. exi No

LA F0 A. ahl vs. HA F1 A. cam No

LA F1 A. spl vs. HA F1 A. exi No

LA F0 A. ahl vs. LA F0 A. spl No

HA F0 A. cam vs. HA F1 A. exi No

LA F0 A. ahl vs. HA F0 A. cam No

LA F0 A. ahl vs. LA F1 A. spl No

HA F1 A. cam vs. HA F1 A. exi No

LA F0 A. spl vs. HA F1 A. exi No

HA F0 A. exi vs. HA F1 A. exi No

LA F1 A. spl vs. HA F0 A. exi No

HA F0 A. cam vs. HA F0 A. exi No

HA F1 A. cam vs. HA F0 A. exi No

LA F0 A. spl vs. HA F0 A. exi No

LA F1 A. spl vs. HA F1 A. cam No

LA F1 A. spl vs. LA F0 A. spl No

HA F0 A. cam vs. HA F1 A. cam No

HA F0 A. cam vs. LA F0 A. spl No

LA F1 A. spl vs. HA F0 A. cam No

HA F1 A. cam vs. LA F0 A. spl No

Comparisons for factor: **altitude/generation/species within 28**

**Comparison Diff of Means t Unadjusted P Critical Level**

LA F1 A. spl vs. LA F0 A. ahl 151.869 3.310 0.001 0.002

LA F1 A. ahl vs. LA F0 A. ahl 120.191 2.718 0.008 0.002

HA F0 A. cam vs. LA F0 A. ahl 108.443 2.253 0.026 0.002

HA F1 A. cam vs. LA F0 A. ahl 99.876 2.177 0.032 0.002

LA F1 A. spl vs. HA F1 A. exi 91.379 1.991 0.049 0.002

HA F0 A. exi vs. LA F0 A. ahl 82.853 1.806 0.074 0.002

LA F0 A. spl vs. LA F0 A. ahl 84.942 1.765 0.080 0.002

LA F1 A. spl vs. HA F0 A. exi 69.016 1.504 0.135 0.002

LA F1 A. spl vs. LA F0 A. spl 66.927 1.391 0.167 0.003

LA F1 A. ahl vs. HA F1 A. exi 59.701 1.350 0.180 0.003

HA F1 A. exi vs. LA F0 A. ahl 60.490 1.318 0.190 0.003

LA F1 A. spl vs. HA F1 A. cam 51.992 1.133 0.260 0.003

HA F0 A. cam vs. HA F1 A. exi 47.953 0.996 0.321 0.003

LA F1 A. spl vs. HA F0 A. cam 43.426 0.902 0.369 0.003

HA F1 A. cam vs. HA F1 A. exi 39.386 0.858 0.393 0.004

LA F1 A. ahl vs. HA F0 A. exi 37.339 0.844 0.400 0.004

LA F1 A. ahl vs. LA F0 A. spl 35.249 0.757 0.450 0.004

LA F1 A. spl vs. LA F1 A. ahl 31.678 0.716 0.475 0.005

HA F0 A. cam vs. HA F0 A. exi 25.591 0.532 0.596 0.005

LA F0 A. spl vs. HA F1 A. exi 24.452 0.508 0.612 0.006

HA F0 A. exi vs. HA F1 A. exi 22.362 0.487 0.627 0.006

HA F0 A. cam vs. LA F0 A. spl 23.501 0.468 0.641 0.007

LA F1 A. ahl vs. HA F1 A. cam 20.315 0.459 0.647 0.009

HA F1 A. cam vs. HA F0 A. exi 17.024 0.371 0.711 0.010

HA F1 A. cam vs. LA F0 A. spl 14.935 0.310 0.757 0.013

LA F1 A. ahl vs. HA F0 A. cam 11.748 0.252 0.801 0.017

HA F0 A. cam vs. HA F1 A. cam 8.567 0.178 0.859 0.025

LA F0 A. spl vs. HA F0 A. exi 2.089 0.0434 0.965 0.050

**Comparison Significant?**

LA F1 A. spl vs. LA F0 A. ahl Yes

LA F1 A. ahl vs. LA F0 A. ahl No

HA F0 A. cam vs. LA F0 A. ahl No

HA F1 A. cam vs. LA F0 A. ahl No

LA F1 A. spl vs. HA F1 A. exi No

HA F0 A. exi vs. LA F0 A. ahl No

LA F0 A. spl vs. LA F0 A. ahl No

LA F1 A. spl vs. HA F0 A. exi No

LA F1 A. spl vs. LA F0 A. spl No

LA F1 A. ahl vs. HA F1 A. exi No

HA F1 A. exi vs. LA F0 A. ahl No

LA F1 A. spl vs. HA F1 A. cam No

HA F0 A. cam vs. HA F1 A. exi No

LA F1 A. spl vs. HA F0 A. cam No

HA F1 A. cam vs. HA F1 A. exi No

LA F1 A. ahl vs. HA F0 A. exi No

LA F1 A. ahl vs. LA F0 A. spl No

LA F1 A. spl vs. LA F1 A. ahl No

HA F0 A. cam vs. HA F0 A. exi No

LA F0 A. spl vs. HA F1 A. exi No

HA F0 A. exi vs. HA F1 A. exi No

HA F0 A. cam vs. LA F0 A. spl No

LA F1 A. ahl vs. HA F1 A. cam No

HA F1 A. cam vs. HA F0 A. exi No

HA F1 A. cam vs. LA F0 A. spl No

LA F1 A. ahl vs. HA F0 A. cam No

HA F0 A. cam vs. HA F1 A. cam No

LA F0 A. spl vs. HA F0 A. exi No
