# Supplementary material for: Local Adaptation to Altitude Underlies Divergent Thermal Physiology in Tropical Killifishes of the Genus Aphyosemion
Source: PLoS One. 2013 Jan 22;8(1):e54345. doi: 10.1371/journal.pone.0054345 (PMC3551936; doi:10.1371/journal.pone.0054345)
Supplement: Text S4 — Two Way Analysis of Variance comparing time to SDA peak at three temperatures among 2 altitudes×2 species×2 generations. (DOC) [file pone.0054345.s004.doc]

**Supporting Information 4**

**Two Way Analysis of Variance comparing time to SDA peak at three temperatures among 2 altitudes x 2 species x 2 generations**

General Linear Model

Dependent Variable: Log10(T_peak_) in minutes

**Normality Test:** Passed (P = 0.334)

**Equal Variance Test:** Passed (P = 0.062)

**Source of Variation DF SS MS F P**

altitude/generation/species 7 1.048 0.150 1.368 0.226

temperature 2 1.508 0.754 6.893 0.002

altitude/gene x temperature 14 4.736 0.338 3.093 <0.001

Residual 111 12.139 0.109

Total 134 19.472 0.145

Main effects cannot be properly interpreted if significant interaction is determined. This is because the size of a factor's effect depends upon the level of the other factor.

The effect of different levels of altitude/generation/species depends on what level of temperature is present. There is a statistically significant interaction between altitude/generation/species and temperature . (P = <0.001)

Power of performed test with alpha = 0.0500: for altitude/generation/species : 0.158

Power of performed test with alpha = 0.0500: for temperature : 0.880

Power of performed test with alpha = 0.0500: for altitude/gene x temperature : 0.946

Least square means for altitude/generation/species :

**Group Mean SEM**

HA F0 A. exiguum 2.075 0.0805

HA F1 A. exiguum 2.165 0.0842

HA F0 A. cameronense 2.016 0.0854

HA F1 A. cameronense 1.929 0.0779

LA F0 A. ahli 1.954 0.0805

LA F1 A. ahli 1.995 0.0761

LA F0 A. splendopleure 2.177 0.0854

LA F1 A. splendopleure 1.955 0.0779

Least square means for temperature :

**Group Mean SEM**

19 2.051 0.0489

25 1.894 0.0515

28 2.155 0.0485

Least square means for altitude/gene x temperature :

**Group Mean SEM**

HA F0 A. exiguum x 19 2.059 0.135

HA F0 A. exiguum x 25 2.012 0.148

HA F0 A. exiguum x 28 2.155 0.135

HA F1 A. exiguum x 19 1.928 0.135

HA F1 A. exiguum x 25 1.963 0.165

HA F1 A. exiguum x 28 2.604 0.135

HA F0 A. cameronense x 19 1.728 0.148

HA F0 A. cameronense x 25 1.988 0.148

HA F0 A. cameronense x 28 2.330 0.148

HA F1 A. cameronense x 19 1.714 0.135

HA F1 A. cameronense x 25 1.814 0.135

HA F1 A. cameronense x 28 2.259 0.135

LA F0 A. ahli x 19 2.068 0.135

LA F0 A. ahli x 25 1.753 0.148

LA F0 A. ahli x 28 2.040 0.135

LA F1 A. ahli x 19 2.242 0.135

LA F1 A. ahli x 25 1.967 0.135

LA F1 A. ahli x 28 1.778 0.125

LA F0 A. sple x 19 2.404 0.148

LA F0 A. sple x 25 1.979 0.148

LA F0 A. sple x 28 2.148 0.148

LA F1 A. sple x 19 2.264 0.135

LA F1 A. sple x 25 1.673 0.135

LA F1 A. sple x 28 1.928 0.135

All Pairwise Multiple Comparison Procedures (Holm-Sidak method):

Overall significance level = 0.05

Comparisons for factor: **altitude/generation/species**

**Comparison Diff of Means t Unadjusted P Critical Level**

LA F0 A. spl vs. HA F1 A. cam 0.248 2.146 0.034 0.002

HA F1 A. exi vs. HA F1 A. cam 0.236 2.056 0.042 0.002

LA F0 A. spl vs. LA F1 A. spl 0.222 1.921 0.057 0.002

LA F0 A. spl vs. LA F0 A. ahl 0.223 1.900 0.060 0.002

HA F1 A. exi vs. LA F1 A. spl 0.210 1.829 0.070 0.002

HA F1 A. exi vs. LA F0 A. ahl 0.211 1.810 0.073 0.002

LA F0 A. spl vs. LA F1 A. ahl 0.182 1.588 0.115 0.002

HA F1 A. exi vs. LA F1 A. ahl 0.169 1.493 0.138 0.002

LA F0 A. spl vs. HA F0 A. cam 0.161 1.337 0.184 0.003

HA F0 A. exi vs. HA F1 A. cam 0.147 1.308 0.194 0.003

HA F1 A. exi vs. HA F0 A. cam 0.149 1.245 0.216 0.003

HA F0 A. exi vs. LA F1 A. spl 0.120 1.075 0.285 0.003

HA F0 A. exi vs. LA F0 A. ahl 0.121 1.067 0.288 0.003

LA F0 A. spl vs. HA F0 A. exi 0.102 0.866 0.389 0.003

HA F1 A. exi vs. HA F0 A. exi 0.0894 0.768 0.444 0.004

HA F0 A. cam vs. HA F1 A. cam 0.0867 0.750 0.455 0.004

HA F0 A. exi vs. LA F1 A. ahl 0.0800 0.722 0.472 0.004

LA F1 A. ahl vs. HA F1 A. cam 0.0665 0.611 0.543 0.005

HA F0 A. cam vs. LA F0 A. ahl 0.0616 0.525 0.601 0.005

HA F0 A. cam vs. LA F1 A. spl 0.0606 0.524 0.601 0.006

HA F0 A. exi vs. HA F0 A. cam 0.0599 0.510 0.611 0.006

LA F1 A. ahl vs. LA F0 A. ahl 0.0414 0.374 0.709 0.007

LA F1 A. ahl vs. LA F1 A. spl 0.0405 0.372 0.711 0.009

LA F1 A. spl vs. HA F1 A. cam 0.0261 0.236 0.814 0.010

LA F0 A. ahl vs. HA F1 A. cam 0.0251 0.224 0.823 0.013

HA F0 A. cam vs. LA F1 A. ahl 0.0201 0.176 0.861 0.017

LA F0 A. spl vs. HA F1 A. exi 0.0122 0.101 0.919 0.025

LA F1 A. spl vs. LA F0 A. ahl 0.000981 0.00875 0.993 0.050

**Comparison Significant?**

LA F0 A. spl vs. HA F1 A. cam No

HA F1 A. exi vs. HA F1 A. cam No

LA F0 A. spl vs. LA F1 A. spl No

LA F0 A. spl vs. LA F0 A. ahl No

HA F1 A. exi vs. LA F1 A. spl No

HA F1 A. exi vs. LA F0 A. ahl No

LA F0 A. spl vs. LA F1 A. ahl No

HA F1 A. exi vs. LA F1 A. ahl No

LA F0 A. spl vs. HA F0 A. cam No

HA F0 A. exi vs. HA F1 A. cam No

HA F1 A. exi vs. HA F0 A. cam No

HA F0 A. exi vs. LA F1 A. spl No

HA F0 A. exi vs. LA F0 A. ahl No

LA F0 A. spl vs. HA F0 A. exi No

HA F1 A. exi vs. HA F0 A. exi No

HA F0 A. cam vs. HA F1 A. cam No

HA F0 A. exi vs. LA F1 A. ahl No

LA F1 A. ahl vs. HA F1 A. cam No

HA F0 A. cam vs. LA F0 A. ahl No

HA F0 A. cam vs. LA F1 A. spl No

HA F0 A. exi vs. HA F0 A. cam No

LA F1 A. ahl vs. LA F0 A. ahl No

LA F1 A. ahl vs. LA F1 A. spl No

LA F1 A. spl vs. HA F1 A. cam No

LA F0 A. ahl vs. HA F1 A. cam No

HA F0 A. cam vs. LA F1 A. ahl No

LA F0 A. spl vs. HA F1 A. exi No

LA F1 A. spl vs. LA F0 A. ahl No

Comparisons for factor: **temperature**

**Comparison Diff of Means t Unadjusted P Critical Level Significant?**

28 vs. 25 0.262 3.700 <0.001 0.017 Yes

19 vs. 25 0.157 2.213 0.029 0.025 No

28 vs. 19 0.105 1.518 0.132 0.050 No

Comparisons for factor: **temperature within HA F0 A. exiguum**

**Comparison Diff of Means t Unadjusted P Critical Level**

28 vs. 25 0.143 0.714 0.477 0.017

28 vs. 19 0.0955 0.500 0.618 0.025

19 vs. 25 0.0474 0.237 0.813 0.050

**Comparison Significant?**

28 vs. 25 No

28 vs. 19 No

19 vs. 25 No

Comparisons for factor: **temperature within HA F1 A. exiguum**

**Comparison Diff of Means t Unadjusted P Critical Level**

28 vs. 19 0.676 3.542 <0.001 0.017

28 vs. 25 0.641 3.001 0.003 0.025

25 vs. 19 0.0357 0.167 0.867 0.050

**Comparison Significant?**

28 vs. 19 Yes

28 vs. 25 Yes

25 vs. 19 No

Comparisons for factor: **temperature within HA F0 A. cameronense**

**Comparison Diff of Means t Unadjusted P Critical Level**

28 vs. 19 0.602 2.879 0.005 0.017

28 vs. 25 0.342 1.634 0.105 0.025

25 vs. 19 0.260 1.244 0.216 0.050

**Comparison Significant?**

28 vs. 19 Yes

28 vs. 25 No

25 vs. 19 No

Comparisons for factor: **temperature within HA F1 A. cameronense**

**Comparison Diff of Means t Unadjusted P Critical Level**

28 vs. 19 0.546 2.857 0.005 0.017

28 vs. 25 0.445 2.332 0.022 0.025

25 vs. 19 0.100 0.526 0.600 0.050

**Comparison Significant?**

28 vs. 19 Yes

28 vs. 25 Yes

25 vs. 19 No

Comparisons for factor: **temperature within LA F0 A. ahli**

**Comparison Diff of Means t Unadjusted P Critical Level**

19 vs. 25 0.315 1.574 0.118 0.017

28 vs. 25 0.287 1.435 0.154 0.025

19 vs. 28 0.0279 0.146 0.884 0.050

**Comparison Significant?**

19 vs. 25 No

28 vs. 25 No

19 vs. 28 No

Comparisons for factor: **temperature within LA F1 A. ahli**

**Comparison Diff of Means t Unadjusted P Critical Level**

19 vs. 28 0.464 2.519 0.013 0.017

19 vs. 25 0.275 1.441 0.152 0.025

25 vs. 28 0.188 1.024 0.308 0.050

**Comparison Significant?**

19 vs. 28 Yes

19 vs. 25 No

25 vs. 28 No

Comparisons for factor: **temperature within LA F0 A. splendopleure**

**Comparison Diff of Means t Unadjusted P Critical Level**

19 vs. 25 0.424 2.029 0.045 0.017

19 vs. 28 0.255 1.221 0.225 0.025

28 vs. 25 0.169 0.808 0.421 0.050

**Comparison Significant?**

19 vs. 25 No

19 vs. 28 No

28 vs. 25 No

Comparisons for factor: **temperature within LA F1 A. splendopleure**

**Comparison Diff of Means t Unadjusted P Critical Level**

19 vs. 25 0.591 3.095 0.002 0.017

19 vs. 28 0.336 1.761 0.081 0.025

28 vs. 25 0.255 1.334 0.185 0.050

**Comparison Significant?**

19 vs. 25 Yes

19 vs. 28 No

28 vs. 25 No

Comparisons for factor: **altitude/generation/species within 19**

**Comparison Diff of Means t Unadjusted P Critical Level**

LA F0 A. spl vs. HA F1 A. cam 0.690 3.446 <0.001 0.002

LA F0 A. spl vs. HA F0 A. cam 0.675 3.229 0.002 0.002

LA F1 A. spl vs. HA F1 A. cam 0.550 2.882 0.005 0.002

LA F1 A. ahl vs. HA F1 A. cam 0.528 2.766 0.007 0.002

LA F1 A. spl vs. HA F0 A. cam 0.536 2.676 0.009 0.002

LA F1 A. ahl vs. HA F0 A. cam 0.514 2.564 0.012 0.002

LA F0 A. spl vs. HA F1 A. exi 0.476 2.377 0.019 0.002

LA F0 A. ahl vs. HA F1 A. cam 0.355 1.858 0.066 0.002

HA F0 A. exi vs. HA F1 A. cam 0.346 1.811 0.073 0.003

LA F1 A. spl vs. HA F1 A. exi 0.336 1.762 0.081 0.003

LA F0 A. spl vs. HA F0 A. exi 0.344 1.719 0.088 0.003

LA F0 A. ahl vs. HA F0 A. cam 0.340 1.699 0.092 0.003

LA F0 A. spl vs. LA F0 A. ahl 0.335 1.674 0.097 0.003

HA F0 A. exi vs. HA F0 A. cam 0.331 1.654 0.101 0.003

LA F1 A. ahl vs. HA F1 A. exi 0.314 1.645 0.103 0.004

HA F1 A. exi vs. HA F1 A. cam 0.214 1.120 0.265 0.004

LA F1 A. spl vs. HA F0 A. exi 0.205 1.071 0.286 0.004

LA F1 A. spl vs. LA F0 A. ahl 0.196 1.025 0.308 0.005

HA F1 A. exi vs. HA F0 A. cam 0.199 0.996 0.322 0.005

LA F1 A. ahl vs. HA F0 A. exi 0.182 0.954 0.342 0.006

LA F1 A. ahl vs. LA F0 A. ahl 0.173 0.908 0.366 0.006

LA F0 A. spl vs. LA F1 A. ahl 0.162 0.809 0.420 0.007

LA F0 A. ahl vs. HA F1 A. exi 0.141 0.738 0.462 0.009

LA F0 A. spl vs. LA F1 A. spl 0.140 0.697 0.487 0.010

HA F0 A. exi vs. HA F1 A. exi 0.132 0.691 0.491 0.013

LA F1 A. spl vs. LA F1 A. ahl 0.0223 0.117 0.907 0.017

HA F0 A. cam vs. HA F1 A. cam 0.0145 0.0726 0.942 0.025

LA F0 A. ahl vs. HA F0 A. exi 0.00893 0.0468 0.963 0.050

**Comparison Significant?**

LA F0 A. spl vs. HA F1 A. cam Yes

LA F0 A. spl vs. HA F0 A. cam Yes

LA F1 A. spl vs. HA F1 A. cam No

LA F1 A. ahl vs. HA F1 A. cam No

LA F1 A. spl vs. HA F0 A. cam No

LA F1 A. ahl vs. HA F0 A. cam No

LA F0 A. spl vs. HA F1 A. exi No

LA F0 A. ahl vs. HA F1 A. cam No

HA F0 A. exi vs. HA F1 A. cam No

LA F1 A. spl vs. HA F1 A. exi No

LA F0 A. spl vs. HA F0 A. exi No

LA F0 A. ahl vs. HA F0 A. cam No

LA F0 A. spl vs. LA F0 A. ahl No

HA F0 A. exi vs. HA F0 A. cam No

LA F1 A. ahl vs. HA F1 A. exi No

HA F1 A. exi vs. HA F1 A. cam No

LA F1 A. spl vs. HA F0 A. exi No

LA F1 A. spl vs. LA F0 A. ahl No

HA F1 A. exi vs. HA F0 A. cam No

LA F1 A. ahl vs. HA F0 A. exi No

LA F1 A. ahl vs. LA F0 A. ahl No

LA F0 A. spl vs. LA F1 A. ahl No

LA F0 A. ahl vs. HA F1 A. exi No

LA F0 A. spl vs. LA F1 A. spl No

HA F0 A. exi vs. HA F1 A. exi No

LA F1 A. spl vs. LA F1 A. ahl No

HA F0 A. cam vs. HA F1 A. cam No

LA F0 A. ahl vs. HA F0 A. exi No

Comparisons for factor: **altitude/generation/species within 25**

**Comparison Diff of Means t Unadjusted P Critical Level**

HA F0 A. exi vs. LA F1 A. spl 0.339 1.692 0.093 0.002

HA F0 A. cam vs. LA F1 A. spl 0.315 1.574 0.118 0.002

LA F1 A. ahl vs. LA F1 A. spl 0.293 1.537 0.127 0.002

LA F0 A. spl vs. LA F1 A. spl 0.306 1.529 0.129 0.002

HA F1 A. exi vs. LA F1 A. spl 0.290 1.359 0.177 0.002

HA F0 A. exi vs. LA F0 A. ahl 0.259 1.238 0.218 0.002

HA F0 A. cam vs. LA F0 A. ahl 0.235 1.125 0.263 0.002

LA F0 A. spl vs. LA F0 A. ahl 0.226 1.081 0.282 0.002

LA F1 A. ahl vs. LA F0 A. ahl 0.213 1.066 0.289 0.003

HA F0 A. exi vs. HA F1 A. cam 0.198 0.989 0.325 0.003

HA F1 A. exi vs. LA F0 A. ahl 0.210 0.947 0.346 0.003

HA F0 A. cam vs. HA F1 A. cam 0.174 0.871 0.386 0.003

LA F0 A. spl vs. HA F1 A. cam 0.165 0.825 0.411 0.003

LA F1 A. ahl vs. HA F1 A. cam 0.153 0.799 0.426 0.003

HA F1 A. cam vs. LA F1 A. spl 0.141 0.738 0.462 0.004

HA F1 A. exi vs. HA F1 A. cam 0.149 0.699 0.486 0.004

LA F0 A. ahl vs. LA F1 A. spl 0.0800 0.400 0.690 0.004

HA F1 A. cam vs. LA F0 A. ahl 0.0608 0.304 0.762 0.005

HA F0 A. exi vs. LA F1 A. ahl 0.0455 0.227 0.821 0.005

HA F0 A. exi vs. HA F1 A. exi 0.0488 0.220 0.826 0.006

HA F0 A. exi vs. LA F0 A. spl 0.0328 0.157 0.876 0.006

HA F0 A. cam vs. HA F1 A. exi 0.0251 0.113 0.910 0.007

HA F0 A. exi vs. HA F0 A. cam 0.0236 0.113 0.910 0.009

HA F0 A. cam vs. LA F1 A. ahl 0.0218 0.109 0.913 0.010

LA F0 A. spl vs. HA F1 A. exi 0.0160 0.0721 0.943 0.013

LA F0 A. spl vs. LA F1 A. ahl 0.0127 0.0634 0.950 0.017

HA F0 A. cam vs. LA F0 A. spl 0.00915 0.0438 0.965 0.025

LA F1 A. ahl vs. HA F1 A. exi 0.00330 0.0155 0.988 0.050

**Comparison Significant?**

HA F0 A. exi vs. LA F1 A. spl No

HA F0 A. cam vs. LA F1 A. spl No

LA F1 A. ahl vs. LA F1 A. spl No

LA F0 A. spl vs. LA F1 A. spl No

HA F1 A. exi vs. LA F1 A. spl No

HA F0 A. exi vs. LA F0 A. ahl No

HA F0 A. cam vs. LA F0 A. ahl No

LA F0 A. spl vs. LA F0 A. ahl No

LA F1 A. ahl vs. LA F0 A. ahl No

HA F0 A. exi vs. HA F1 A. cam No

HA F1 A. exi vs. LA F0 A. ahl No

HA F0 A. cam vs. HA F1 A. cam No

LA F0 A. spl vs. HA F1 A. cam No

LA F1 A. ahl vs. HA F1 A. cam No

HA F1 A. cam vs. LA F1 A. spl No

HA F1 A. exi vs. HA F1 A. cam No

LA F0 A. ahl vs. LA F1 A. spl No

HA F1 A. cam vs. LA F0 A. ahl No

HA F0 A. exi vs. LA F1 A. ahl No

HA F0 A. exi vs. HA F1 A. exi No

HA F0 A. exi vs. LA F0 A. spl No

HA F0 A. cam vs. HA F1 A. exi No

HA F0 A. exi vs. HA F0 A. cam No

HA F0 A. cam vs. LA F1 A. ahl No

LA F0 A. spl vs. HA F1 A. exi No

LA F0 A. spl vs. LA F1 A. ahl No

HA F0 A. cam vs. LA F0 A. spl No

LA F1 A. ahl vs. HA F1 A. exi No

Comparisons for factor: **altitude/generation/species within 28**

**Comparison Diff of Means t Unadjusted P Critical Level**

HA F1 A. exi vs. LA F1 A. ahl 0.826 4.488 <0.001 0.002

HA F1 A. exi vs. LA F1 A. spl 0.676 3.541 <0.001 0.002

HA F1 A. exi vs. LA F0 A. ahl 0.563 2.951 0.004 0.002

HA F0 A. cam vs. LA F1 A. ahl 0.552 2.851 0.005 0.002

HA F1 A. cam vs. LA F1 A. ahl 0.481 2.614 0.010 0.002

HA F1 A. exi vs. HA F0 A. exi 0.449 2.351 0.020 0.002

HA F1 A. exi vs. LA F0 A. spl 0.456 2.275 0.025 0.002

HA F0 A. exi vs. LA F1 A. ahl 0.377 2.048 0.043 0.002

HA F0 A. cam vs. LA F1 A. spl 0.402 2.009 0.047 0.003

LA F0 A. spl vs. LA F1 A. ahl 0.370 1.911 0.059 0.003

HA F1 A. exi vs. HA F1 A. cam 0.345 1.805 0.074 0.003

HA F1 A. cam vs. LA F1 A. spl 0.331 1.735 0.085 0.003

HA F0 A. cam vs. LA F0 A. ahl 0.290 1.447 0.151 0.003

LA F0 A. ahl vs. LA F1 A. ahl 0.262 1.426 0.157 0.003

HA F1 A. exi vs. HA F0 A. cam 0.274 1.366 0.175 0.004

HA F0 A. exi vs. LA F1 A. spl 0.227 1.189 0.237 0.004

HA F1 A. cam vs. LA F0 A. ahl 0.219 1.145 0.255 0.004

LA F0 A. spl vs. LA F1 A. spl 0.220 1.101 0.273 0.005

HA F0 A. cam vs. HA F0 A. exi 0.175 0.875 0.383 0.005

HA F0 A. cam vs. LA F0 A. spl 0.182 0.870 0.386 0.006

LA F1 A. spl vs. LA F1 A. ahl 0.150 0.814 0.418 0.006

HA F0 A. exi vs. LA F0 A. ahl 0.114 0.599 0.550 0.007

LA F0 A. ahl vs. LA F1 A. spl 0.113 0.590 0.556 0.009

HA F1 A. cam vs. LA F0 A. spl 0.111 0.554 0.581 0.010

HA F1 A. cam vs. HA F0 A. exi 0.104 0.546 0.586 0.013

LA F0 A. spl vs. LA F0 A. ahl 0.108 0.538 0.592 0.017

HA F0 A. cam vs. HA F1 A. cam 0.0711 0.355 0.723 0.025

HA F0 A. exi vs. LA F0 A. spl 0.00668 0.0334 0.973 0.050

**Comparison Significant?**

HA F1 A. exi vs. LA F1 A. ahl Yes

HA F1 A. exi vs. LA F1 A. spl Yes

HA F1 A. exi vs. LA F0 A. ahl No

HA F0 A. cam vs. LA F1 A. ahl No

HA F1 A. cam vs. LA F1 A. ahl No

HA F1 A. exi vs. HA F0 A. exi No

HA F1 A. exi vs. LA F0 A. spl No

HA F0 A. exi vs. LA F1 A. ahl No

HA F0 A. cam vs. LA F1 A. spl No

LA F0 A. spl vs. LA F1 A. ahl No

HA F1 A. exi vs. HA F1 A. cam No

HA F1 A. cam vs. LA F1 A. spl No

HA F0 A. cam vs. LA F0 A. ahl No

LA F0 A. ahl vs. LA F1 A. ahl No

HA F1 A. exi vs. HA F0 A. cam No

HA F0 A. exi vs. LA F1 A. spl No

HA F1 A. cam vs. LA F0 A. ahl No

LA F0 A. spl vs. LA F1 A. spl No

HA F0 A. cam vs. HA F0 A. exi No

HA F0 A. cam vs. LA F0 A. spl No

LA F1 A. spl vs. LA F1 A. ahl No

HA F0 A. exi vs. LA F0 A. ahl No

LA F0 A. ahl vs. LA F1 A. spl No

HA F1 A. cam vs. LA F0 A. spl No

HA F1 A. cam vs. HA F0 A. exi No

LA F0 A. spl vs. LA F0 A. ahl No

HA F0 A. cam vs. HA F1 A. cam No

HA F0 A. exi vs. LA F0 A. spl No
