# Supplementary material for: Local Adaptation to Altitude Underlies Divergent Thermal Physiology in Tropical Killifishes of the Genus Aphyosemion
Source: PLoS One. 2013 Jan 22;8(1):e54345. doi: 10.1371/journal.pone.0054345 (PMC3551936; doi:10.1371/journal.pone.0054345)
Supplement: Text S5 — Two Way Analysis of Variance comparing SDA duration at three temperatures among 2 altitudes×2 species×2 generations. (DOC) [file pone.0054345.s005.doc]

**Supporting Information 5**

**Two Way Analysis of Variance comparing SDA duration at three temperatures among 2 altitudes x 2 species x 2 generations**

General Linear Model

Dependent Variable: D_SDA_ in minutes

**Normality Test:** Passed (P = 0.110)

**Equal Variance Test:** Passed (P = 0.670)

**Source of Variation DF SS MS F P**

altitude/generation/species 7 409605.971 58515.139 1.391 0.216

temperature 2 176870.119 88435.060 2.102 0.127

altitude/gene x temperature 14 3582143.057 255867.361 6.082 <0.001

Residual 111 4669981.190 42071.903

Total 134 8834762.104 65931.060

Main effects cannot be properly interpreted if significant interaction is determined. This is because the size of a factor's effect depends upon the level of the other factor.

The effect of different levels of altitude/generation/species depends on what level of temperature is present. There is a statistically significant interaction between altitude/generation/species and temperature . (P = <0.001)

Power of performed test with alpha = 0.0500: for altitude/generation/species : 0.166

Power of performed test with alpha = 0.0500: for temperature : 0.229

Power of performed test with alpha = 0.0500: for altitude/gene x temperature : 1.000

Least square means for altitude/generation/species :

**Group Mean SEM**

HA F0 A. exiguum 587.056 49.931

HA F1 A. exiguum 695.556 52.220

HA F0 A. cameronense 704.667 52.960

HA F1 A. cameronense 535.000 48.346

LA F0 A. ahli 621.500 49.931

LA F1 A. ahli 565.397 47.181

LA F0 A. splendopleure 581.333 52.960

LA F1 A. splendopleure 608.889 48.346

Least square means for temperature :

**Group Mean SEM**

19 626.146 30.337

25 561.854 31.921

28 649.274 30.078

Least square means for altitude/gene x temperature :

**Group Mean SEM**

HA F0 A. exiguum x 19 496.500 83.738

HA F0 A. exiguum x 25 483.000 91.730

HA F0 A. exiguum x 28 781.667 83.738

HA F1 A. exiguum x 19 481.667 83.738

HA F1 A. exiguum x 25 705.000 102.557

HA F1 A. exiguum x 28 900.000 83.738

HA F0 A. cameronense x 19 486.000 91.730

HA F0 A. cameronense x 25 630.000 91.730

HA F0 A. cameronense x 28 998.000 91.730

HA F1 A. cameronense x 19 352.500 83.738

HA F1 A. cameronense x 25 552.500 83.738

HA F1 A. cameronense x 28 700.000 83.738

LA F0 A. ahli x 19 810.000 83.738

LA F0 A. ahli x 25 612.000 91.730

LA F0 A. ahli x 28 442.500 83.738

LA F1 A. ahli x 19 735.000 83.738

LA F1 A. ahli x 25 588.333 83.738

LA F1 A. ahli x 28 372.857 77.526

LA F0 A. sple x 19 750.000 91.730

LA F0 A. sple x 25 454.000 91.730

LA F0 A. sple x 28 540.000 91.730

LA F1 A. sple x 19 897.500 83.738

LA F1 A. sple x 25 470.000 83.738

LA F1 A. sple x 28 459.167 83.738

All Pairwise Multiple Comparison Procedures (Holm-Sidak method):

Overall significance level = 0.05

Comparisons for factor: **altitude/generation/species**

**Comparison Diff of Means t Unadjusted P Critical Level**

HA F0 A. cam vs. HA F1 A. cam 169.667 2.366 0.020 0.002

HA F1 A. exi vs. HA F1 A. cam 160.556 2.256 0.026 0.002

HA F0 A. cam vs. LA F1 A. ahl 139.270 1.964 0.052 0.002

HA F1 A. exi vs. LA F1 A. ahl 130.159 1.849 0.067 0.002

HA F0 A. cam vs. LA F0 A. spl 123.333 1.647 0.102 0.002

HA F0 A. cam vs. HA F0 A. exi 117.611 1.616 0.109 0.002

HA F1 A. exi vs. LA F0 A. spl 114.222 1.536 0.127 0.002

HA F1 A. exi vs. HA F0 A. exi 108.500 1.502 0.136 0.002

HA F0 A. cam vs. LA F1 A. spl 95.778 1.336 0.184 0.003

LA F0 A. ahl vs. HA F1 A. cam 86.500 1.245 0.216 0.003

HA F1 A. exi vs. LA F1 A. spl 86.667 1.218 0.226 0.003

HA F0 A. cam vs. LA F0 A. ahl 83.167 1.143 0.256 0.003

LA F1 A. spl vs. HA F1 A. cam 73.889 1.081 0.282 0.003

HA F1 A. exi vs. LA F0 A. ahl 74.056 1.025 0.308 0.003

LA F0 A. ahl vs. LA F1 A. ahl 56.103 0.817 0.416 0.004

HA F0 A. exi vs. HA F1 A. cam 52.056 0.749 0.455 0.004

LA F0 A. spl vs. HA F1 A. cam 46.333 0.646 0.520 0.004

LA F1 A. spl vs. LA F1 A. ahl 43.492 0.644 0.521 0.005

LA F0 A. ahl vs. LA F0 A. spl 40.167 0.552 0.582 0.005

LA F0 A. ahl vs. HA F0 A. exi 34.444 0.488 0.627 0.006

LA F1 A. ahl vs. HA F1 A. cam 30.397 0.450 0.654 0.006

LA F1 A. spl vs. LA F0 A. spl 27.556 0.384 0.702 0.007

HA F0 A. exi vs. LA F1 A. ahl 21.659 0.315 0.753 0.009

LA F1 A. spl vs. HA F0 A. exi 21.833 0.314 0.754 0.010

LA F0 A. spl vs. LA F1 A. ahl 15.937 0.225 0.823 0.013

LA F0 A. ahl vs. LA F1 A. spl 12.611 0.181 0.856 0.017

HA F0 A. cam vs. HA F1 A. exi 9.111 0.123 0.903 0.025

HA F0 A. exi vs. LA F0 A. spl 5.722 0.0786 0.937 0.050

**Comparison Significant?**

HA F0 A. cam vs. HA F1 A. cam No

HA F1 A. exi vs. HA F1 A. cam No

HA F0 A. cam vs. LA F1 A. ahl No

HA F1 A. exi vs. LA F1 A. ahl No

HA F0 A. cam vs. LA F0 A. spl No

HA F0 A. cam vs. HA F0 A. exi No

HA F1 A. exi vs. LA F0 A. spl No

HA F1 A. exi vs. HA F0 A. exi No

HA F0 A. cam vs. LA F1 A. spl No

LA F0 A. ahl vs. HA F1 A. cam No

HA F1 A. exi vs. LA F1 A. spl No

HA F0 A. cam vs. LA F0 A. ahl No

LA F1 A. spl vs. HA F1 A. cam No

HA F1 A. exi vs. LA F0 A. ahl No

LA F0 A. ahl vs. LA F1 A. ahl No

HA F0 A. exi vs. HA F1 A. cam No

LA F0 A. spl vs. HA F1 A. cam No

LA F1 A. spl vs. LA F1 A. ahl No

LA F0 A. ahl vs. LA F0 A. spl No

LA F0 A. ahl vs. HA F0 A. exi No

LA F1 A. ahl vs. HA F1 A. cam No

LA F1 A. spl vs. LA F0 A. spl No

HA F0 A. exi vs. LA F1 A. ahl No

LA F1 A. spl vs. HA F0 A. exi No

LA F0 A. spl vs. LA F1 A. ahl No

LA F0 A. ahl vs. LA F1 A. spl No

HA F0 A. cam vs. HA F1 A. exi No

HA F0 A. exi vs. LA F0 A. spl No

Comparisons for factor: **temperature**

**Comparison Diff of Means t Unadjusted P Critical Level Significant?**

28 vs. 25 87.420 1.993 0.049 0.017 No

19 vs. 25 64.292 1.460 0.147 0.025 No

28 vs. 19 23.128 0.541 0.589 0.050 No

Comparisons for factor: **temperature within HA F0 A. exiguum**

**Comparison Diff of Means t Unadjusted P Critical Level**

28 vs. 19 285.167 2.408 0.018 0.017

28 vs. 25 298.667 2.405 0.018 0.025

19 vs. 25 13.500 0.109 0.914 0.050

**Comparison Significant?**

28 vs. 19 No

28 vs. 25 Yes

19 vs. 25 No

Comparisons for factor: **temperature within HA F1 A. exiguum**

**Comparison Diff of Means t Unadjusted P Critical Level**

28 vs. 19 418.333 3.533 <0.001 0.017

25 vs. 19 223.333 1.687 0.094 0.025

28 vs. 25 195.000 1.473 0.144 0.050

**Comparison Significant?**

28 vs. 19 Yes

25 vs. 19 No

28 vs. 25 No

Comparisons for factor: **temperature within HA F0 A. cameronense**

**Comparison Diff of Means t Unadjusted P Critical Level**

28 vs. 19 512.000 3.947 <0.001 0.017

28 vs. 25 368.000 2.837 0.005 0.025

25 vs. 19 144.000 1.110 0.269 0.050

**Comparison Significant?**

28 vs. 19 Yes

28 vs. 25 Yes

25 vs. 19 No

Comparisons for factor: **temperature within HA F1 A. cameronense**

**Comparison Diff of Means t Unadjusted P Critical Level**

28 vs. 19 347.500 2.934 0.004 0.017

25 vs. 19 200.000 1.689 0.094 0.025

28 vs. 25 147.500 1.246 0.216 0.050

**Comparison Significant?**

28 vs. 19 Yes

25 vs. 19 No

28 vs. 25 No

Comparisons for factor: **temperature within LA F0 A. ahli**

**Comparison Diff of Means t Unadjusted P Critical Level**

19 vs. 28 367.500 3.103 0.002 0.017

19 vs. 25 198.000 1.594 0.114 0.025

25 vs. 28 169.500 1.365 0.175 0.050

**Comparison Significant?**

19 vs. 28 Yes

19 vs. 25 No

25 vs. 28 No

Comparisons for factor: **temperature within LA F1 A. ahli**

**Comparison Diff of Means t Unadjusted P Critical Level**

19 vs. 28 362.143 3.173 0.002 0.017

25 vs. 28 215.476 1.888 0.062 0.025

19 vs. 25 146.667 1.238 0.218 0.050

**Comparison Significant?**

19 vs. 28 Yes

25 vs. 28 No

19 vs. 25 No

Comparisons for factor: **temperature within LA F0 A. splendopleure**

**Comparison Diff of Means t Unadjusted P Critical Level**

19 vs. 25 296.000 2.282 0.024 0.017

19 vs. 28 210.000 1.619 0.108 0.025

28 vs. 25 86.000 0.663 0.509 0.050

**Comparison Significant?**

19 vs. 25 No

19 vs. 28 No

28 vs. 25 No

Comparisons for factor: **temperature within LA F1 A. splendopleure**

**Comparison Diff of Means t Unadjusted P Critical Level**

19 vs. 28 438.333 3.701 <0.001 0.017

19 vs. 25 427.500 3.610 <0.001 0.025

25 vs. 28 10.833 0.0915 0.927 0.050

**Comparison Significant?**

19 vs. 28 Yes

19 vs. 25 Yes

25 vs. 28 No

Comparisons for factor: **altitude/generation/species within 19**

**Comparison Diff of Means t Unadjusted P Critical Level**

LA F1 A. spl vs. HA F1 A. cam 545.000 4.602 <0.001 0.002

LA F0 A. ahl vs. HA F1 A. cam 457.500 3.863 <0.001 0.002

LA F1 A. spl vs. HA F1 A. exi 415.833 3.511 <0.001 0.002

LA F1 A. spl vs. HA F0 A. exi 401.000 3.386 <0.001 0.002

LA F1 A. spl vs. HA F0 A. cam 411.500 3.313 0.001 0.002

LA F1 A. ahl vs. HA F1 A. cam 382.500 3.230 0.002 0.002

LA F0 A. spl vs. HA F1 A. cam 397.500 3.200 0.002 0.002

LA F0 A. ahl vs. HA F1 A. exi 328.333 2.773 0.007 0.002

LA F0 A. ahl vs. HA F0 A. exi 313.500 2.647 0.009 0.003

LA F0 A. ahl vs. HA F0 A. cam 324.000 2.609 0.010 0.003

LA F0 A. spl vs. HA F1 A. exi 268.333 2.160 0.033 0.003

LA F1 A. ahl vs. HA F1 A. exi 253.333 2.139 0.035 0.003

LA F0 A. spl vs. HA F0 A. exi 253.500 2.041 0.044 0.003

LA F0 A. spl vs. HA F0 A. cam 264.000 2.035 0.044 0.003

LA F1 A. ahl vs. HA F0 A. exi 238.500 2.014 0.046 0.004

LA F1 A. ahl vs. HA F0 A. cam 249.000 2.005 0.047 0.004

LA F1 A. spl vs. LA F1 A. ahl 162.500 1.372 0.173 0.004

HA F0 A. exi vs. HA F1 A. cam 144.000 1.216 0.227 0.005

LA F1 A. spl vs. LA F0 A. spl 147.500 1.188 0.238 0.005

HA F1 A. exi vs. HA F1 A. cam 129.167 1.091 0.278 0.006

HA F0 A. cam vs. HA F1 A. cam 133.500 1.075 0.285 0.006

LA F1 A. spl vs. LA F0 A. ahl 87.500 0.739 0.462 0.007

LA F0 A. ahl vs. LA F1 A. ahl 75.000 0.633 0.528 0.009

LA F0 A. ahl vs. LA F0 A. spl 60.000 0.483 0.630 0.010

HA F0 A. exi vs. HA F1 A. exi 14.833 0.125 0.901 0.013

LA F0 A. spl vs. LA F1 A. ahl 15.000 0.121 0.904 0.017

HA F0 A. exi vs. HA F0 A. cam 10.500 0.0845 0.933 0.025

HA F0 A. cam vs. HA F1 A. exi 4.333 0.0349 0.972 0.050

**Comparison Significant?**

LA F1 A. spl vs. HA F1 A. cam Yes

LA F0 A. ahl vs. HA F1 A. cam Yes

LA F1 A. spl vs. HA F1 A. exi Yes

LA F1 A. spl vs. HA F0 A. exi Yes

LA F1 A. spl vs. HA F0 A. cam Yes

LA F1 A. ahl vs. HA F1 A. cam Yes

LA F0 A. spl vs. HA F1 A. cam Yes

LA F0 A. ahl vs. HA F1 A. exi No

LA F0 A. ahl vs. HA F0 A. exi No

LA F0 A. ahl vs. HA F0 A. cam No

LA F0 A. spl vs. HA F1 A. exi No

LA F1 A. ahl vs. HA F1 A. exi No

LA F0 A. spl vs. HA F0 A. exi No

LA F0 A. spl vs. HA F0 A. cam No

LA F1 A. ahl vs. HA F0 A. exi No

LA F1 A. ahl vs. HA F0 A. cam No

LA F1 A. spl vs. LA F1 A. ahl No

HA F0 A. exi vs. HA F1 A. cam No

LA F1 A. spl vs. LA F0 A. spl No

HA F1 A. exi vs. HA F1 A. cam No

HA F0 A. cam vs. HA F1 A. cam No

LA F1 A. spl vs. LA F0 A. ahl No

LA F0 A. ahl vs. LA F1 A. ahl No

LA F0 A. ahl vs. LA F0 A. spl No

HA F0 A. exi vs. HA F1 A. exi No

LA F0 A. spl vs. LA F1 A. ahl No

HA F0 A. exi vs. HA F0 A. cam No

HA F0 A. cam vs. HA F1 A. exi No

Comparisons for factor: **altitude/generation/species within 25**

**Comparison Diff of Means t Unadjusted P Critical Level**

HA F1 A. exi vs. LA F0 A. spl 251.000 1.824 0.071 0.002

HA F1 A. exi vs. LA F1 A. spl 235.000 1.775 0.079 0.002

HA F1 A. exi vs. HA F0 A. exi 222.000 1.613 0.109 0.002

HA F0 A. cam vs. LA F0 A. spl 176.000 1.357 0.178 0.002

HA F0 A. cam vs. LA F1 A. spl 160.000 1.288 0.200 0.002

LA F0 A. ahl vs. LA F0 A. spl 158.000 1.218 0.226 0.002

HA F1 A. exi vs. HA F1 A. cam 152.500 1.152 0.252 0.002

LA F0 A. ahl vs. LA F1 A. spl 142.000 1.143 0.255 0.002

HA F0 A. cam vs. HA F0 A. exi 147.000 1.133 0.260 0.003

LA F1 A. ahl vs. LA F0 A. spl 134.333 1.082 0.282 0.003

LA F1 A. ahl vs. LA F1 A. spl 118.333 0.999 0.320 0.003

LA F0 A. ahl vs. HA F0 A. exi 129.000 0.994 0.322 0.003

HA F1 A. exi vs. LA F1 A. ahl 116.667 0.881 0.380 0.003

LA F1 A. ahl vs. HA F0 A. exi 105.333 0.848 0.398 0.003

HA F1 A. cam vs. LA F0 A. spl 98.500 0.793 0.429 0.004

HA F1 A. cam vs. LA F1 A. spl 82.500 0.697 0.487 0.004

HA F1 A. exi vs. LA F0 A. ahl 93.000 0.676 0.501 0.004

HA F0 A. cam vs. HA F1 A. cam 77.500 0.624 0.534 0.005

HA F1 A. cam vs. HA F0 A. exi 69.500 0.560 0.577 0.005

HA F1 A. exi vs. HA F0 A. cam 75.000 0.545 0.587 0.006

LA F0 A. ahl vs. HA F1 A. cam 59.500 0.479 0.633 0.006

HA F0 A. cam vs. LA F1 A. ahl 41.667 0.335 0.738 0.007

LA F1 A. ahl vs. HA F1 A. cam 35.833 0.303 0.763 0.009

HA F0 A. exi vs. LA F0 A. spl 29.000 0.224 0.824 0.010

LA F0 A. ahl vs. LA F1 A. ahl 23.667 0.191 0.849 0.013

HA F0 A. cam vs. LA F0 A. ahl 18.000 0.139 0.890 0.017

LA F1 A. spl vs. LA F0 A. spl 16.000 0.129 0.898 0.025

HA F0 A. exi vs. LA F1 A. spl 13.000 0.105 0.917 0.050

**Comparison Significant?**

HA F1 A. exi vs. LA F0 A. spl No

HA F1 A. exi vs. LA F1 A. spl No

HA F1 A. exi vs. HA F0 A. exi No

HA F0 A. cam vs. LA F0 A. spl No

HA F0 A. cam vs. LA F1 A. spl No

LA F0 A. ahl vs. LA F0 A. spl No

HA F1 A. exi vs. HA F1 A. cam No

LA F0 A. ahl vs. LA F1 A. spl No

HA F0 A. cam vs. HA F0 A. exi No

LA F1 A. ahl vs. LA F0 A. spl No

LA F1 A. ahl vs. LA F1 A. spl No

LA F0 A. ahl vs. HA F0 A. exi No

HA F1 A. exi vs. LA F1 A. ahl No

LA F1 A. ahl vs. HA F0 A. exi No

HA F1 A. cam vs. LA F0 A. spl No

HA F1 A. cam vs. LA F1 A. spl No

HA F1 A. exi vs. LA F0 A. ahl No

HA F0 A. cam vs. HA F1 A. cam No

HA F1 A. cam vs. HA F0 A. exi No

HA F1 A. exi vs. HA F0 A. cam No

LA F0 A. ahl vs. HA F1 A. cam No

HA F0 A. cam vs. LA F1 A. ahl No

LA F1 A. ahl vs. HA F1 A. cam No

HA F0 A. exi vs. LA F0 A. spl No

LA F0 A. ahl vs. LA F1 A. ahl No

HA F0 A. cam vs. LA F0 A. ahl No

LA F1 A. spl vs. LA F0 A. spl No

HA F0 A. exi vs. LA F1 A. spl No

Comparisons for factor: **altitude/generation/species within 28**

**Comparison Diff of Means t Unadjusted P Critical Level**

HA F0 A. cam vs. LA F1 A. ahl 625.143 5.205 <0.001 0.002

HA F1 A. exi vs. LA F1 A. ahl 527.143 4.619 <0.001 0.002

HA F0 A. cam vs. LA F0 A. ahl 555.500 4.473 <0.001 0.002

HA F0 A. cam vs. LA F1 A. spl 538.833 4.338 <0.001 0.002

HA F1 A. exi vs. LA F0 A. ahl 457.500 3.863 <0.001 0.002

HA F1 A. exi vs. LA F1 A. spl 440.833 3.723 <0.001 0.002

HA F0 A. exi vs. LA F1 A. ahl 408.810 3.582 <0.001 0.002

HA F0 A. cam vs. LA F0 A. spl 458.000 3.531 <0.001 0.002

HA F1 A. exi vs. LA F0 A. spl 360.000 2.898 0.005 0.003

HA F1 A. cam vs. LA F1 A. ahl 327.143 2.867 0.005 0.003

HA F0 A. exi vs. LA F0 A. ahl 339.167 2.864 0.005 0.003

HA F0 A. exi vs. LA F1 A. spl 322.500 2.723 0.008 0.003

HA F0 A. cam vs. HA F1 A. cam 298.000 2.399 0.018 0.003

HA F1 A. cam vs. LA F0 A. ahl 257.500 2.174 0.032 0.003

HA F1 A. cam vs. LA F1 A. spl 240.833 2.034 0.044 0.004

HA F0 A. exi vs. LA F0 A. spl 241.667 1.946 0.054 0.004

HA F0 A. cam vs. HA F0 A. exi 216.333 1.742 0.084 0.004

HA F1 A. exi vs. HA F1 A. cam 200.000 1.689 0.094 0.005

LA F0 A. spl vs. LA F1 A. ahl 167.143 1.392 0.167 0.005

HA F1 A. cam vs. LA F0 A. spl 160.000 1.288 0.200 0.006

HA F1 A. exi vs. HA F0 A. exi 118.333 0.999 0.320 0.006

HA F0 A. cam vs. HA F1 A. exi 98.000 0.789 0.432 0.007

LA F0 A. spl vs. LA F0 A. ahl 97.500 0.785 0.434 0.009

LA F1 A. spl vs. LA F1 A. ahl 86.310 0.756 0.451 0.010

HA F0 A. exi vs. HA F1 A. cam 81.667 0.690 0.492 0.013

LA F0 A. spl vs. LA F1 A. spl 80.833 0.651 0.517 0.017

LA F0 A. ahl vs. LA F1 A. ahl 69.643 0.610 0.543 0.025

LA F1 A. spl vs. LA F0 A. ahl 16.667 0.141 0.888 0.050

**Comparison Significant?**

HA F0 A. cam vs. LA F1 A. ahl Yes

HA F1 A. exi vs. LA F1 A. ahl Yes

HA F0 A. cam vs. LA F0 A. ahl Yes

HA F0 A. cam vs. LA F1 A. spl Yes

HA F1 A. exi vs. LA F0 A. ahl Yes

HA F1 A. exi vs. LA F1 A. spl Yes

HA F0 A. exi vs. LA F1 A. ahl Yes

HA F0 A. cam vs. LA F0 A. spl Yes

HA F1 A. exi vs. LA F0 A. spl No

HA F1 A. cam vs. LA F1 A. ahl No

HA F0 A. exi vs. LA F0 A. ahl No

HA F0 A. exi vs. LA F1 A. spl No

HA F0 A. cam vs. HA F1 A. cam No

HA F1 A. cam vs. LA F0 A. ahl No

HA F1 A. cam vs. LA F1 A. spl No

HA F0 A. exi vs. LA F0 A. spl No

HA F0 A. cam vs. HA F0 A. exi No

HA F1 A. exi vs. HA F1 A. cam No

LA F0 A. spl vs. LA F1 A. ahl No

HA F1 A. cam vs. LA F0 A. spl No

HA F1 A. exi vs. HA F0 A. exi No

HA F0 A. cam vs. HA F1 A. exi No

LA F0 A. spl vs. LA F0 A. ahl No

LA F1 A. spl vs. LA F1 A. ahl No

HA F0 A. exi vs. HA F1 A. cam No

LA F0 A. spl vs. LA F1 A. spl No

LA F0 A. ahl vs. LA F1 A. ahl No

LA F1 A. spl vs. LA F0 A. ahl No
