# Supplementary material for: Local Adaptation to Altitude Underlies Divergent Thermal Physiology in Tropical Killifishes of the Genus Aphyosemion
Source: PLoS One. 2013 Jan 22;8(1):e54345. doi: 10.1371/journal.pone.0054345 (PMC3551936; doi:10.1371/journal.pone.0054345)
Supplement: Text S6 — Two Way Analysis of Variance comparing SDA coefficient at three temperatures among 2 altitudes×2 species×2 generations. (DOC) [file pone.0054345.s006.doc]

**Supporting Information 6**

**Two Way Analysis of Variance comparing SDA coefficient at three temperatures among 2 altitudes x 2 species x 2 generations**

General Linear Model

Dependent Variable: Log10(C_SDA_) in μg 0_2_

**Normality Test:** Passed (P = 0.213)

**Equal Variance Test:** Passed (P = 0.247)

**Source of Variation DF SS MS F P**

altitude/generation/species 7 0.735 0.105 1.663 0.125

temperature 2 0.178 0.0888 1.406 0.250

altitude/gene x temperature 14 1.315 0.0940 1.488 0.127

Residual 111 7.009 0.0631

Total 134 9.211 0.0687

The difference in the mean values among the different levels of altitude/generation/species is not great enough to exclude the possibility that the difference is just due to random sampling variability after allowing for the effects of differences in temperature . There is not a statistically significant difference (P = 0.125).

The difference in the mean values among the different levels of temperature is not great enough to exclude the possibility that the difference is just due to random sampling variability after allowing for the effects of differences in altitude/generation/species. There is not a statistically significant difference (P = 0.250).

The effect of different levels of altitude/generation/species does not depend on what level of temperature is present. There is not a statistically significant interaction between altitude/generation/species and temperature . (P = 0.127)

Power of performed test with alpha = 0.0500: for altitude/generation/species : 0.270

Power of performed test with alpha = 0.0500: for temperature : 0.110

Power of performed test with alpha = 0.0500: for altitude/gene x temperature : 0.282

Least square means for altitude/generation/species :

**Group Mean SEM**

HA F0 A. exiguum 2.841 0.0612

HA F1 A. exiguum 2.940 0.0640

HA F0 A. cameronense 2.810 0.0649

HA F1 A. cameronense 2.868 0.0592

LA F0 A. ahli 2.914 0.0612

LA F1 A. ahli 2.940 0.0578

LA F0 A. splendopleure 2.785 0.0649

LA F1 A. splendopleure 3.026 0.0592

Least square means for temperature :

**Group Mean SEM**

19 2.844 0.0372

25 2.895 0.0391

28 2.932 0.0368

Least square means for altitude/gene x temperature :

**Group Mean SEM**

HA F0 A. exiguum x 19 2.787 0.103

HA F0 A. exiguum x 25 2.690 0.112

HA F0 A. exiguum x 28 3.046 0.103

HA F1 A. exiguum x 19 2.832 0.103

HA F1 A. exiguum x 25 2.933 0.126

HA F1 A. exiguum x 28 3.054 0.103

HA F0 A. cameronense x 19 2.778 0.112

HA F0 A. cameronense x 25 2.931 0.112

HA F0 A. cameronense x 28 2.722 0.112

HA F1 A. cameronense x 19 2.676 0.103

HA F1 A. cameronense x 25 2.888 0.103

HA F1 A. cameronense x 28 3.042 0.103

LA F0 A. ahli x 19 2.898 0.103

LA F0 A. ahli x 25 3.018 0.112

LA F0 A. ahli x 28 2.827 0.103

LA F1 A. ahli x 19 2.920 0.103

LA F1 A. ahli x 25 3.048 0.103

LA F1 A. ahli x 28 2.852 0.0950

LA F0 A. sple x 19 2.739 0.112

LA F0 A. sple x 25 2.713 0.112

LA F0 A. sple x 28 2.902 0.112

LA F1 A. sple x 19 3.126 0.103

LA F1 A. sple x 25 2.941 0.103

LA F1 A. sple x 28 3.010 0.103
