# Supplementary material for: Local Adaptation to Altitude Underlies Divergent Thermal Physiology in Tropical Killifishes of the Genus Aphyosemion
Source: PLoS One. 2013 Jan 22;8(1):e54345. doi: 10.1371/journal.pone.0054345 (PMC3551936; doi:10.1371/journal.pone.0054345)
Supplement: Text S7 — Two Way Analysis of Variance. comparing routine metabolic rate at three temperatures among 2 altitude groups×2 generations. (DOC) [file pone.0054345.s007.doc]

**Supporting Information 7**

**Two Way Analysis of Variance. comparing routine metabolic rate at three temperatures among 2 altitude groups x 2 generations**

General Linear Model

Dependent Variable: Log10(RMR) in μg O_2_ h^-1^

**Normality Test:** Passed (P = 0.103)

**Equal Variance Test:** Passed (P = 0.148)

**Source of Variation DF SS MS F P**

altitude/generation 3 0.216 0.0718 4.054 0.009

temperature 2 1.593 0.796 44.940 <0.001

altitude/generation x temperature 6 0.0677 0.0113 0.637 0.700

Residual 123 2.179 0.0177

Total 134 4.058 0.0303

The difference in the mean values among the different levels of altitude/generation is greater than would be expected by chance after allowing for effects of differences in temperature . There is a statistically significant difference (P = 0.009). To isolate which group(s) differ from the others use a multiple comparison procedure.

The difference in the mean values among the different levels of temperature is greater than would be expected by chance after allowing for effects of differences in altitude/generation. There is a statistically significant difference (P = <0.001). To isolate which group(s) differ from the others use a multiple comparison procedure.

The effect of different levels of altitude/generation does not depend on what level of temperature is present. There is not a statistically significant interaction between altitude/generation and temperature . (P = 0.700)

Power of performed test with alpha = 0.0500: for altitude/generation : 0.705

Power of performed test with alpha = 0.0500: for temperature : 1.000

Power of performed test with alpha = 0.0500: for altitude/generation x temperature : 0.0500

Least square means for altitude/generation :

**Group Mean SEM**

HA F0 2.031 0.0236

HA F1 1.940 0.0229

LA F0 1.982 0.0225

LA F1 1.928 0.0228

Least square means for temperature :

**Group Mean SEM**

19 1.837 0.0196

25 1.976 0.0206

28 2.099 0.0194

Least square means for altitude/generation x temperature :

**Group Mean SEM**

HA F0 x 19 1.901 0.0401

HA F0 x 25 2.064 0.0421

HA F0 x 28 2.129 0.0401

HA F1 x 19 1.773 0.0384

HA F1 x 25 1.975 0.0421

HA F1 x 28 2.073 0.0384

LA F0 x 19 1.867 0.0384

LA F0 x 25 1.961 0.0401

LA F0 x 28 2.118 0.0384

LA F1 x 19 1.806 0.0401

LA F1 x 25 1.904 0.0401

LA F1 x 28 2.074 0.0384

All Pairwise Multiple Comparison Procedures (Holm-Sidak method):

Overall significance level = 0.05

Comparisons for factor: **altitude/generation**

**Comparison Diff of Means t Unadjusted P Critical Level Significant?**

HA F0 vs. LA F1 0.104 3.155 0.002 0.009 Yes

HA F0 vs. HA F1 0.0910 2.768 0.007 0.010 Yes

LA F0 vs. LA F1 0.0545 1.699 0.092 0.013 No

HA F0 vs. LA F0 0.0490 1.505 0.135 0.017 No

LA F0 vs. HA F1 0.0419 1.305 0.194 0.025 No

HA F1 vs. LA F1 0.0126 0.389 0.698 0.050 No

Comparisons for factor: **temperature**

**Comparison Diff of Means t Unadjusted P Critical Level Significant?**

28 vs. 19 0.262 9.476 <0.001 0.017 Yes

25 vs. 19 0.139 4.900 <0.001 0.025 Yes

28 vs. 25 0.122 4.330 <0.001 0.050 Yes

Comparisons for factor: **temperature within HA F0**

**Comparison Diff of Means t Unadjusted P Critical Level Significant?**

28 vs. 19 0.227 4.007 <0.001 0.017 Yes

25 vs. 19 0.162 2.792 0.006 0.025 Yes

28 vs. 25 0.0651 1.119 0.265 0.050 No

Comparisons for factor: **temperature within HA F1**

**Comparison Diff of Means t Unadjusted P Critical Level Significant?**

28 vs. 19 0.301 5.530 <0.001 0.017 Yes

25 vs. 19 0.203 3.555 <0.001 0.025 Yes

28 vs. 25 0.0979 1.718 0.088 0.050 No

Comparisons for factor: **temperature within LA F0**

**Comparison Diff of Means t Unadjusted P Critical Level Significant?**

28 vs. 19 0.251 4.626 <0.001 0.017 Yes

28 vs. 25 0.157 2.827 0.005 0.025 Yes

25 vs. 19 0.0943 1.697 0.092 0.050 No

Comparisons for factor: **temperature within LA F1**

**Comparison Diff of Means t Unadjusted P Critical Level Significant?**

28 vs. 19 0.268 4.824 <0.001 0.017 Yes

28 vs. 25 0.170 3.059 0.003 0.025 Yes

25 vs. 19 0.0981 1.728 0.087 0.050 No

Comparisons for factor: **altitude/generation within 19**

**Comparison Diff of Means t Unadjusted P Critical Level Significant?**

HA F0 vs. HA F1 0.129 2.317 0.022 0.009 No

LA F0 vs. HA F1 0.0944 1.737 0.085 0.010 No

HA F0 vs. LA F1 0.0957 1.685 0.094 0.013 No

LA F0 vs. LA F1 0.0613 1.104 0.272 0.017 No

HA F0 vs. LA F0 0.0343 0.618 0.538 0.025 No

LA F1 vs. HA F1 0.0331 0.595 0.553 0.050 No

Comparisons for factor: **altitude/generation within 25**

**Comparison Diff of Means t Unadjusted P Critical Level Significant?**

HA F0 vs. LA F1 0.160 2.750 0.007 0.009 Yes

HA F0 vs. LA F0 0.102 1.760 0.081 0.010 No

HA F0 vs. HA F1 0.0885 1.486 0.140 0.013 No

HA F1 vs. LA F1 0.0715 1.229 0.222 0.017 No

LA F0 vs. LA F1 0.0576 1.014 0.312 0.025 No

HA F1 vs. LA F0 0.0139 0.239 0.812 0.050 No

Comparisons for factor: **altitude/generation within 28**

**Comparison Diff of Means t Unadjusted P Critical Level Significant?**

HA F0 vs. HA F1 0.0557 1.002 0.318 0.009 No

HA F0 vs. LA F1 0.0550 0.990 0.324 0.010 No

LA F0 vs. HA F1 0.0453 0.833 0.406 0.013 No

LA F0 vs. LA F1 0.0446 0.821 0.413 0.017 No

HA F0 vs. LA F0 0.0104 0.187 0.852 0.025 No

LA F1 vs. HA F1 0.000635 0.0117 0.991 0.050 No
