# Supplementary material for: Local Adaptation to Altitude Underlies Divergent Thermal Physiology in Tropical Killifishes of the Genus Aphyosemion
Source: PLoS One. 2013 Jan 22;8(1):e54345. doi: 10.1371/journal.pone.0054345 (PMC3551936; doi:10.1371/journal.pone.0054345)
Supplement: Text S8 — Two Way Analysis of Variance comparing peak SDA MO2 at three temperatures among 2 altitude groups×2 generations. (DOC) [file pone.0054345.s008.doc]

**Supporting Information 8**

**Two Way Analysis of Variance comparing peak SDA MO_2_ at three temperatures among 2 altitude groups x 2 generations**

Dependent Variable: MO_2peak_ in μg O_2_ h^-1^

**Normality Test:** Passed (P = 0.215)

**Equal Variance Test:** Passed (P = 0.339)

**Source of Variation DF SS MS F P**

altitude/generation 3 18557.752 6185.917 0.926 0.430

temperature 2 81004.864 40502.432 6.064 0.003

altitude/generation x temperature 6 9645.972 1607.662 0.241 0.962

Residual 123 821510.076 6678.944

Total 134 932399.721 6958.207

The difference in the mean values among the different levels of altitude/generation is not great enough to exclude the possibility that the difference is just due to random sampling variability after allowing for the effects of differences in temperature . There is not a statistically significant difference (P = 0.430).

The difference in the mean values among the different levels of temperature is greater than would be expected by chance after allowing for effects of differences in altitude/generation. There is a statistically significant difference (P = 0.003). To isolate which group(s) differ from the others use a multiple comparison procedure.

The effect of different levels of altitude/generation does not depend on what level of temperature is present. There is not a statistically significant interaction between altitude/generation and temperature . (P = 0.962)

Power of performed test with alpha = 0.0500: for altitude/generation : 0.0500

Power of performed test with alpha = 0.0500: for temperature : 0.820

Power of performed test with alpha = 0.0500: for altitude/generation x temperature : 0.0500

Least square means for altitude/generation :

**Group Mean SEM**

HA F0 221.660 14.462

HA F1 214.701 14.068

LA F0 229.318 13.826

LA F1 246.202 14.027

Least square means for temperature :

**Group Mean SEM**

19 197.572 12.061

25 258.356 12.625

28 227.983 11.929

Least square means for altitude/generation x temperature :

**Group Mean SEM**

HA F0 x 19 186.485 24.641

HA F0 x 25 245.054 25.844

HA F0 x 28 233.442 24.641

HA F1 x 19 187.688 23.592

HA F1 x 25 237.275 25.844

HA F1 x 28 219.140 23.592

LA F0 x 19 209.706 23.592

LA F0 x 25 263.357 24.641

LA F0 x 28 214.891 23.592

LA F1 x 19 206.410 24.641

LA F1 x 25 287.735 24.641

LA F1 x 28 244.461 23.592

All Pairwise Multiple Comparison Procedures (Holm-Sidak method):

Overall significance level = 0.05

Comparisons for factor: **altitude/generation**

**Comparison Diff of Means t Unadjusted P Critical Level Significant?**

LA F1 vs. HA F1 31.501 1.586 0.115 0.009 No

LA F1 vs. HA F0 24.542 1.218 0.226 0.010 No

LA F1 vs. LA F0 16.884 0.857 0.393 0.013 No

LA F0 vs. HA F1 14.617 0.741 0.460 0.017 No

LA F0 vs. HA F0 7.658 0.383 0.703 0.025 No

HA F0 vs. HA F1 6.959 0.345 0.731 0.050 No

Comparisons for factor: **temperature**

**Comparison Diff of Means t Unadjusted P Critical Level Significant?**

25 vs. 19 60.783 3.481 <0.001 0.017 Yes

28 vs. 19 30.411 1.793 0.075 0.025 No

25 vs. 28 30.372 1.749 0.083 0.050 No

Comparisons for factor: **temperature within HA F0**

**Comparison Diff of Means t Unadjusted P Critical Level Significant?**

25 vs. 19 58.569 1.640 0.104 0.017 No

28 vs. 19 46.956 1.347 0.180 0.025 No

25 vs. 28 11.613 0.325 0.746 0.050 No

Comparisons for factor: **temperature within HA F1**

**Comparison Diff of Means t Unadjusted P Critical Level Significant?**

25 vs. 19 49.587 1.417 0.159 0.017 No

28 vs. 19 31.452 0.943 0.348 0.025 No

25 vs. 28 18.135 0.518 0.605 0.050 No

Comparisons for factor: **temperature within LA F0**

**Comparison Diff of Means t Unadjusted P Critical Level Significant?**

25 vs. 19 53.652 1.573 0.118 0.017 No

25 vs. 28 48.466 1.421 0.158 0.025 No

28 vs. 19 5.186 0.155 0.877 0.050 No

Comparisons for factor: **temperature within LA F1**

**Comparison Diff of Means t Unadjusted P Critical Level Significant?**

25 vs. 19 81.326 2.334 0.021 0.017 No

25 vs. 28 43.275 1.269 0.207 0.025 No

28 vs. 19 38.051 1.115 0.267 0.050 No

Comparisons for factor: **altitude/generation within 19**

**Comparison Diff of Means t Unadjusted P Critical Level Significant?**

LA F0 vs. HA F0 23.220 0.681 0.497 0.009 No

LA F0 vs. HA F1 22.018 0.660 0.511 0.010 No

LA F1 vs. HA F0 19.925 0.572 0.569 0.013 No

LA F1 vs. HA F1 18.722 0.549 0.584 0.017 No

LA F0 vs. LA F1 3.296 0.0966 0.923 0.025 No

HA F1 vs. HA F0 1.203 0.0353 0.972 0.050 No

Comparisons for factor: **altitude/generation within 25**

**Comparison Diff of Means t Unadjusted P Critical Level Significant?**

LA F1 vs. HA F1 50.460 1.413 0.160 0.009 No

LA F1 vs. HA F0 42.681 1.195 0.234 0.010 No

LA F0 vs. HA F1 26.082 0.730 0.467 0.013 No

LA F1 vs. LA F0 24.378 0.700 0.486 0.017 No

LA F0 vs. HA F0 18.303 0.513 0.609 0.025 No

HA F0 vs. HA F1 7.779 0.213 0.832 0.050 No

Comparisons for factor: **altitude/generation within 28**

**Comparison Diff of Means t Unadjusted P Critical Level Significant?**

LA F1 vs. LA F0 29.569 0.886 0.377 0.009 No

LA F1 vs. HA F1 25.321 0.759 0.449 0.010 No

HA F0 vs. LA F0 18.550 0.544 0.588 0.013 No

HA F0 vs. HA F1 14.301 0.419 0.676 0.017 No

LA F1 vs. HA F0 11.019 0.323 0.747 0.025 No

HA F1 vs. LA F0 4.249 0.127 0.899 0.050 No
