# Supplementary material for: Local Adaptation to Altitude Underlies Divergent Thermal Physiology in Tropical Killifishes of the Genus Aphyosemion
Source: PLoS One. 2013 Jan 22;8(1):e54345. doi: 10.1371/journal.pone.0054345 (PMC3551936; doi:10.1371/journal.pone.0054345)
Supplement: Text S9 — Two Way Analysis of Variance comparing time to SDA peak at three temperatures among 2 altitude groups×2 generations. (DOC) [file pone.0054345.s009.doc]

**Supporting Information 9**

**Two Way Analysis of Variance comparing time to SDA peak at three temperatures among 2 altitude groups x 2 generations**

General Linear Model

Dependent Variable: Log10(T_peak_) in minutes

**Normality Test:** Passed (P = 0.647)

**Equal Variance Test:** Passed (P = 0.073)

**Source of Variation DF SS MS F P**

altitude/generation 3 0.284 0.0946 0.849 0.470

temperature 2 1.483 0.742 6.652 0.002

altitude/generation x temperature 6 4.024 0.671 6.015 <0.001

Residual 123 13.713 0.111

Total 134 19.472 0.145

Main effects cannot be properly interpreted if significant interaction is determined. This is because the size of a factor's effect depends upon the level of the other factor.

The effect of different levels of altitude/generation depends on what level of temperature is present. There is a statistically significant interaction between altitude/generation and temperature . (P = <0.001)

Power of performed test with alpha = 0.0500: for altitude/generation : 0.050

Power of performed test with alpha = 0.0500: for temperature : 0.866

Power of performed test with alpha = 0.0500: for altitude/generation x temperature : 0.994

Least square means for altitude/generation :

**Group Mean SEM**

HA F0 2.048 0.0591

HA F1 2.042 0.0575

LA F0 1.953 0.0565

LA F1 2.073 0.0573

Least square means for temperature :

**Group Mean SEM**

19 2.053 0.0493

25 1.889 0.0516

28 2.146 0.0487

Least square means for altitude/generation x temperature :

**Group Mean SEM**

HA F0 x 19 1.909 0.101

HA F0 x 25 2.000 0.106

HA F0 x 28 2.235 0.101

HA F1 x 19 1.821 0.0964

HA F1 x 25 1.874 0.106

HA F1 x 28 2.432 0.0964

LA F0 x 19 2.166 0.0964

LA F0 x 25 1.710 0.101

LA F0 x 28 1.984 0.0964

LA F1 x 19 2.315 0.101

LA F1 x 25 1.972 0.101

LA F1 x 28 1.932 0.0964

All Pairwise Multiple Comparison Procedures (Holm-Sidak method):

Overall significance level = 0.05

Comparisons for factor: **altitude/generation**

**Comparison Diff of Means t Unadjusted P Critical Level Significant?**

LA F1 vs. LA F0 0.120 1.492 0.138 0.009 No

HA F0 vs. LA F0 0.0946 1.157 0.249 0.010 No

HA F1 vs. LA F0 0.0886 1.100 0.274 0.013 No

LA F1 vs. HA F1 0.0314 0.387 0.700 0.017 No

LA F1 vs. HA F0 0.0254 0.309 0.758 0.025 No

HA F0 vs. HA F1 0.00597 0.0724 0.942 0.050 No

Comparisons for factor: **temperature**

**Comparison Diff of Means t Unadjusted P Critical Level Significant?**

28 vs. 25 0.257 3.618 <0.001 0.017 Yes

19 vs. 25 0.164 2.296 0.023 0.025 Yes

28 vs. 19 0.0929 1.341 0.182 0.050 No

Comparisons for factor: **temperature within HA F0**

**Comparison Diff of Means t Unadjusted P Critical Level Significant?**

28 vs. 19 0.326 2.288 0.024 0.017 No

28 vs. 25 0.234 1.607 0.111 0.025 No

25 vs. 19 0.0913 0.626 0.532 0.050 No

Comparisons for factor: **temperature within HA F1**

**Comparison Diff of Means t Unadjusted P Critical Level Significant?**

28 vs. 19 0.611 4.482 <0.001 0.017 Yes

28 vs. 25 0.558 3.902 <0.001 0.025 Yes

25 vs. 19 0.0531 0.371 0.711 0.050 No

Comparisons for factor: **temperature within LA F0**

**Comparison Diff of Means t Unadjusted P Critical Level Significant?**

19 vs. 25 0.457 3.277 0.001 0.017 Yes

28 vs. 25 0.275 1.971 0.051 0.025 No

19 vs. 28 0.182 1.335 0.184 0.050 No

Comparisons for factor: **temperature within LA F1**

**Comparison Diff of Means t Unadjusted P Critical Level Significant?**

19 vs. 28 0.383 2.747 0.007 0.017 Yes

19 vs. 25 0.343 2.409 0.017 0.025 Yes

25 vs. 28 0.0399 0.287 0.775 0.050 No

Comparisons for factor: **altitude/generation within 19**

**Comparison Diff of Means t Unadjusted P Critical Level Significant?**

LA F1 vs. HA F1 0.495 3.549 <0.001 0.009 Yes

LA F1 vs. HA F0 0.406 2.855 0.005 0.010 Yes

LA F0 vs. HA F1 0.346 2.535 0.012 0.013 Yes

LA F0 vs. HA F0 0.257 1.846 0.067 0.017 No

LA F1 vs. LA F0 0.149 1.070 0.287 0.025 No

HA F0 vs. HA F1 0.0883 0.633 0.528 0.050 No

Comparisons for factor: **altitude/generation within 25**

**Comparison Diff of Means t Unadjusted P Critical Level Significant?**

HA F0 vs. LA F0 0.291 1.992 0.049 0.009 No

LA F1 vs. LA F0 0.263 1.846 0.067 0.010 No

HA F1 vs. LA F0 0.164 1.125 0.263 0.013 No

HA F0 vs. HA F1 0.127 0.847 0.399 0.017 No

LA F1 vs. HA F1 0.0986 0.676 0.500 0.025 No

HA F0 vs. LA F1 0.0279 0.191 0.849 0.050 No

Comparisons for factor: **altitude/generation within 28**

**Comparison Diff of Means t Unadjusted P Critical Level Significant?**

HA F1 vs. LA F1 0.499 3.662 <0.001 0.009 Yes

HA F1 vs. LA F0 0.447 3.282 0.001 0.010 Yes

HA F0 vs. LA F1 0.302 2.169 0.032 0.013 No

HA F0 vs. LA F0 0.250 1.797 0.075 0.017 No

HA F1 vs. HA F0 0.197 1.413 0.160 0.025 No

LA F0 vs. LA F1 0.0518 0.380 0.705 0.050 No
