# Supplementary material for: Local Adaptation to Altitude Underlies Divergent Thermal Physiology in Tropical Killifishes of the Genus Aphyosemion
Source: PLoS One. 2013 Jan 22;8(1):e54345. doi: 10.1371/journal.pone.0054345 (PMC3551936; doi:10.1371/journal.pone.0054345)
Supplement: Text S10 — Two Way Analysis of Variance comparing SDA duration at three temperatures among 2 altitude groups×2 generations. (DOC) [file pone.0054345.s010.doc]

**Supporting Information 10**

**Two Way Analysis of Variance comparing SDA duration at three temperatures among 2 altitude groups x 2 generations**

General Linear Model

Dependent Variable: D_SDA_ in minutes

**Normality Test:** Passed (P = 0.667)

**Equal Variance Test:** Passed (P = 0.082)

**Source of Variation DF SS MS F P**

altitude/generation 3 87195.942 29065.314 0.676 0.568

temperature 2 177438.818 88719.409 2.064 0.131

altitude/generation x temperature 6 3274675.996 545779.333 12.695 <0.001

Residual 123 5287913.561 42991.167

Total 134 8834762.104 65931.060

Main effects cannot be properly interpreted if significant interaction is determined. This is because the size of a factor's effect depends upon the level of the other factor.

The effect of different levels of altitude/generation depends on what level of temperature is present. There is a statistically significant interaction between altitude/generation and temperature. (P = <0.001)

Power of performed test with alpha = 0.0500: for altitude/generation : 0.050

Power of performed test with alpha = 0.0500: for temperature : 0.223

Power of performed test with alpha = 0.0500: for altitude/generation x temperature : 1.000

Least square means for altitude/generation :

**Group Mean SEM**

HA F0 642.742 36.690

HA F1 610.194 35.691

LA F0 613.043 35.077

LA F1 570.530 35.589

Least square means for temperature :

**Group Mean SEM**

19 626.095 30.600

25 557.955 32.030

28 643.333 30.266

Least square means for altitude/generation x temperature :

**Group Mean SEM**

HA F0 x 19 491.727 62.516

HA F0 x 25 556.500 65.568

HA F0 x 28 880.000 62.516

HA F1 x 19 417.083 59.855

HA F1 x 25 613.500 65.568

HA F1 x 28 800.000 59.855

LA F0 x 19 853.750 59.855

LA F0 x 25 534.545 62.516

LA F0 x 28 450.833 59.855

LA F1 x 19 741.818 62.516

LA F1 x 25 527.273 62.516

LA F1 x 28 442.500 59.855

All Pairwise Multiple Comparison Procedures (Holm-Sidak method):

Overall significance level = 0.05

Comparisons for factor: **altitude/generation**

**Comparison Diff of Means t Unadjusted P Critical Level Significant?**

HA F0 vs. LA F1 72.212 1.413 0.160 0.009 No

LA F0 vs. LA F1 42.513 0.851 0.397 0.010 No

HA F1 vs. LA F1 39.664 0.787 0.433 0.013 No

HA F0 vs. HA F1 32.548 0.636 0.526 0.017 No

HA F0 vs. LA F0 29.699 0.585 0.560 0.025 No

LA F0 vs. HA F1 2.848 0.0569 0.955 0.050 No

Comparisons for factor: **temperature**

**Comparison Diff of Means t Unadjusted P Critical Level Significant?**

28 vs. 25 85.379 1.937 0.055 0.017 No

19 vs. 25 68.140 1.538 0.127 0.025 No

28 vs. 19 17.239 0.401 0.689 0.050 No

Comparisons for factor: **temperature within HA F0**

**Comparison Diff of Means t Unadjusted P Critical Level Significant?**

28 vs. 19 388.273 4.392 <0.001 0.017 Yes

28 vs. 25 323.500 3.571 <0.001 0.025 Yes

25 vs. 19 64.773 0.715 0.476 0.050 No

Comparisons for factor: **temperature within HA F1**

**Comparison Diff of Means t Unadjusted P Critical Level Significant?**

28 vs. 19 382.917 4.524 <0.001 0.017 Yes

25 vs. 19 196.417 2.212 0.029 0.025 No

28 vs. 25 186.500 2.101 0.038 0.050 Yes

Comparisons for factor: **temperature within LA F0**

**Comparison Diff of Means t Unadjusted P Critical Level Significant?**

19 vs. 28 402.917 4.760 <0.001 0.017 Yes

19 vs. 25 319.205 3.688 <0.001 0.025 Yes

25 vs. 28 83.712 0.967 0.335 0.050 No

Comparisons for factor: **temperature within LA F1**

**Comparison Diff of Means t Unadjusted P Critical Level Significant?**

19 vs. 28 299.318 3.458 <0.001 0.017 Yes

19 vs. 25 214.545 2.427 0.017 0.025 Yes

25 vs. 28 84.773 0.979 0.329 0.050 No

Comparisons for factor: **altitude/generation within 19**

**Comparison Diff of Means t Unadjusted P Critical Level Significant?**

LA F0 vs. HA F1 436.667 5.159 <0.001 0.009 Yes

LA F0 vs. HA F0 362.023 4.183 <0.001 0.010 Yes

LA F1 vs. HA F1 324.735 3.752 <0.001 0.013 Yes

LA F1 vs. HA F0 250.091 2.829 0.005 0.017 Yes

LA F0 vs. LA F1 111.932 1.293 0.198 0.025 No

HA F0 vs. HA F1 74.644 0.862 0.390 0.050 No

Comparisons for factor: **altitude/generation within 25**

**Comparison Diff of Means t Unadjusted P Critical Level Significant?**

HA F1 vs. LA F1 86.227 0.952 0.343 0.009 No

HA F1 vs. LA F0 78.955 0.872 0.385 0.010 No

HA F1 vs. HA F0 57.000 0.615 0.540 0.013 No

HA F0 vs. LA F1 29.227 0.323 0.748 0.017 No

HA F0 vs. LA F0 21.955 0.242 0.809 0.025 No

LA F0 vs. LA F1 7.273 0.0823 0.935 0.050 No

Comparisons for factor: **altitude/generation within 28**

**Comparison Diff of Means t Unadjusted P Critical Level Significant?**

HA F0 vs. LA F1 437.500 5.055 <0.001 0.009 Yes

HA F0 vs. LA F0 429.167 4.959 <0.001 0.010 Yes

HA F1 vs. LA F1 357.500 4.223 <0.001 0.013 Yes

HA F1 vs. LA F0 349.167 4.125 <0.001 0.017 Yes

HA F0 vs. HA F1 80.000 0.924 0.357 0.025 No

LA F0 vs. LA F1 8.333 0.0984 0.922 0.050 No
