# Supplementary material for: Local Adaptation to Altitude Underlies Divergent Thermal Physiology in Tropical Killifishes of the Genus Aphyosemion
Source: PLoS One. 2013 Jan 22;8(1):e54345. doi: 10.1371/journal.pone.0054345 (PMC3551936; doi:10.1371/journal.pone.0054345)
Supplement: Text S11 — Two Way Analysis of Variance comparing SDA coefficient at three temperatures among 2 altitude groups×2 generations. (DOC) [file pone.0054345.s011.doc]

**Supporting Information 11**

**Two Way Analysis of Variance comparing SDA coefficient at three temperatures among 2 altitude groups x 2 generations**

General Linear Model

Dependent Variable: Log10(C_SDA_) in μg 0_2_

**Normality Test:** Passed (P = 0.171)

**Equal Variance Test:** Passed (P = 0.065)

**Source of Variation DF SS MS F P**

altitude/generation 3 0.348 0.116 1.739 0.163

temperature 2 0.180 0.0902 1.354 0.262

altitude/generation x temperature 6 0.491 0.0818 1.227 0.297

Residual 123 8.196 0.0666

Total 134 9.211 0.0687

The difference in the mean values among the different levels of altitude/generation is not great enough to exclude the possibility that the difference is just due to random sampling variability after allowing for the effects of differences in temperature . There is not a statistically significant difference (P = 0.163).

The difference in the mean values among the different levels of temperature is not great enough to exclude the possibility that the difference is just due to random sampling variability after allowing for the effects of differences in altitude/generation. There is not a statistically significant difference (P = 0.262).

The effect of different levels of altitude/generation does not depend on what level of temperature is present. There is not a statistically significant interaction between altitude/generation and temperature . (P = 0.297)

Power of performed test with alpha = 0.0500: for altitude/generation : 0.198

Power of performed test with alpha = 0.0500: for temperature : 0.102

Power of performed test with alpha = 0.0500: for altitude/generation x temperature : 0.107

Least square means for altitude/generation :

**Group Mean SEM**

HA F0 2.831 0.0457

HA F1 2.903 0.0444

LA F0 2.969 0.0437

LA F1 2.869 0.0443

Least square means for temperature :

**Group Mean SEM**

19 2.847 0.0381

25 2.897 0.0399

28 2.935 0.0377

Least square means for altitude/generation x temperature :

**Group Mean SEM**

HA F0 x 19 2.783 0.0778

HA F0 x 25 2.810 0.0816

HA F0 x 28 2.899 0.0778

HA F1 x 19 2.754 0.0745

HA F1 x 25 2.906 0.0816

HA F1 x 28 3.048 0.0745

LA F0 x 19 3.012 0.0745

LA F0 x 25 2.976 0.0778

LA F0 x 28 2.918 0.0745

LA F1 x 19 2.838 0.0778

LA F1 x 25 2.896 0.0778

LA F1 x 28 2.873 0.0745
