# Supplementary material for: Local Adaptation to Altitude Underlies Divergent Thermal Physiology in Tropical Killifishes of the Genus Aphyosemion
Source: PLoS One. 2013 Jan 22;8(1):e54345. doi: 10.1371/journal.pone.0054345 (PMC3551936; doi:10.1371/journal.pone.0054345)
Supplement: Text S12 — One Way Analysis of Variance comparing appetite among 2 high altitude and 2 low altitude species. (DOC) [file pone.0054345.s012.doc]

**Supporting Information 12**

**One Way Analysis of Variance comparing appetite among 2 high altitude and 2 low altitude species**

Dependent Variable: appetite (% body mass consumed)

**Group Name N Missing Mean Std Dev SEM**

Low altitude

A. ahli F0 19 12 0 15.511 4.434 1.280

A. ahli F0 25 10 0 14.477 5.609 1.774

A. ahli F0 28 12 0 15.279 5.890 1.700

A. splendopleure F1 19 10 0 18.218 7.327 2.317

A. splendopleure F1 25 12 0 18.619 11.675 3.370

A. splendopleure F1 28 11 0 23.567 9.535 2.875

High altitude

A. exiguum F0 19 10 0 19.282 9.779 3.092

A. exiguum F0 25 10 0 14.096 7.442 2.353

A. exiguum F0 28 12 0 18.226 7.240 2.090

A. cameronense F1 19 12 0 14.432 7.434 2.146

A. cameronense F1 25 11 0 18.994 9.270 2.795

A. cameronense F1 28 12 0 17.483 8.113 2.342

**Source of Variation DF SS MS F P**

Between Groups 11 911.059 82.824 1.279 0.244

Residual 122 7899.764 64.752

Total 133 8810.824

The differences in the mean values among the treatment groups are not great enough to exclude the possibility that the difference is due to random sampling variability; there is not a statistically significant difference (P = 0.244).

Power of performed test with alpha = 0.050: 0.151

The power of the performed test (0.151) is beLAw the desired power of 0.800.

Less than desired power indicates you are less likely to detect a difference when one actually exists. Negative results should be interpreted cautiously.
